# Comprehensive bioinformatics analysis of human cytomegalovirus pathway genes in pan-cancer

**DOI:** 10.1186/s40246-024-00633-5

**Published:** 2024-06-17

**Authors:** Tengyue Yan, Xianwu Pang, Boying Liang, Qiuxia Meng, Huilin Wei, Wen Li, Dahai Liu, Yanling Hu

**Affiliations:** 1 Collaborative Innovation Centre of Regenerative Medicine and Medical Bioresource Development and Application Co-constructed by the Province and Ministry, Guangxi Medical University, Nanning, Guangxi 530021 China; 2 Guangxi Zhuang Autonomous Region Center for Disease Control and Prevention, Nanning, 530028 China; 3 Department of Immunology, School of Basic Medical Sciences, Guangxi Medical University, Nanning, China; 4 School of Information and Managent, Guangxi Medical University, Nanning, China; 5 School of Institute of Life Sciences, Guangxi Medical University, Nanning, China; 6 Department of Biochemistry and Molecular Biology, School of Basic Medicine, Guangxi Medical University, Nanning, China; 7 School of Medicine, Foshan University, Foshan, Guangdong 528000 People’s Republic of China

**Keywords:** Human cytomegalovirus pathway, Pan-cancer, Immune infiltration, Tumor mutation burden, Bioinformatics

## Abstract

**Background:**

Human cytomegalovirus (HCMV) is a herpesvirus that can infect various cell types and modulate host gene expression and immune response. It has been associated with the pathogenesis of various cancers, but its molecular mechanisms remain elusive.

**Methods:**

We comprehensively analyzed the expression of HCMV pathway genes across 26 cancer types using the Cancer Genome Atlas (TCGA) and The Genotype-Tissue Expression (GTEx) databases. We also used bioinformatics tools to study immune invasion and tumor microenvironment in pan-cancer. Cox regression and machine learning were used to analyze prognostic genes and their relationship with drug sensitivity.

**Results:**

We found that HCMV pathway genes are widely expressed in various cancers. Immune infiltration and the tumor microenvironment revealed that HCMV is involved in complex immune processes. We obtained prognostic genes for 25 cancers and significantly found 23 key genes in the HCMV pathway, which are significantly enriched in cellular chemotaxis and synaptic function and may be involved in disease progression. Notably, CaM family genes were up-regulated and AC family genes were down-regulated in most tumors. These hub genes correlate with sensitivity or resistance to various drugs, suggesting their potential as therapeutic targets.

**Conclusions:**

Our study has revealed the role of the HCMV pathway in various cancers and provided insights into its molecular mechanism and therapeutic significance. It is worth noting that the key genes of the HCMV pathway may open up new doors for cancer prevention and treatment.

**Supplementary Information:**

The online version contains supplementary material available at 10.1186/s40246-024-00633-5.

## Background

Cancer, the predominant cause of mortality worldwide, accounts for approximately 19 million new diagnoses and close to 10 million fatalities annually [1]. Intriguingly, a noticeable shift towards a younger demographic in cancer incidence has been observed in recent years [2]. Confronted with the relentless escalation in both cancer incidence and mortality, mitigating this burden has emerged as a cardinal objective within the global public health sphere.

Human Cytomegalovirus (HCMV), a β-herpesvirus ubiquitous in the human population, exhibits a high infection rate of up to 90% [3, 4]. HCMV infection is prevalent worldwide, with susceptibility patterns differing between developing and developed countries. In developing nations, young people are more susceptible to HCMV, whereas in developed countries, the elderly are at a higher risk of infection [5]. Its main transmission occurs through saliva, semen, urine, breastfeeding, placental transfer, blood and organ transplantation [6, 7]. Characterized by a genome of 235 kbp, it stands out as the largest among all herpes viruses, featuring a double-stranded DNA structure, and encode at least 700 open reading frames [8, 9]. Most of the genetic products are closely related to HCMV infection and prevalence [4].

The majority of people infected with primary HCMV are asymptomatic, and only a small number of people will develop mononucleosis syndrome after infection, with fever, sweating, abnormal liver function and discomfort and other symptoms [10, 11]. The natural course of HCMV infection is complex, with primary infection occurring when an immune compromised individual is first infected, followed by an incubation period. When the infected person is exposed to HCMV again, it will be repeated infection, which is called reinfection. HCMV persists throughout the host’s lifetime, and most people carry it in a latent state, but there is also a risk of it being reactivated [12–14]. In addition, HCMV can also cause a variety of diseases such as systemic lupus erythematosus, systemic sclerosis, pneumonia, atherosclerosis, mental disorders and so on [15–19]. Compare this with the diseases mentioned above, as the apoptosis disorder induced by HCMV infection is more closely related to cancer and prompts biological responses that closely mimic those supporting chronic inflammation, leukocyte dysfunction, angiogenesis, and wound healing, potentially making it a promoter of a malignant tumor [4, 20, 21]. Numerous studies have found that HCMV may be associated with various types of cancer, such as hepatocellular carcinoma [22], breast cancer [23], gastric cancer [24], cervical cancer [25], colorectal cancer [26], ovarian cancer [27], prostate cancer [28], lymphoma [29], and glioblastoma [30]. Importantly, due to the host immune evasion mechanism of HCMV, it profoundly influences the development of the tumor microenvironment. This influence not only alters the tumor microenvironment but is also associated with poor prognosis, metastasis, and drug resistance [14, 31]. However, the specific molecular mechanism of how HCMV is involved in the regulation of tumour development remains to be further elucidated.

In this study, we comprehensively analyzed the expression of HCMV pathway genes across cancers using the Cancer Genome Atlas (TCGA) and The Genotype-Tissue Expression (GTEx) databases. Importantly, we revealed the relationships between HCMV pathway gene expression and prognosis, genomic mutations, tumor microenvironment (TME), and chemo- and immunotherapy drug sensitivity. Additionally, through comprehensive bioinformatics analysis, we identified hub genes in the HCMV pathway that may play crucial roles in regulating cancer progression.

## Methods

### Data collection and screen

We obtained pan-cancer TCGA and GTEx transcriptomic expression data (log2(TPM + 0.001)), as well as clinical data, from the UCSC Xena database (https://xena.ucsc.edu/). Additionally, we acquired pan-cancer mutation data from the TCGA database (https://portal.gdc.cancer.gov/). Subsequently, we procured genes associated with the Human cytomegalovirus infection pathway from the KEGG database (https://www.genome.jp/kegg/). In clinical data, we first eliminated missing values and then matched the tumor samples accordingly. Subsequently, we extracted pathway genes and further analyzed the differential expression between various cancerous tumors and normal tissues using the Wilcoxon test. All analyses are based on R (version: 4.2.2). We established the following inclusion and exclusion criteria: the number of normal controls for each cancer must be greater than or equal to 5, and a P-value less than 0.05 was considered statistically significant.

### Differential expression analysis

Limma-Voom: the model consistently performed well on various benchmark datasets, providing a reasonable balance between error discovery (FDR) and recall rates, which is comparable to or better than that of count-based RNA-seq methods [32, 33]. Consequently, we employed this method to further compare the gene expression levels in normal and cancer tissues. We set the threshold values at adjust P Value < 0.05 and |logFC| ≥ 1, thereby identifying genes with significant differential expression.

### Immune cell infiltration

For the analysis of immune infiltration, we employed the CIBERSORT R package, setting perm to 1000. This tool, extensively utilized for investigating the proportions of 22 subtypes of human immune cells, leverages a machine learning technique known as Support Vector Regression (SVR) to enhance deconvolution performance through an amalgamation of feature selection and robust mathematical optimization techniques, notably outperforming other methods in terms of accuracy when it comes to resolving closely related cell subsets and mixtures with unknown cell types [34].

### Tumor microenvironment

ESTIMATE: A methodology that utilizes gene expression features to infer the proportion of stromal and immune cells in tumor samples [35]. We employed the “ESTIMATER” R package to calculate the stromal score and immune score in each cancer, further estimating the tumor purity for each type of cancer (platform select “Affymetrix”).

### Prognostic related gene screening

A univariate Cox analysis was performed on the differential genes of each cancer. To prevent the loss of some important genes, genes with a P-value less than 0.2 were screened. To minimize overfitting risk, the Lasso regression and XGboost were then used for the feature selection. We performed feature selection and shrinkage using the “glmnet” R package. The penalty parameter (λ) of the model was determined by conducting ten-fold cross-validation and following the minimum criterion, which corresponds to the λ value of the minimum likelihood deviation [36]. Meanwhile using the “XGBoost” R package, we set ‘survival: cox’ as the objective function and ‘cox-nloglik’ as the evaluation metric, trained the model for 100 rounds at a 0.1 learning rate with the L1 regularization hyperparameter alpha set to 0.5 to prevent overfitting. Finally, a multivariate Cox analysis was performed, selecting genes with P < 0.05 as prognostic genes [37]. Patients were divided into high-risk and low-risk groups based on the median of the risk scores, followed by survival analysis using the “survminer” package in R. The “timeROC” package in R was utilized to conduct time-dependent receiver operating characteristic (ROC) curve analysis to evaluate the prognostic predictive performance of the gene features for prognosis over a 3-year period [38].

### Tumor mutation burden

Tumor Mutation Burden (TMB), characterized as the aggregate count of somatic coding inaccuracies, base substitutions, and insertion or deletion mutations identified per million bases of DNA, serves as an effective estimator for both mutational and neoantigen loads [39]. The “maftools” R-package integrates standard analysis and visualisation modules into a single pipeline by implementing well-established statistical and computational methods, requiring only a single and uniform input data format for the process from analysis to visualisation to annotation [40]. We further used this R package to calculate the mutations in prognosis-related genes for each cancer.

### Screening for hub genes

The gene interaction network was established utilizing the STRING database (https://string-db.org/). Choose the highest confidence (0.900) as the minimum required interaction score. Further use Cytoscape software to visualize the connections between genes. For further study, after protein-protein network analysis, the MCODE plug-in was performed to screen hub genes, whose degree cutoff was = 2, node score cutoff was = 0.2, k core was = 2, and maximum depth was = 100 [41].

### GO enrichment analysis of hub gene

To ascertain the functions of the identified hub genes, a Gene Ontology (GO) analysis was conducted, encompassing enrichment of Biological Process (BP), Cellular Component (CC), and Molecular Function (MF) [42]. Further analysis was conducted using the “clusterProfiler”, “enrichplot” and “Goplot” R packages, with a p.adjust value of less than 0.05 set as the critical threshold.

### Drug sensitivity analysis

The Genomics of Drug Sensitivity in Cancer (GDSC) database, as the largest public resource for information on drug sensitivity in cancer cells and molecular markers of drug response, amalgamates extensive drug sensitivity and genomic datasets to expedite the discovery of novel therapeutic biomarkers for cancer treatment [43]. And subsequently, we utilized GSCA (http://bioinfo.life.hust.edu.cn/GSCA/#/) [44], a tool incorporating data from the GDSC database, to investigate the association between hub genes and drug sensitivity.

## Results

### Data set screening and preliminary analysis

**Fig. 1:**
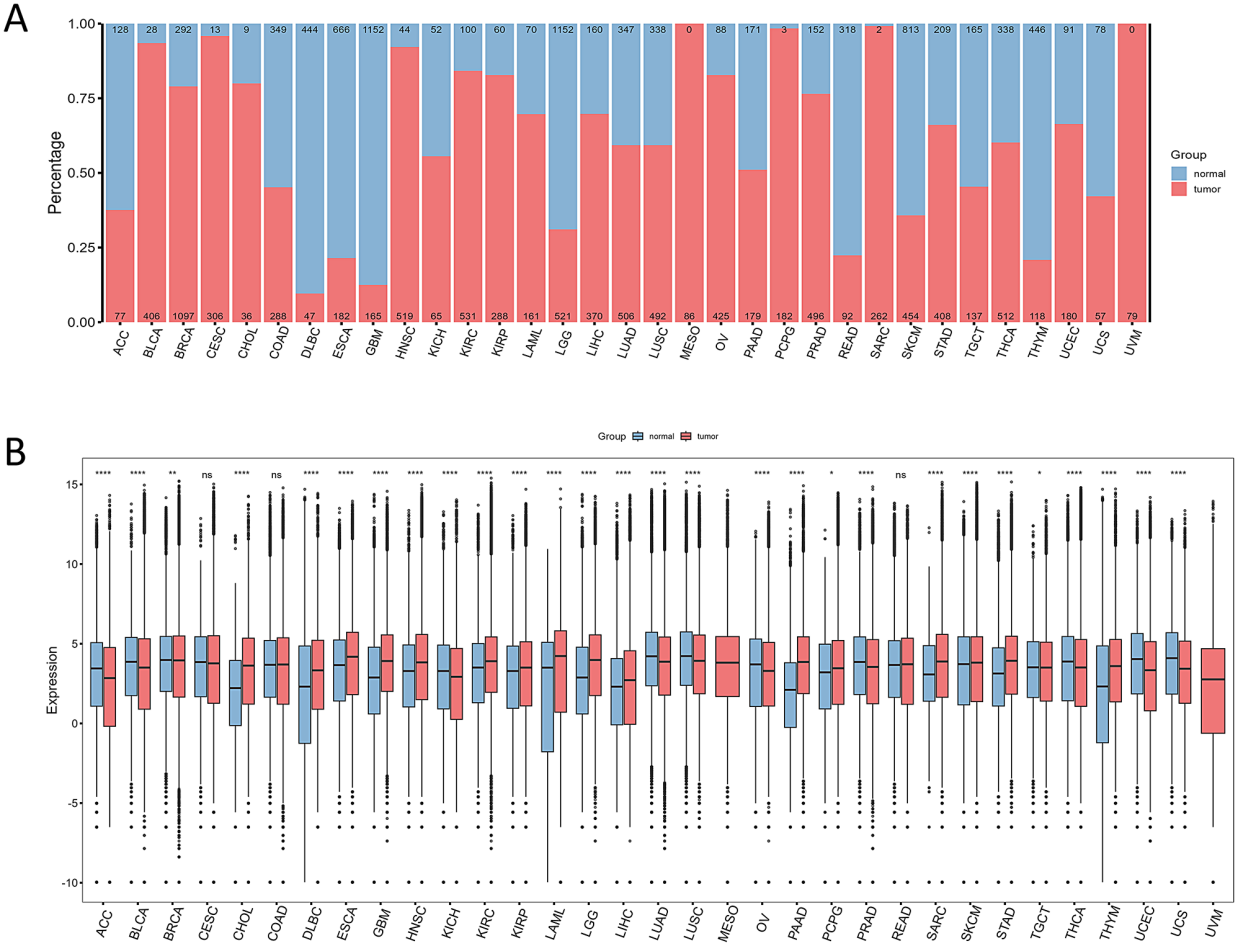
Overview of Sample Composition and Gene Expression Differences in Various Cancers.(**A**) The distribution of normal and tumor tissue samples across different cancer types: The horizontal axis represents different types of cancer, and samples are distinguished by color: blue for normal tissue and red for tumor tissue. For each cancer type, the counts of normal samples are indicated at the top, while those of tumor samples are indicated at the bottom. (**B**) The differences between normal and tumor samples across various cancers: Normal and tumor samples are depicted in blue and red, respectively. The horizontal axis represents different types of cancer, while the vertical axis indicates the level of gene expression. The symbols “*”, “**”, “***”, “****” and “ns” correspond to p < 0.05, p < 0.01, p < 0.001, p < 0.0001, and non-significance, respectively. The lack of any markers signifies the absence of normal controls for that type of cancer (MESO, UVM)

We downloaded samples of 33 types of cancer from public database. After screening, we obtained a total of 18,002 samples, including 8,278 normal samples and 9,724 tumor samples. The sample information for each cancer type is shown in Fig. 1A. Using the Wilcoxon test, we excluded 7 types of cancer. Ultimately, we identified 26 types of cancer for further analysis. It’s noteworthy that gene expression in tumor tissues surpasses that in normal tissues for most cancers, as depicted in Fig. 1B.

### Differential gene recognition

**Fig. 2:**
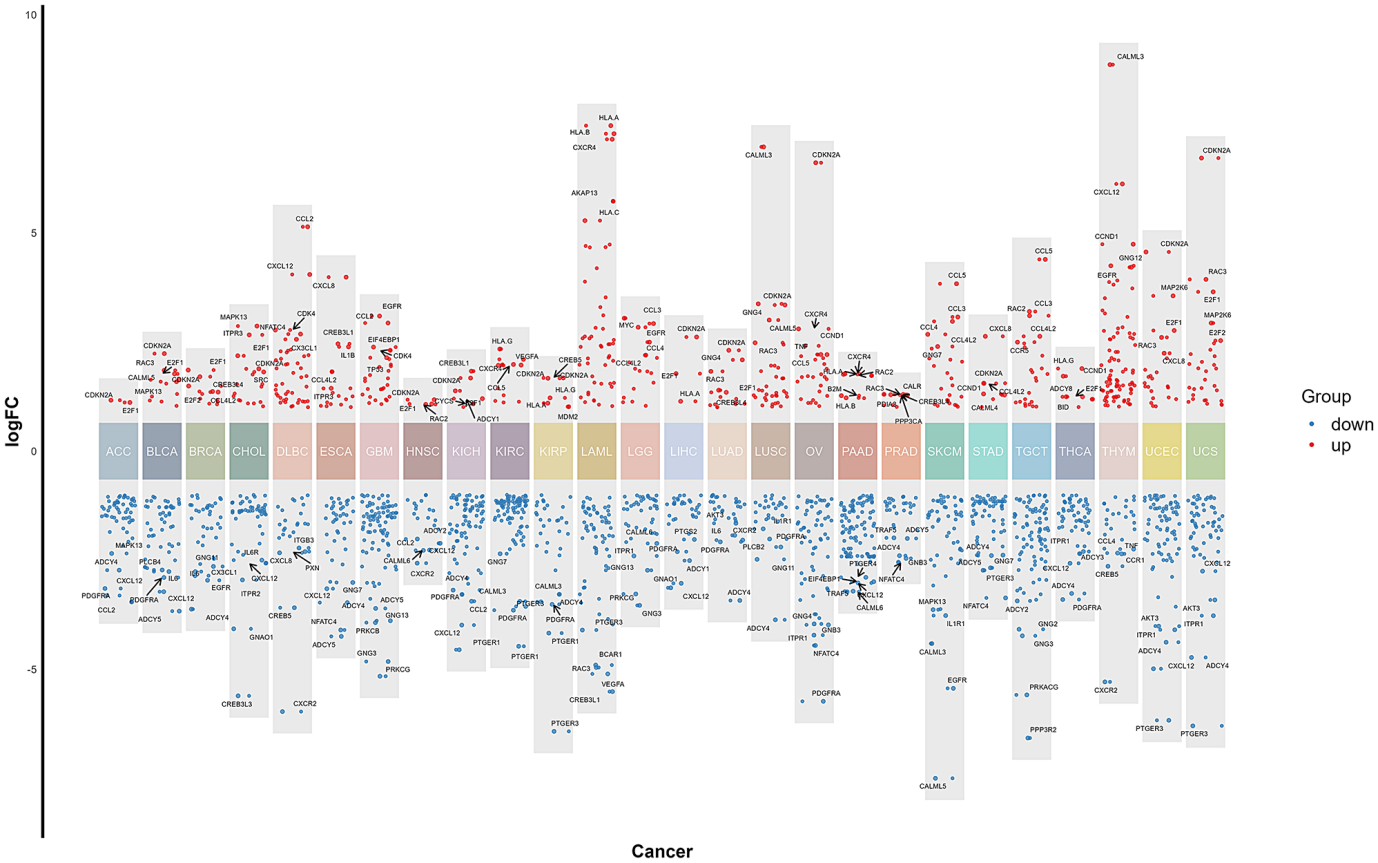
The differential gene expression across various cancer types (from left to right ACC, BLCA, BRCA, CHOL, DLBC, ESCA, GBM, HNSC, KICH, KIRC, KIRP, LAML, LGG, LIHC, LUAD, LUSC, OV, PAAD, PRAD, SKCM, STAD, TGCT, THCA, THYM, UCEC, UCS). The y axis shows the fold changes in gene expression. Gene expression levels relative to different color blocks are displayed in varying colors, with red representing up-regulated genes and blue representing down-regulated genes

Differential expression analysis identified a total of 1,853 differentially expressed genes, including 506 up-regulated and 1,347 down-regulated genes. The top 5 up- and down-regulated genes across the cancer samples were displayed (Fig. 2). The complete list of differentially expressed genes for each cancer sample is provided in Supplementary Table S1. Additionally, we have generated clustering heatmaps for each type of cancer to visually illustrate the differences in gene expression. The corresponding images can be found in Figure S1.

In addition, we analyzed the significant functional differences and commonalities of differential genes across various cancer types using GO enrichment analysis (Figure S2). For example, in adrenocortical carcinoma (ACC), genes primarily function in response to peptide hormones, highlighting their importance in regulating hormone balance and cell signaling. In cholangiocarcinoma (CHOL), genes play a significant role in peptidyl-serine phosphorylation, indicating their critical role in protein modification and signal transduction. In liver hepatocellular carcinoma (LIHC), genes are crucial in responding to tumor necrosis factor, underscoring their involvement in inflammatory responses and immune regulation. In breast invasive carcinoma (BRCA), genes are particularly active in muscle cell proliferation, indicating their role in cell growth and tissue development… Notably, differential genes across multiple cancer types are involved in leukocyte migration, cellular response to abiotic stimuli, and regulation of vasculature development. This suggests that these genes play universally important roles in regulating immune responses, adapting to environmental changes, and promoting angiogenesis.

### Differences in immune cell infiltration in different cancer samples

**Fig. 3:**
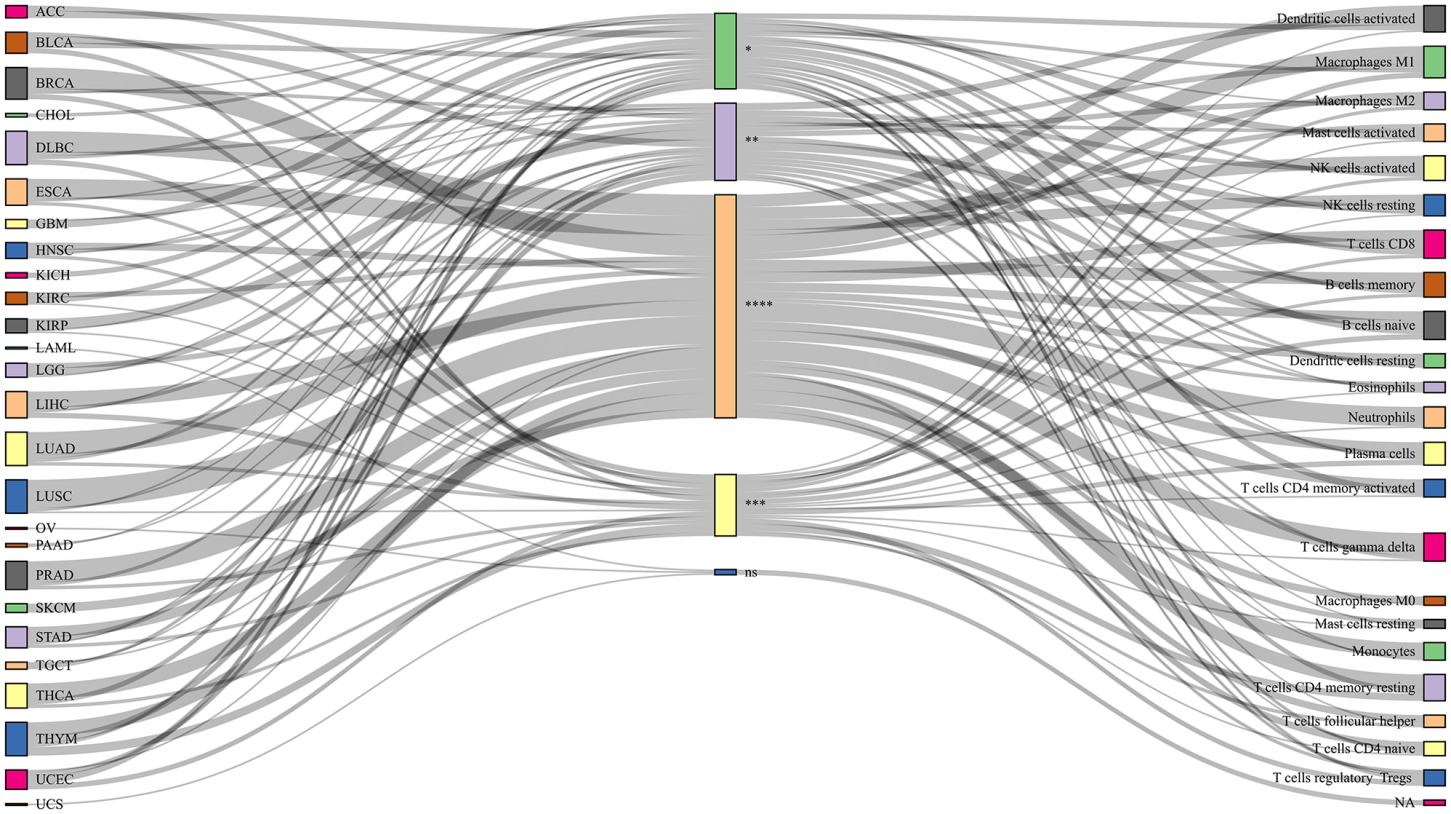
Immune cell infiltration in various types of cancer: The squares on the left represent different cancer types, the middle squares represent salience scores, and the squares on the right represent distinct immune cell populations. The symbols “*”, “**”, “***”, “****” and “ns” correspond to p < 0.05, p < 0.01, p < 0.001, p < 0.0001, and non-significance

An in-depth analysis of immune cell infiltration in a variety of cancer samples was performed using CIBERSORT, after which immune subpopulations with P-values less than 0.05 were screened out and correlation heat maps were used to show the relationship between differential genes and these subpopulations (Figure S3), the proportion of cell subpopulations in each cancer is shown in Figure S4. To further explore the differences in immune cells in tumor and normal samples, we used the Wilcoxcon rank sum test for comparison. The results showed that immune infiltrating cells in the pathway mainly included T cells, macrophages, NK cells, B cells, dendritic cells, plasma cells, monocytes, and neutrophils. To visualize these findings more intuitively, we visualized the results using the “networkD3” R package (Fig. 3). These data provide us with strong evidence that these immune cell subpopulations may play a key role in the pathogenesis of various cancers.

### Tumour microenvironment score

**Fig. 4:**
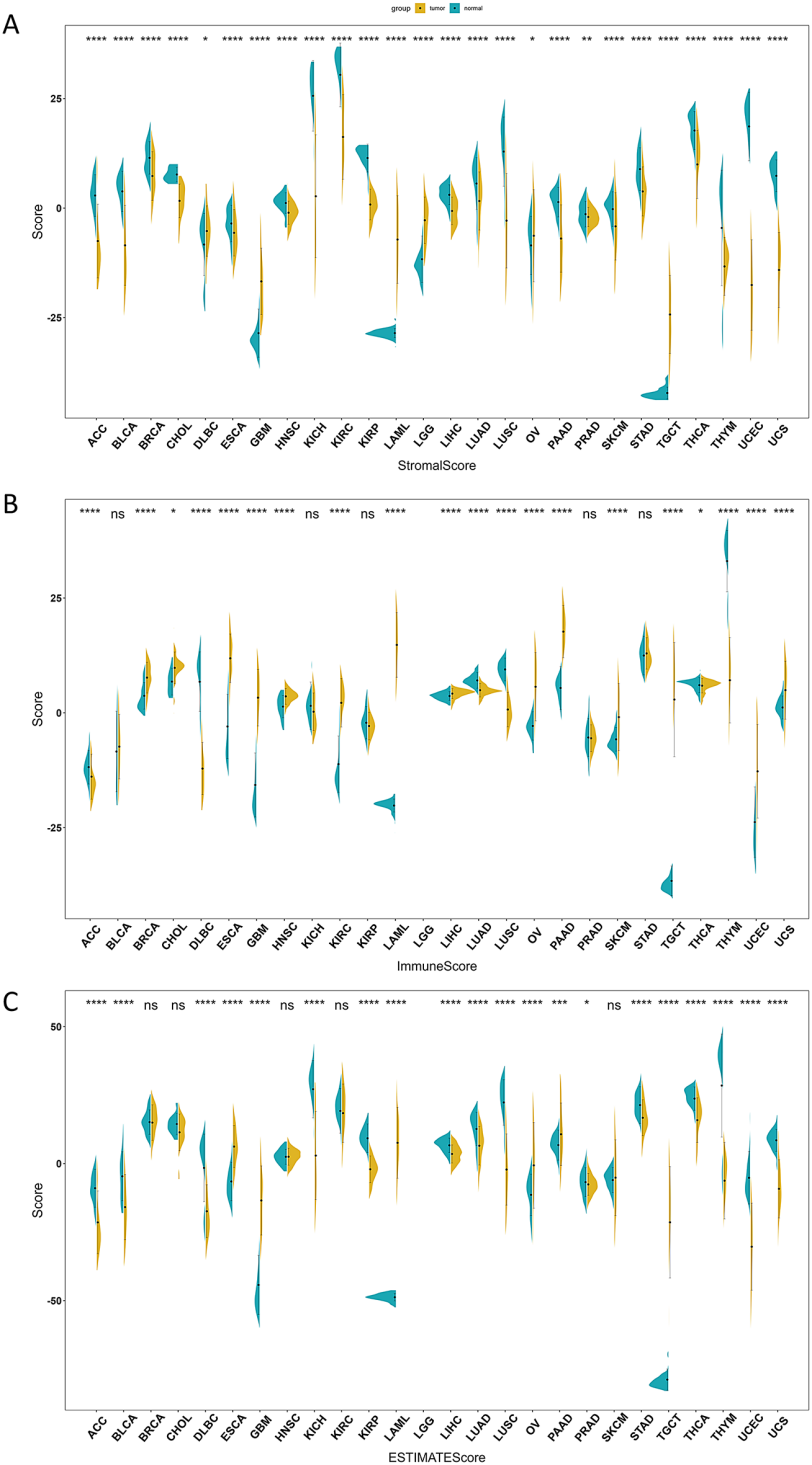
Assessing the tumor microenvironment via ESTIMATE algorithm: The horizontal axis represents different cancer types, and the vertical axis represents the scores. Normal samples are represented in blue, while tumor samples are indicated in yellow. The symbols “*”, “**”, “***”, “****” and “ns” correspond to p < 0.05, p < 0.01, p < 0.001, p < 0.0001, and non-significance. (**A**) Distribution of stromal scores across various cancers assessed by ESTIMATE approach. (**B**) Distribution of immune scores across multiple cancer types calculated by ESTIMATE algorithm. (**C**) Estimate scores reflecting tumor purity determined by ESTIMATE method

Using the ESTIMATE algorithm, we analyzed 26 cancer types and obtained stromal scores, immune scores and estimate scores for each cancer except LGG, which only had stromal scores available. The estimate score is calculated as the sum of stromal and immune scores [35]. As shown in Fig. 4, immune scores were increased while stromal and estimate scores were decreased across most cancer cohorts. Additionally Stromal scores, immune scores and estimate scores were all negatively correlated with tumor purity, with the estimate score showing the strongest negative correlation (Figure S5). This implies that immune cells may play an increasingly important role within the tumor microenvironment of these cancers.

### Prognostic related gene screening

**Fig. 5:**
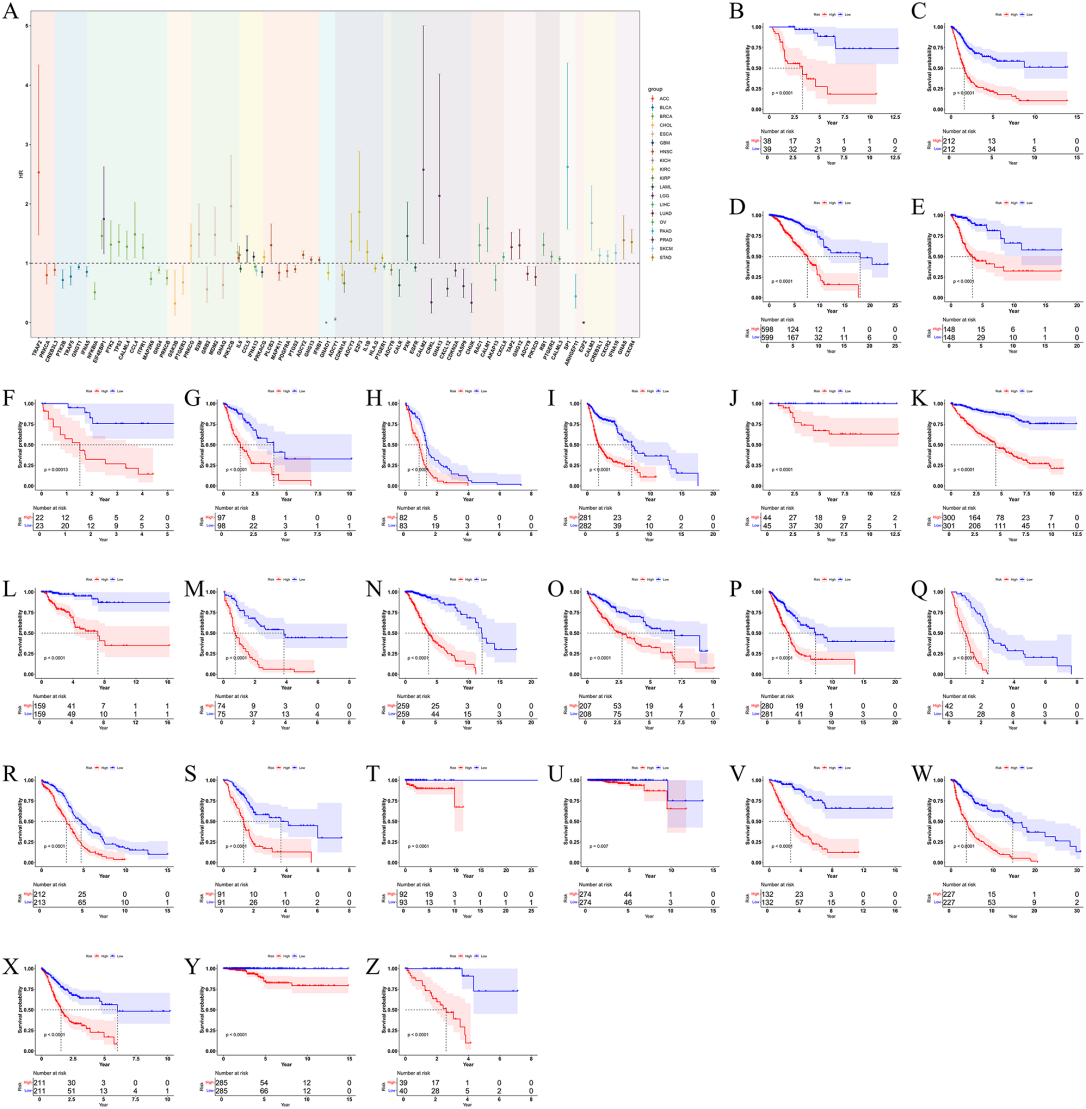
Identification and validation of prognostic gene signatures for cancers. (**A**) Forest plots of partial prognostic genes for each cancer: Different colors represent different cancer types, the horizontal axis represents different genes, the vertical axis represents the hazard ratio (HR) (dashed line represents HR = 1) and the line segments in the figure represent confidence intervals (CI). Kaplan-Meier curves compared the prognostic situations of prognostic genes in (**B**) ACC, (**C**) BLCA, (**D**) BRCA, (**E**) CHOL, (**F**) DLBC, (**G**) ESCA, (**H**) GBM, (**I**) HNSC, (**J**) KICH, (**K**) KIRC, (**L**) KIRP, (**M**) LAML, (**N**) LGG, (**O**) LIHC, (**P**) LUAD, (**Q**) LUSC, (**R**) OV, (**S**) PAAD, (**T**) PRAD, (**U**) SKCM, (**V**) STAD, (**W**) THCA, (**X**) THYM, (**Y**) UCEC, (**Z**) UCS. Red lines represented high-risk group, blue represented low-risk group

**Fig. 6:**
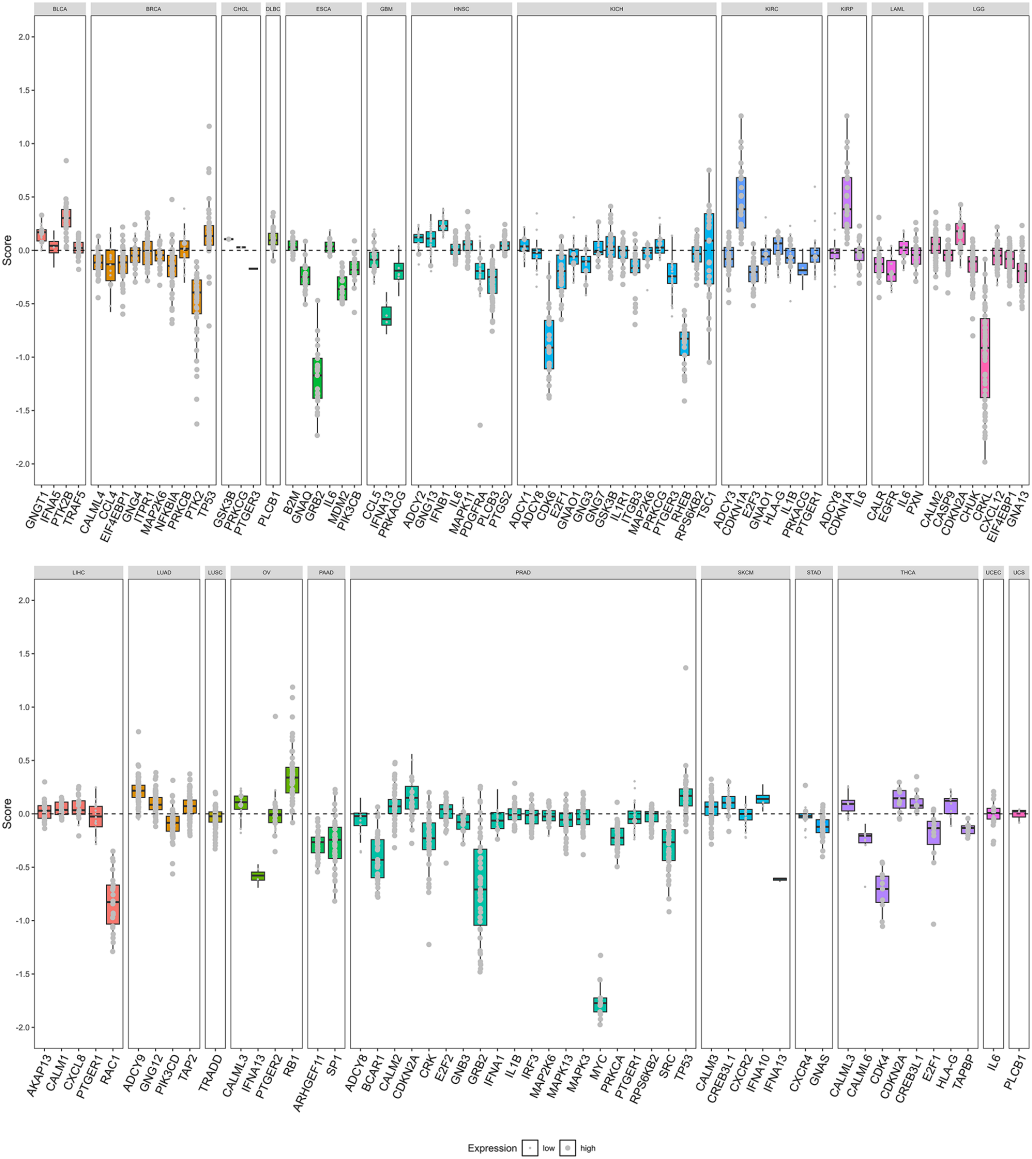
Validation of pan-cancer prognostic genes in CCLE: Validation of pan-cancer prognostic genes in CCLE: Each panel represents a different cancer type, with the x-axis denoting different genes, and the y-axis representing The Chronos dependency score. The size of the points within the boxplot corresponds to the gene’s expression level in cells (log2(TPM + 1))

After screening 26 cancer-related genes by univariate analysis, we further screened them by LASSO regression method and successfully obtained 20 prognostic genes associated with cancer. For the six cancers (LUSC, DLBC, TGCT, THYM, UCEC, UCS) that failed the LASSO screening, we re-screened them using the XGboost method, and only TGCT was excluded (P > 0.05), and finally we obtained prognostic-associated genes for these cancers (Table S2). We showed some of the genes with prognostic value for each cancer (Fig. 5A). The survival curves of different cancers with high and low prognostic risks were clearly separated, and the prognostic assessment models constructed using these genes could effectively distinguish the prognostic status of different cancer samples (Fig. 5B-Z). These models were validated by ROC curves and showed reliable predictive efficacy (Figure S6). To further confirm the importance of these genes in the development of cancer and their clinical significance regarding expression levels, we utilized data from The Cancer Cell Line Encyclopedia (CCLE) project for external validation. The CCLE is a comprehensive resource that includes genomic and drug response data from over 1000 cancer cell lines of various tissue types, offering a valuable asset for cancer biology and precision medicine research [45]. Our analysis revealed that most genes exhibit a Chronos dependency score of less than zero, suggesting their potential role in cancer progression [46]. Furthermore, the majority of these genes show high expression levels in cancer cells (Fig. 6), suggesting their significance in specific cellular contexts.

### Tumor mutation burden

**Fig. 7:**
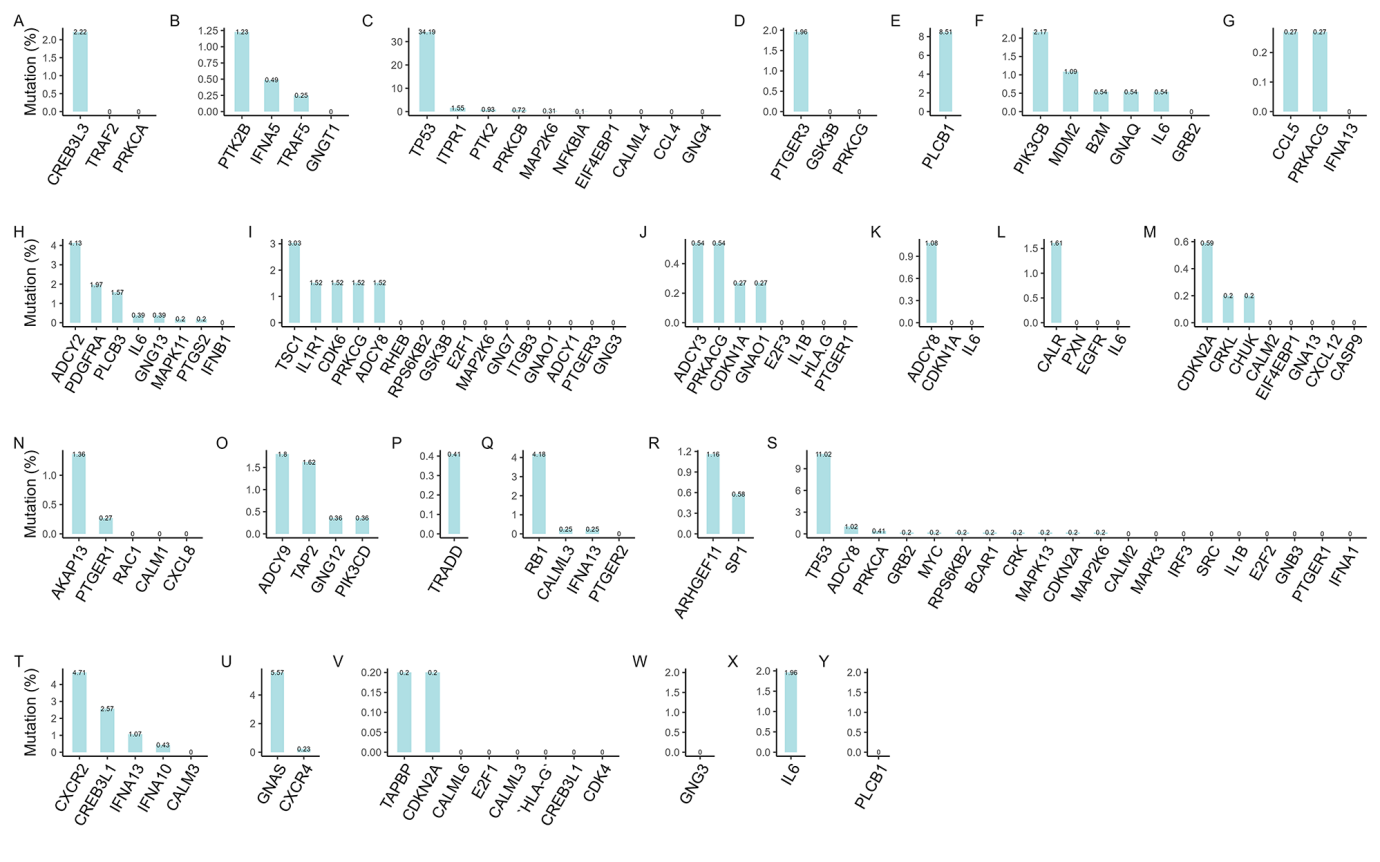
Mutations of different cancer prognostic genes. Prognostic gene mutation details in (**A**) ACC, (**B**) BLCA, (**C**) BRCA, (**D**) CHOL, (**E**) DLBC, (**F**) ESCA, (**G**) GBM, (**H**) HNSC, (**I**) KICH, (**J**) KIRC, (**K**) KIRP, (**L**) LAML, (**M**) LGG, (**N**) LIHC, (**O**) LUAD, (**P**) LUSC, (**Q**) OV, (**R**) PAAD, (**S**) PRAD, (**T**) SKCM, (**U**) STAD, (**V**) THCA, (**W**) THYM, (**X**) UCEC, (**Y**) UCS: The horizontal axis represents genes, and the vertical axis represents mutation rates

Utilizing “maftools” R package, we systematically analyzed the mutation profiles of various cancer prognostic genes. Of the 131 prognostic-related genes examined, mutations were identified in 72 (54.96%), whereas the other 59 (45.04%) did not harbor any mutations (Fig. 7A-Y). Further mutation classification of these 72 mutated genes revealed a predominance of missense mutations in the majority of them. The detailed mutation spectra for each individual gene are illustrated in Figure S7.

### Hub gene and its functional enrichment analysis

**Fig. 8:**
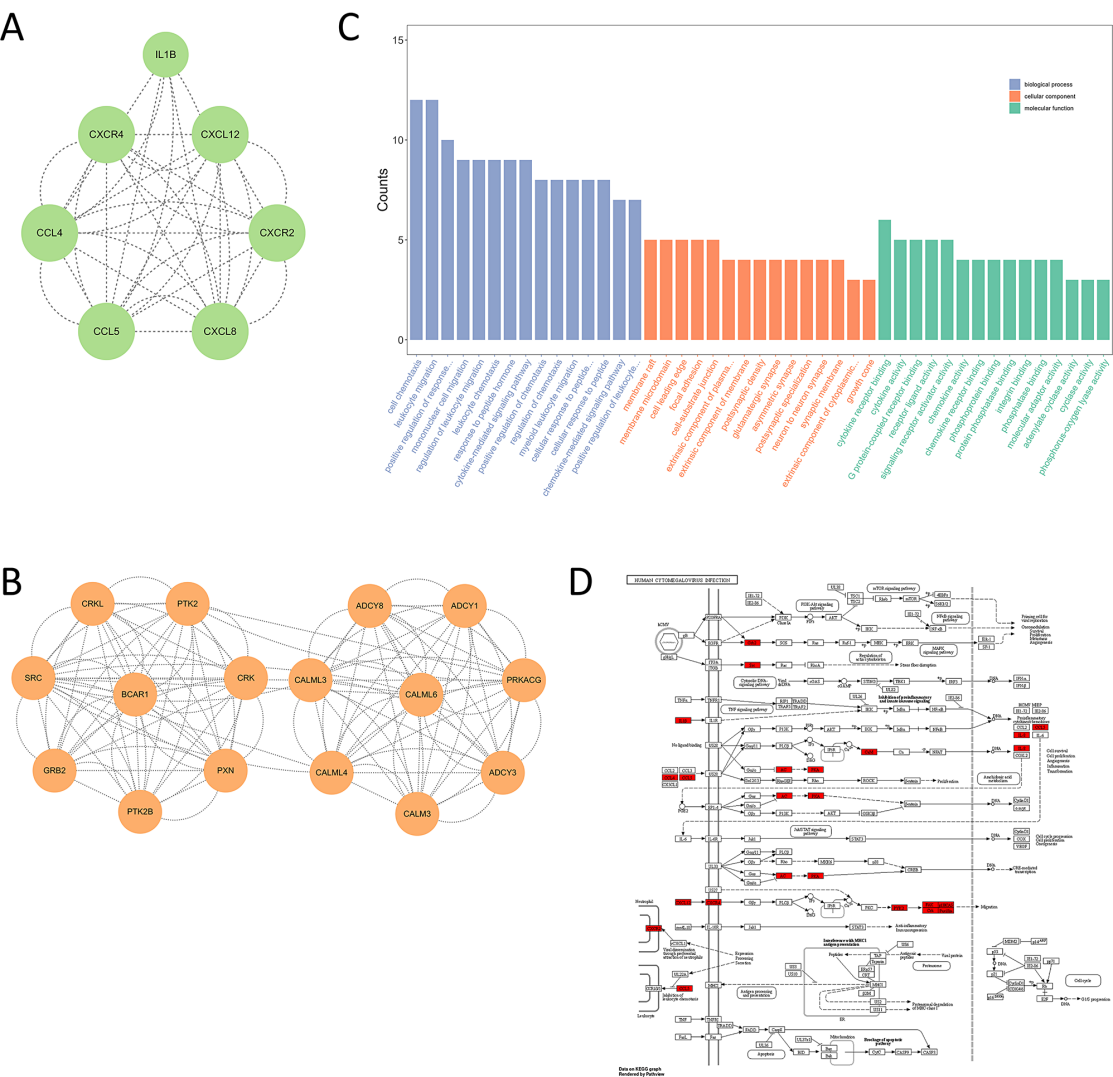
Integrated Analyses of HCMV Pathway and Hub Genes. (**A**, **B**) Two significant subnetworks of PPI network. (**C**) GO enrichment analysis of Hub genes: The horizontal axis represents the potential functions enriched, while the vertical axis indicates the number of enrichments (from left to right, they are Biological Process (BP) in purple, Cellular Component (CC) in orange, and Molecular Function (MF) in green). (**D**) Pathway localization of hub genes in HCMV pathway

After constructing the Protein-Protein Interaction (PPI) network, MCODE analysis was further used to screen modules with scores ≥ 6, highlighting the most interconnected hub genes implicated in the HCMV pathway. A total of 23 hub genes were obtained, including CALML4, GRB2, CALML3 etc. The hub gene interaction network is shown in Fig. 8A-B. GO enrichment analysis revealed that these hub genes were significantly enriched in 803 BP, 47 CC and 97 MF. The enriched BP were primarily related to cell chemotaxis and response to peptides; the CC were mostly membrane-bound and synapse-related; the MF included receptor ligand binding activities and enzyme activities (Fig. 8C), underscore the multifaceted biological implications of these genes. In addition, we visualized key genes in the HCMV pathway using the ‘pathview’ R package [47]. The results showed that most of the genes had protein-molecular interactions in the pathway. Specifically, the expression of CaM (CALML4, CALM3, CALML6) was promoted, while AC (ADCY8, ADCY3) was inhibited in the pathway (Fig. 8D), and potentially impacting the HCMV’s pathophysiological mechanisms.

### Analysis of the correlation between hub gene expression levels and drug sensitivity

**Fig. 9:**
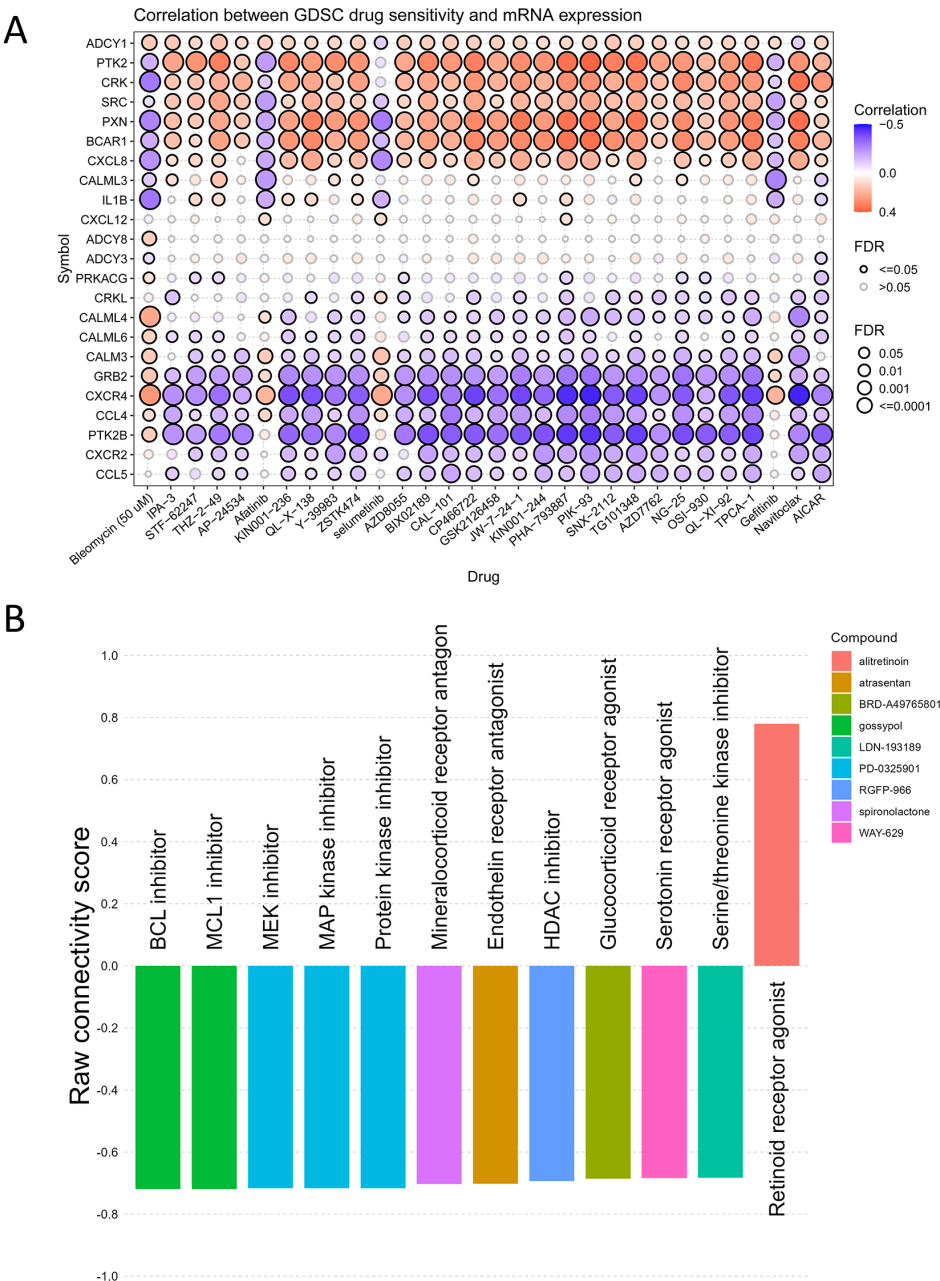
Integration of GDSC Analysis and CMap Validation Reveals Potential Drug Targets Associated with Hub Genes. (**A**) Gene expression-drug sensitivity correlations: Red indicates positive correlation and blue indicates negative correlation. Bubble size positively corresponds to FDR significance, with black outline highlighting correlations meeting FDR < = 0.05 threshold. (**B**) CMap validation identifies potential drug targets: The columns of various colors represent the actions of different compounds, with corresponding labels denoting the potential targets of action. The vertical axis depicts the connectivity score

We investigated the correlation between the expression of HCMV pathway hub genes and drug sensitivity profiles in the GDSC database. The results showed that the expression levels of ADCY1, PTK2, CRK, SRC, PXN, BRCA1, CXCL8 are mostly positively correlated with IPA-3, STF-62,247, THZ-2-49, AP-24,534, KIN001-236, QL-X-138, Y-39,983, ZSTK474, AZD8055, BX02189, CAL-101, CP466722, GSK2126458, JW-7-24-1, KIN001-244, PHA-793,887, PIK-93, SNX-2112, TG101348, AZD7762, NG-25, OSI-930, QL-XI-92, TPCA-1, Navitoclax and AICAR, these findings suggest that elevated expression of these genes may serve as a biomarker for predicting response to specific drugs, potentially guiding more tailored treatment strategies. The expression levels of ADCY1, PTK2, CRK, SRC, PXN, BRCA1, and CXCL8 were mostly negatively correlated with sensitivity to bleomycin (50 µM), afatinib, cerutinib, and gefitinib. By contrast, the expression levels of CALML4, CALML6, CALM3, GRB2, CCR4, RTK2B, CXCR2, CCL5 to the above mentioned is the opposite (Fig. 9A), these may provide new insights into developing combination therapies or alternative treatments to overcome drug resistance. To further validate these findings, we performed additional analyses using the Connectivity Map (CMap), which helped identify the targets of drug action [Fig. 9B]. For instance, the mechanism of action of PD-0325901 is highly similar to that of AP-24,534, AZD8055, and TG101348, as listed in the GDSC database, and all belong to kinase inhibitors. Of note, the results from CMap suggest that drugs may exert their effects by inhibiting or downregulating these target points. This not only strengthens the reliability of our results but also further confirms the potential application of these HCMV pathway hub genes in predicting drug response, demonstrating their significant value in drug response prediction.

## Discussion

In this study, we comprehensively analysed the role of the human cell cytomegalovirus (HCMV) pathway in the development of a variety of cancers by means of bioinformatics methods. We found that the HCMV pathway has a wide range of differentially expressed genes in various cancer samples. Immune infiltration analysis demonstrated that immune cells such as T cells and macrophages play important roles in the tumor microenvironment, providing evidence that HCMV is involved in the regulation of tumorigenesis. Furthermore, 23 pathway key genes were identified, which provide potential targets for the development of HCMV-associated targeted therapies. Overall, our study comprehensively resolved the role of HCMV pathway in the development of various cancers, provided new insights into the relationship between HCMV and tumours, and provided a theoretical basis for the treatment of HCMV-associated tumours.

HCMV invasion induces multiple immune responses. Endothelial cells, dendritic cells, natural killer cells, monocytes, and macrophages become activated in the blood and tissues, producing abundant inflammatory mediators including IL-1β, IL-2, IL-6, TNF-α, and chemokines [48–51]. This process prompts circulating neutrophils to receive signals from antigen-presenting cells at the site of infection and prepare for a further immune response. Concurrently, the activation of local clotting factors also induces platelet aggregation, further enhancing inflammatory processes [52, 53]. Our analysis revealed that differential gene functions across various cancer types, including leukocyte migration, response to abiotic stimulus, and vasculature development regulation, align with the immune responses seen in HCMV invasion. These shared pathways highlight common mechanisms of immune regulation and inflammation critical to both viral infections and cancer progression. Given these findings, it is crucial to explore additional genetic regulatory layers that may influence these processes. DNA methylation, one of the most extensively studied epigenetic modifications, plays a critical role in essential biological processes such as embryonic development, genomic imprinting, and X-chromosome inactivation [54]. Aberrant DNA methylation can alter the cellular microenvironment, affect gene expression patterns, and lead to various pathological conditions, including cancer [55]. Using the MethSurv tool, we explored individual methylation CpG sites in differentially expressed cancer genes [56–58]. For instance, in adrenocortical carcinoma (ACC), we found a significant association between the methylation site cg04721825 in the PRKCA gene (HR: 6.626, 95% CI: 1.561–28.119, P: 0.010349507) and disease risk. Similarly, in bladder cancer (BLCA), the methylation site cg09825327 in the TRAF5 gene (HR: 1.682, 95% CI: 1.136–2.49, P: 0.009355414) was significantly associated with disease risk… These results suggest that variations in methylation sites may play a critical role in cancer development, highlighting an important direction for future research. However, the relationship between HCMV and the host immune system is complex, and once HCMV enters the body, it is able to establish latency in undifferentiated hematopoietic progenitor cells in the bone marrow and then reactivate, leading to a recurrence of the disease [51, 59, 60]. Latently infected monocytes disseminate the virus to various organs, and upon their reactivation, the adaptive immune system activates T helper cells and cytotoxic T cells via T cell receptors while accelerating epigenetic events. This further exacerbates the inflammatory role in pathophysiology, which may precipitate multi-organ diseases, heighten disease risk, and even cause death in severe cases [61–64].

Using the bioinformatics tools CIBERSORT and ESTIMATE, we analyzed the immune cell composition and scoring of 26 cancer types. While the degree and proportion of immune cell infiltration varied across cancers, the infiltration patterns were similar to those of cells involved in innate and adaptive immunity. Fractalkine/CX3C chemokine ligand 1 (CX3CL1) and its receptor CX3CR1 have been found to allow immature dendritic cells to migrate to cancer cells using the expression of their receptor CX3CL1 [65, 66]. The CX3CL1-CX3CR1 axis promotes NK cells to adhere to tumor cells and directly kill cancer cells [67, 68]. Cancers associated with the CX3CL1-CX3CR1 axis include BLCA, STAD, BRCA, GBM, LGG, LIHC, LUAD, LUSC, PAAD, KICH, KIRC, KIRP, OV, PAAD, HNSC, PRAD, TGCT, UCEC, SKCM, etc [69]. . . Results from ESTIMATE revealed high tumor purity across most cancer cohorts, implying a relatively high proportion of tumor cells and low immune activity in the tumor samples. The tumor immune microenvironment represents a highly complex system that is pivotal in driving immunosuppression, distant metastasis, local drug resistance, and response to targeted therapies [70–73]. Moreover, it is closely related to the clinical prognosis of tumor patients [74]. The tumor microenvironment contains multiple immunosuppressive cell types that are induced by cancer-associated fibroblasts, such as M2 macrophages, regulatory T cells and myeloid-derived suppressor cells. These immunosuppressive cells accumulate abundantly within the tumor immune microenvironment (TIME) and play critical roles in promoting immune evasion and suppression [75–77]. , with which our results are also consistent, supporting the hypothesis that enriched immune cells reduce the proportion of tumour cells by enhancing their killing effect on tumour cells and inhibiting their proliferation. We comprehensively analysed the immune infiltration of pan-cancer, which is important for understanding the immune characteristics of tumours and developing corresponding immunotherapy strategies. Future studies can further delve into the interactions of different immune cells in the tumour immune microenvironment to more comprehensively resolve the complexity of the tumour immunoregulatory network.

We identified 25 cancer prognostic genes that are significantly associated with survival (ACC, BLCA, BRCA, CHOL, DLBC, ESCA, GBM, HNSC, KICH, KIRC, KIRP, LAML, LGG, LIHC, LUAD, LUSC, OV, PAAD, PRAD, SKCM, STAD, THCA, THYM, UCEC, UCS), these predictive models of gene composition can effectively distinguish between high and low risk groups of cancer patients. The use of large-scale public databases for biological information mining allows us to efficiently discover these potential cancer prognostic genes [78]. Our findings provide important molecular markers for prognostic assessment and risk stratification of cancer. The study by Anuraga et al. (2021), leveraging multiple database resources such as The Cancer Cell Line Encyclopedia (CCLE) and the Tumor Immune Estimation Resource (TIMER), has successfully identified prognostic biomarkers for breast cancer, contributing valuable insights to the field. Their work not only underscores the efficacy of CCLE in cancer genomics research but also inspired the application of this methodology to pan-cancer analysis, further exploring the commonalities and specificities of molecular characteristics across various cancers [79, 80]. Notably, individual cancer types, such as TGCT, did not show significant prognostic genes in our model, which may be related to the small sample size. In follow-up studies, we will collect more samples to improve statistical efficiency and use experimental techniques to verify the expression and prognostic value of these genes in relevant cancer samples. In addition, we analyzed the mutation spectra of these prognostic genes, and found that about 55% of the genes had mutation information, and the proportion of missense mutations was relatively high. Studies have shown that tumor genomic characteristics, mutation load, and tumor-specific neoantigens are key factors in determining a patient’s response to immune checkpoint blockers, and they may affect the patient’s immunotherapy response. Moreover, tumor mutation load and its associated tumor-specific neoantigens appear to be key ways to predict the potential clinical efficacy of immune checkpoint blockers [81].

The present study identified 23 pivotal hub genes by constructing protein-protein interaction networks and implementing the MCODE algorithm, thus shedding light on the molecular mechanisms underlying the interplay between HCMV infection and host cells. These hub genes were implicated principally in cell chemotaxis and synaptic function modulation, intimating that viruses might harness such processes to facilitate dissemination and proliferation. Meanwhile, promotion of CaM family genes (CALM1, CALM2, CALM3, CALML3, CALML4, CALML5, CALML6) and inhibition of AC family genes (ADCY1, CDCY2, ADCY3, ADCY4, ADCCY5, ADCY6, ADCY7, ADCY8, ADCY9) were observed. These are prognosis-associated genes. Notably, while mutations prevail in AC family genes expressed in manifold cancers (e.g. HNSC, KICH, KIRC, KIRP, LUAD, PRAD), the mutation rates of CaM family genes were mostly 0% across certain expressed cancers (BRCA, LGG, LIHC, PRAD, SKCM, THCA). Additionally, we utilized the GDSC database to screen for drug sensitivities, which provides a comprehensive overview of the correlations between gene expression levels and various drug responses. To further validate these findings, we employed the Connectivity Map (CMap) database, a broad bioinformatics resource used to elucidate the connections between small molecules, biological processes, and disease states by comparing gene expression profiles to predict drug mechanisms, annotate genetic variations, and provide insights for clinical trials [82–84]. Interestingly, the results between CMap and GDSC were similar, and more importantly, CMap identified the targets of drug actions. By leveraging CMap, we were able to cross-validate our preliminary findings and gain deeper insights into drug-gene interactions, thus proffering novel theoretical foundations for therapeutic and preventive interventions against the diseases. However, inter-gene interactions are intricate and multifaceted. Whether the pathway hub genes discovered in this study are the major factors influencing HCMV-induced disease progression remains to be further validated. Their purported capacities to exert pivotal roles in diverse HCMV-associated cancers also remain nebulous. Despite unveiling latent molecular mechanisms, inherent limitations exist as a purely in bioinformatical predictive study. Subsequent validation through experimental techniques is imperative to verify the expressional and functional alterations of these genes, alongside their precise roles during HCMV infection. Furthermore, the regulatory mechanisms of the hub genes and their downstream pathways, as well as the influences on viral infection and pathogenic mechanisms of diseases, warrant further in-depth research. Investigations on their consistencies of actions across the gamut of HCMV-induced cancers are necessitated, alongside assessments of their potentials as novel biomarkers or therapeutic targets for diseases. In summary, this study proffered valuable insights into HCMV and host interplay, and invoked multiple scientific issues for scholars across pertinent domains to pursue. We hope that future studies can further uncover the mystery of HCMV mechanism and provide theoretical basis for prevention and treatment of related diseases.

## Conclusions

Through a comprehensive and comprehensive analysis of the relationship between HCMV and pan-cancer, HCMV is significantly associated with many cancers and is involved in complex immune processes. We finally obtained prognostic genes and their mutations for 25 cancers, providing potential targets for clinical treatment. It is important to find the key genes of HCMV pathway species, which are closely related to cell chemotaxis and synaptic function, and in most tumors, CaM family genes are up-regulated and AC family genes are down-regulated, which may play an important role in the occurrence and development of cancer, and deserve further study.

### Electronic supplementary material

Below is the link to the electronic supplementary material.

Supplementary Material 1

Supplementary Material 2

Supplementary Material 3

Supplementary Material 4

Supplementary Material 5

Supplementary Material 6

Supplementary Material 7

Supplementary Material 8

Supplementary Material 9

## Data Availability

No datasets were generated or analysed during the current study.The data that support the findings of this study are available from the UCSC Xena database (https://xena.ucsc.edu/), and The Cancer Genome Atlas database (https://portal.gdc.cancer.gov/).

## Abbreviations

HCMV: Human cytomegalovirus
TCGA: The Cancer Genome Atlas
GTEx: The Genotype-Tissue Expression
ACC: Adrenocortical carcinoma
BLCA: Bladder Urothelial Carcinoma
BRCA: Breast invasive carcinoma
CESC: Cervical squamous cell carcinoma and endocervical adenocarcinoma
CHOL: Cholangiocarcinoma
COAD: Colon adenocarcinoma
DLBC: Lymphoid Neoplasm Diffuse Large B-cell Lymphoma
ESCA: Esophageal carcinoma
GBM: Glioblastoma multiforme
HNSC: Head and Neck squamous cell carcinoma
KICH: Kidney Chromophobe
KIRC: Kidney renal clear cell carcinoma
KIRP: Kidney renal papillary cell carcinoma
LAML: Acute Myeloid Leukemia
LGG: Brain Lower Grade Glioma
LIHC: Liver hepatocellular carcinoma
LUAD: Lung adenocarcinoma
LUSC: Lung squamous cell carcinoma
MESO: Mesothelioma
OV: Ovarian serous cystadenocarcinoma
PAAD: Pancreatic adenocarcinoma
PCPG: Pheochromocytoma and Paraganglioma
PRAD: Prostate adenocarcinoma
READ: Rectum adenocarcinoma
SARC: Sarcoma
SKCM: Skin Cutaneous Melanoma
STAD: Stomach adenocarcinoma
TGCT: Testicular Germ Cell Tumors
THCA: Thyroid carcinoma
THYM: Thymoma
UCEC: Uterine Corpus Endometrial Carcinoma
UCS: Uterine Carcinosarcoma
UVM: Uveal Melanoma
CMap: Connectivity Map
HR: Hazard ratio
CCLE: The Cancer Cell Line Encyclopedia
SVR: Support Vector Regression
ROC: receiver operating characteristic
TMB: Tumor Mutation Burden
GO: Gene Ontology
CC: Cellular Component
MF: Molecular Function
GDSC: The Genomics of Drug Sensitivity in Cancer
TIME: The tumor immune microenvironment
