# Supplementary material for: Comprehensive bioinformatics analysis of human cytomegalovirus pathway genes in pan-cancer
Source: Hum Genomics. 2024 Jun 17;18:65. doi: 10.1186/s40246-024-00633-5 (PMC11181644; doi:10.1186/s40246-024-00633-5)
Supplement: Supplementary file 3 — Supplementary Material 3 [file 40246_2024_633_MOESM3_ESM.pdf]

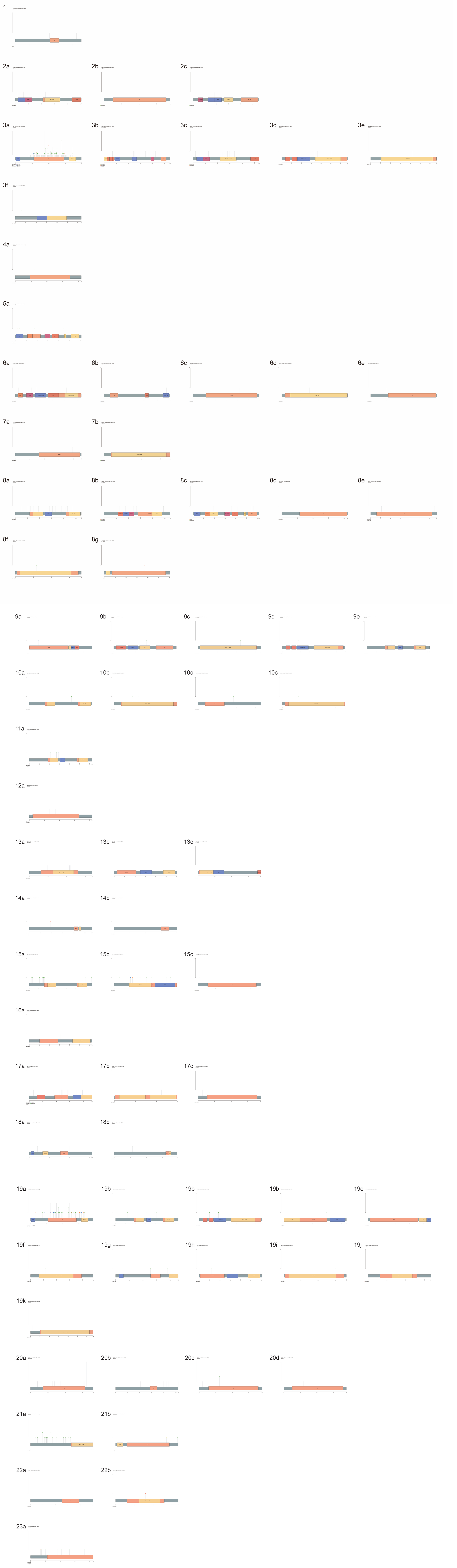

Gene mutation information of (1) ACC, (2) BLCA, (3) BRCA, (4) CHOL, (5) DLBC, (6) ESCA, (7) GBM, (8) HNSC, (9) KICH, (10) KIRC, (11) KIRP, (12) LAML, (13) LGG, (14) LIHC, (15) LUAD, (16) LUSC, (17) OV, (18) PAAD, (19) PRAD, (20) SKCM, (21) STAD, (22) THCA, (23) UCEC.
