# Supplementary material for: Comprehensive bioinformatics analysis of human cytomegalovirus pathway genes in pan-cancer
Source: Hum Genomics. 2024 Jun 17;18:65. doi: 10.1186/s40246-024-00633-5 (PMC11181644; doi:10.1186/s40246-024-00633-5)
Supplement: Supplementary file 6 — Supplementary Material 6 [file 40246_2024_633_MOESM6_ESM.pdf]

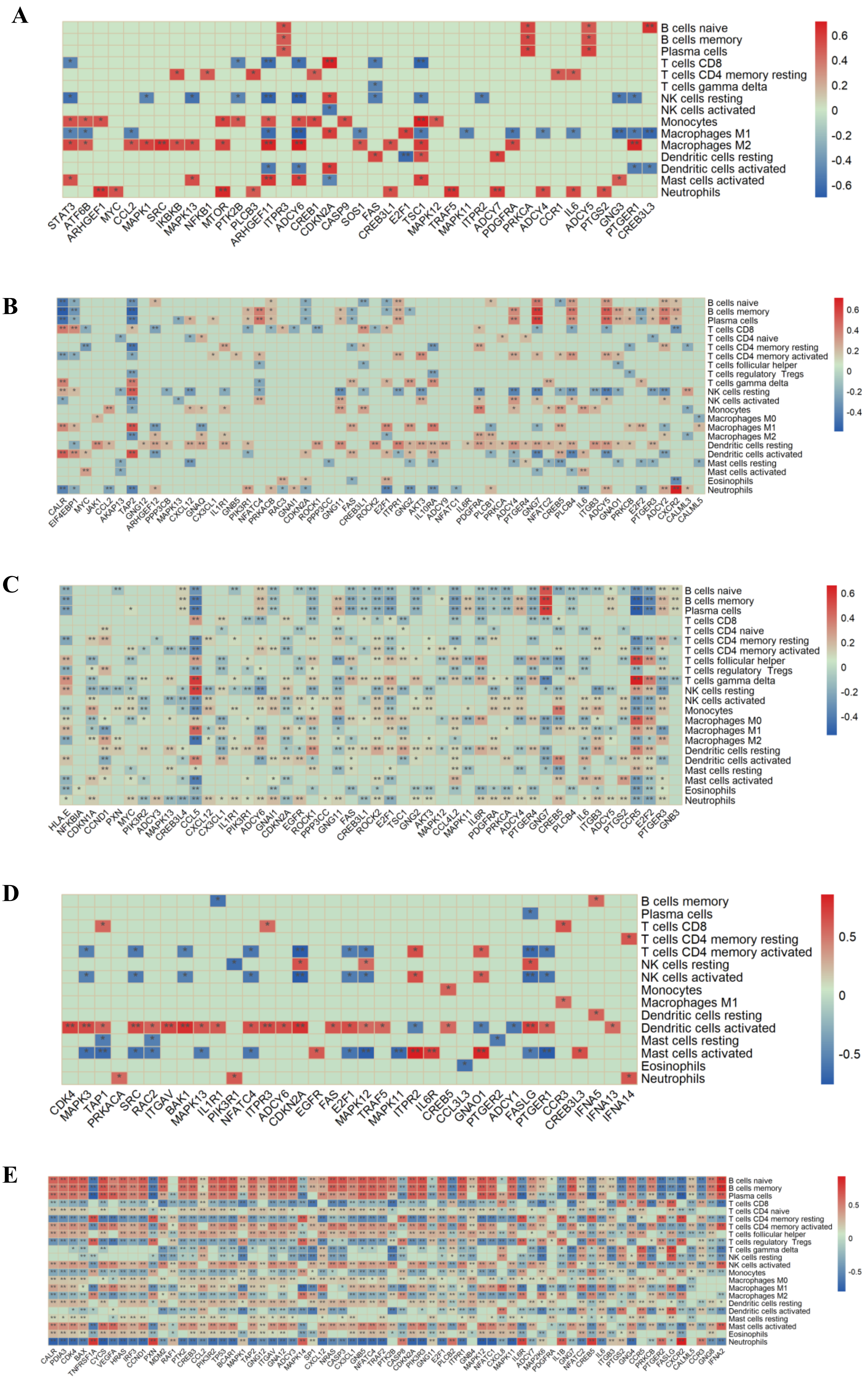
A
B cells naive
B cells memory
Plasma cells
T cells CD8
T cells CD4 memory resting
T cells gamma delta
NK cells resting
NK cells activated
Monocytes
Macrophages M1
Macrophages M2
Dendritic cells resting
Dendritic cells activated
Mast cells activated
Neutrophils
0.6
0.4
0.2
0
-0.2
-0.4
-0.6
STAT3
ATF6B
ARHGEF1
MYC
CCL2
MAPK1
SRC
IKBKB
MAPK13
NFKB1
MTOR
PTK2B
PLCB3
ARHGEF11
ITPR3
ADCY6
CREB1
CDKN2A
CASP9
SOS1
FAS
CREB3L1
E2F1
TSC1
MAPK12
TRAF5
MAPK11
ITPR2
ADCY7
PDGFRA
PRKCA
ADCY4
CCR1
IL6
ADCY5
PTGS2
GNG3
PTGER1
CREB3L3
B
B cells naive
B cells memory
Plasma cells
T cells CD8
T cells CD4 naive
T cells CD4 memory resting
T cells CD4 memory activated
T cells follicular helper
T cells regulatory Tregs
T cells gamma delta
NK cells resting
NK cells activated
Monocytes
Macrophages M0
Macrophages M1
Macrophages M2
Dendritic cells resting
Dendritic cells activated
Mast cells resting
Mast cells activated
Eosinophils
Neutrophils
0.6
0.4
0.2
0
-0.2
-0.4
C
B cells naive
B cells memory
Plasma cells
T cells CD8
T cells CD4 naive
T cells CD4 memory resting
T cells CD4 memory activated
T cells follicular helper
T cells regulatory Tregs
T cells gamma delta
NK cells resting
NK cells activated
Monocytes
Macrophages M0
Macrophages M1
Macrophages M2
Dendritic cells resting
Dendritic cells activated
Mast cells resting
Mast cells activated
Eosinophils
Neutrophils
0.6
0.4
0.2
0
-0.2
-0.4
D
B cells memory
Plasma cells
T cells CD8
T cells CD4 memory resting
T cells CD4 memory activated
NK cells resting
NK cells activated
Monocytes
Macrophages M1
Dendritic cells resting
Dendritic cells activated
Mast cells resting
Mast cells activated
Eosinophils
Neutrophils
0.5
0
-0.5
CDK4
MAPK3
TAP1
PRKACA
SRC
RAC2
ITGAV
BAK1
MAPK13
IL1R1
PIK3R1
NFATC4
ITPR3
ADCY6
CDKN2A
EGFR
FAS
E2F1
MAPK12
TRAF5
MAPK11
ITPR2
IL6R
CREB5
CCL3L3
GNAO1
PTGER2
ADCY1
FASLG
PTGER1
CCR3
CREB3L3
IFNA5
IFNA13
IFNA14
E
B cells naive
B cells memory
Plasma cells
T cells CD8
T cells CD4 naive
T cells CD4 memory resting
T cells CD4 memory activated
T cells follicular helper
T cells regulatory Tregs
T cells gamma delta
NK cells resting
NK cells activated
Monocytes
Macrophages M0
Macrophages M1
Macrophages M2
Dendritic cells resting
Dendritic cells activated
Mast cells resting
Mast cells activated
Eosinophils
Neutrophils
0.5
0
-0.5

F

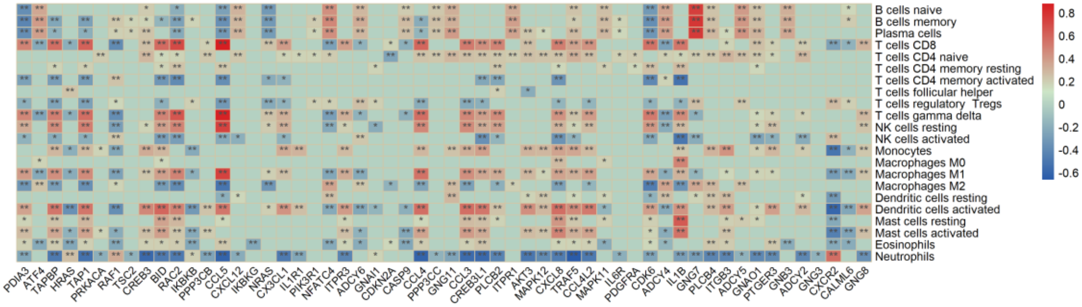
B cells naive
B cells memory
Plasma cells
T cells CD8
T cells CD4 naive
T cells CD4 memory resting
T cells CD4 memory activated
T cells follicular helper
T cells regulatory Tregs
T cells gamma delta
NK cells resting
NK cells activated
Monocytes
Macrophages M0
Macrophages M1
Macrophages M2
Dendritic cells resting
Dendritic cells activated
Mast cells resting
Mast cells activated
Eosinophils
Neutrophils
0.8
0.6
0.4
0.2
0
-0.2
-0.4
-0.6

G

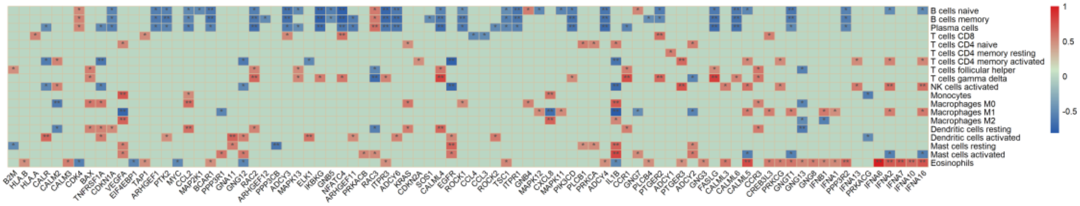

H

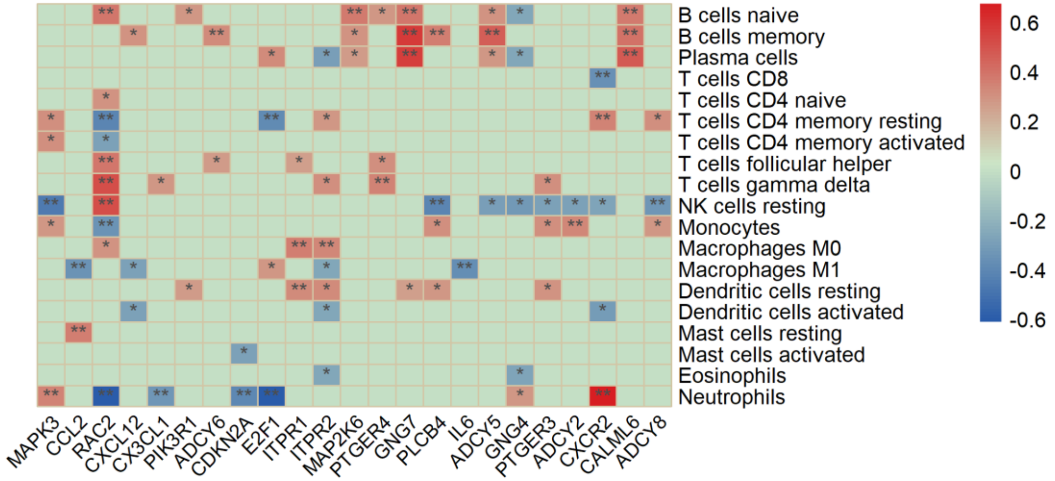
B cells naive
B cells memory
Plasma cells
T cells CD8
T cells CD4 naive
T cells CD4 memory resting
T cells CD4 memory activated
T cells follicular helper
T cells gamma delta
NK cells resting
Monocytes
Macrophages M0
Macrophages M1
Dendritic cells resting
Dendritic cells activated
Mast cells resting
Mast cells activated
Eosinophils
Neutrophils
MAPK3
CCL2
RAC2
CXCL12
CX3CL1
PIK3R1
ADCY6
CDKN2A
E2F1
ITPR1
ITPR2
MAP2K6
PTGER4
GNG7
PLCB4
IL6
ADCY5
GNG4
PTGER3
ADCY2
CXCR2
CALML6
ADCY8
0.6
0.4
0.2
0
-0.2
-0.4
-0.6

I

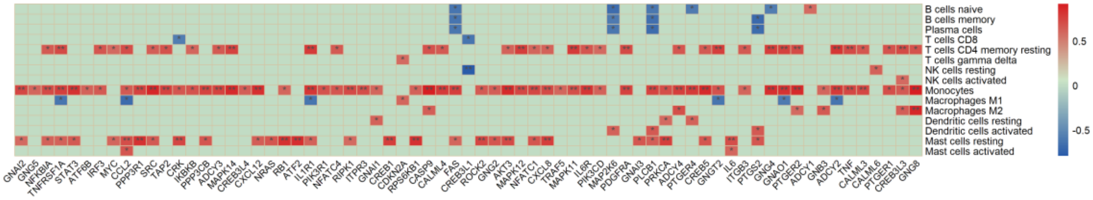

J

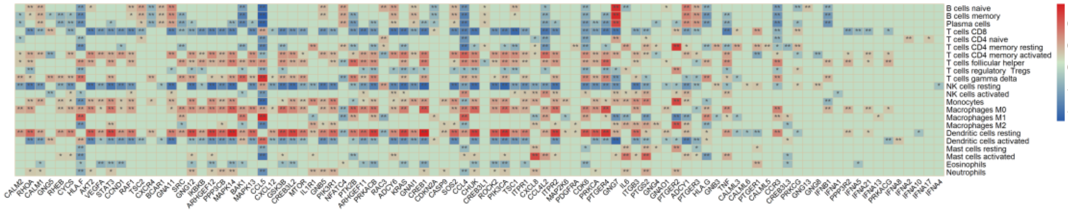

K

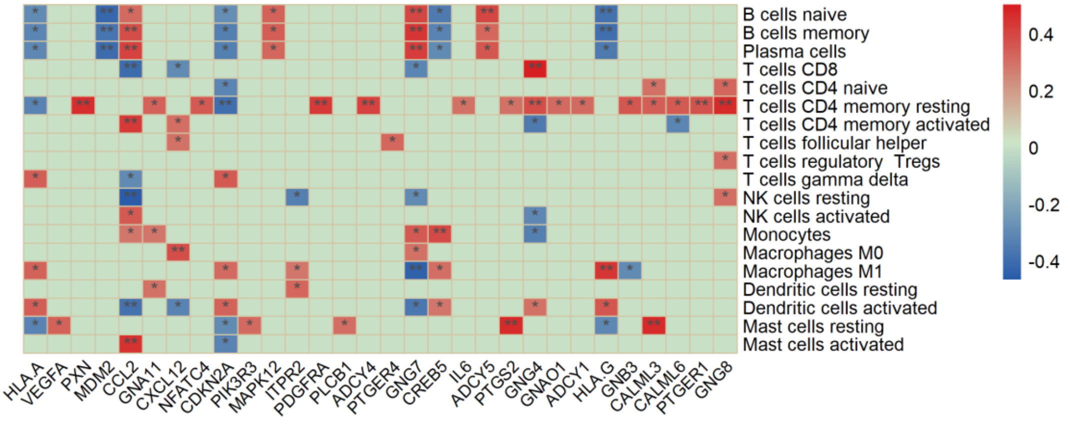
B cells naive
B cells memory
Plasma cells
T cells CD8
T cells CD4 naive
T cells CD4 memory resting
T cells CD4 memory activated
T cells follicular helper
T cells regulatory Tregs
T cells gamma delta
NK cells resting
NK cells activated
Monocytes
Macrophages M0
Macrophages M1
Dendritic cells resting
Dendritic cells activated
Mast cells resting
Mast cells activated
HLA.A
VEGFA
PXN
MDM2
CCL2
GNA11
CXCL12
NFATC4
CDKN2A
PIK3R3
MAPK12
ITPR2
PDGFRA
PLCB1
ADCY4
PTGER4
GNG7
CREB5
IL6
ADCY5
PTGS2
GNG4
GNAO1
ADCY1
HLA.G
GNB3
CALML3
CALML6
PTGER1
GNG8
0.4
0.2
0
-0.2
-0.4

L

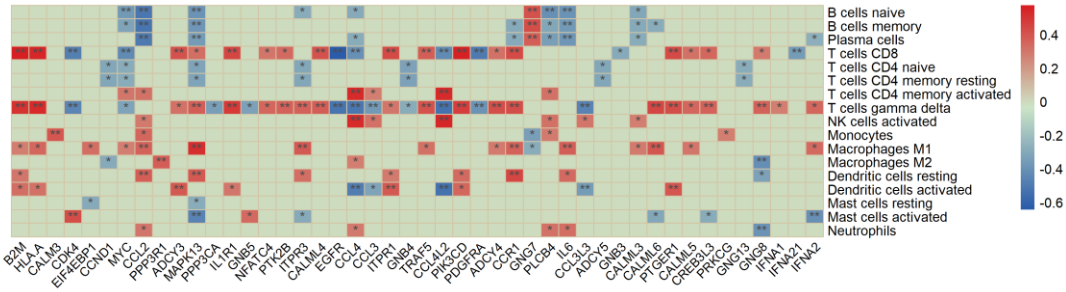
B cells naive
B cells memory
Plasma cells
T cells CD8
T cells CD4 naive
T cells CD4 memory resting
T cells CD4 memory activated
T cells gamma delta
NK cells activated
Monocytes
Macrophages M1
Macrophages M2
Dendritic cells resting
Dendritic cells activated
Mast cells resting
Mast cells activated
Neutrophils
B2M
HLA.A
CALM3
CDK4
EIF4EBP1
CCND1
MYC
CCL2
PPP3R1
ADCY3
MAPK13
PPP3CA
IL1R1
GNB5
NFATC4
PTK2B
ITPR3
CALML4
EGFR
CCL4
CCL3
ITPR1
GNB4
TRAF5
CCL4L2
PIK3CD
PDGFRA
ADCY4
CCR1
GNG7
PLCB4
IL6
CCL3L3
ADCY5
GNB3
CALML3
CALML6
PTGER1
CALML5
CREB3L3
PRKCG
GNG13
GNG8
IFNA1
IFNA21
IFNA2
0.4
0.2
0
-0.2
-0.4
-0.6

M

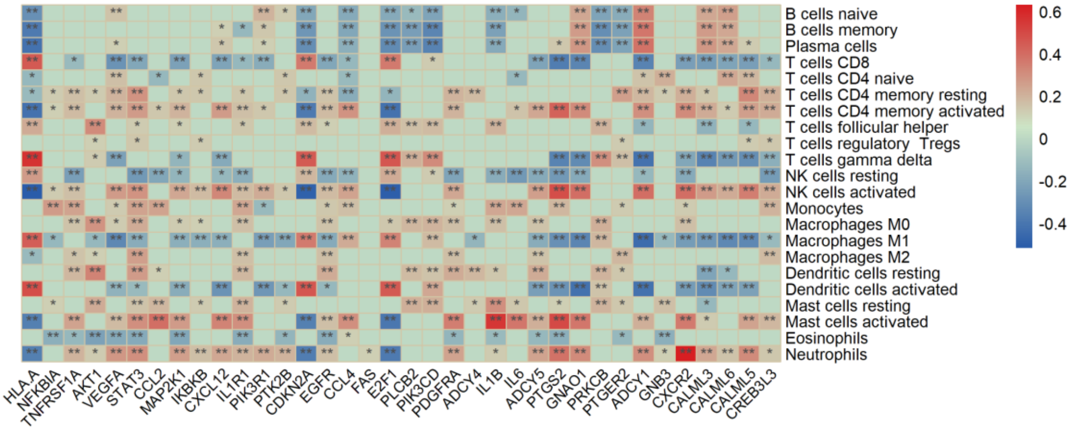
B cells naive
B cells memory
Plasma cells
T cells CD8
T cells CD4 naive
T cells CD4 memory resting
T cells CD4 memory activated
T cells follicular helper
T cells regulatory Tregs
T cells gamma delta
NK cells resting
NK cells activated
Monocytes
Macrophages M0
Macrophages M1
Macrophages M2
Dendritic cells resting
Dendritic cells activated
Mast cells resting
Mast cells activated
Eosinophils
Neutrophils
HLA.A
NFKBIA
TNFRSF1A
AKT1
VEGFA
STAT3
CCL2
MAP2K1
IKBKB
CXCL12
IL1R1
PIK3R1
PTK2B
CDKN2A
EGFR
CCL4
FAS
E2F1
PLCB2
PIK3CD
PDGFRA
ADCY4
IL1B
IL6
ADCY5
PTGS2
GNAO1
PRKCB
PTGER2
ADCY1
GNB3
CXCR2
CALML3
CALML6
CALML5
CREB3L3
0.6
0.4
0.2
0
-0.2
-0.4

N

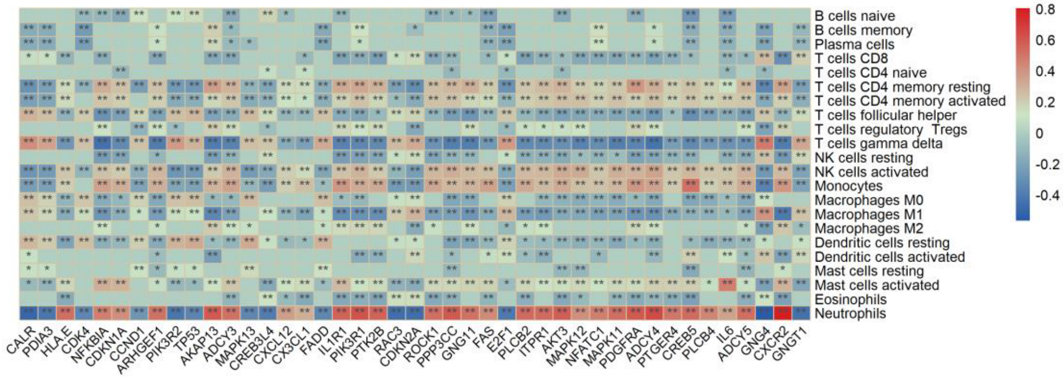
B cells naive
B cells memory
Plasma cells
T cells CD8
T cells CD4 naive
T cells CD4 memory resting
T cells CD4 memory activated
T cells follicular helper
T cells regulatory Tregs
T cells gamma delta
NK cells resting
NK cells activated
Monocytes
Macrophages M0
Macrophages M1
Macrophages M2
Dendritic cells resting
Dendritic cells activated
Mast cells resting
Mast cells activated
Eosinophils
Neutrophils
CALR
PDIA3
HLA.E
CDK4
NFKBIA
CDKN1A
CCND1
ARHGEF1
PIK3R2
TP53
AKAP13
ADCY3
MAPK13
CREB3L4
CXCL12
CX3CL1
FADD
IL1R1
PIK3R1
PTK2B
RAC3
CDKN2A
ROCK1
PPP3CC
GNG11
FAS
E2F1
PLCB2
ITPR1
AKT3
MAPK12
NFATC1
MAPK11
PDGFRA
ADCY4
PTGER4
CREB5
PLCB4
IL6
ADCY5
GNG4
CXCR2
GNGT1
0.8
0.6
0.4
0.2
0
-0.2
-0.4

O

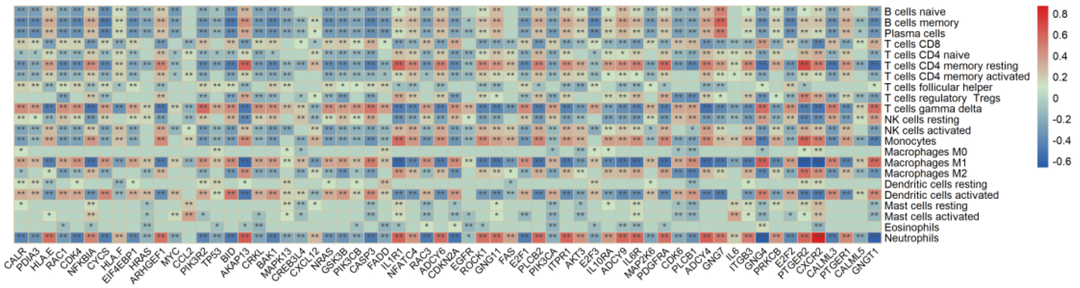

P

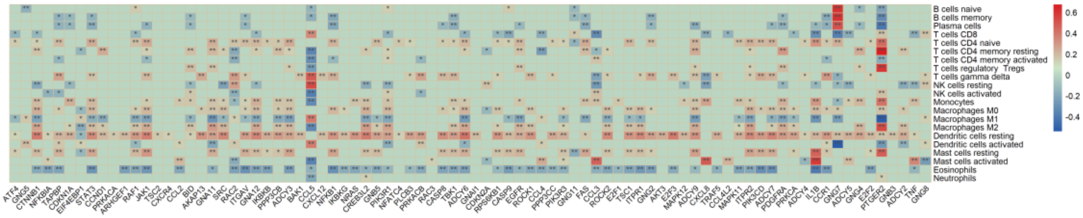

Q

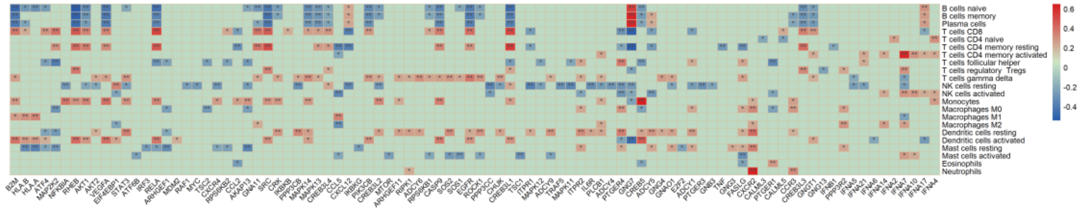

R

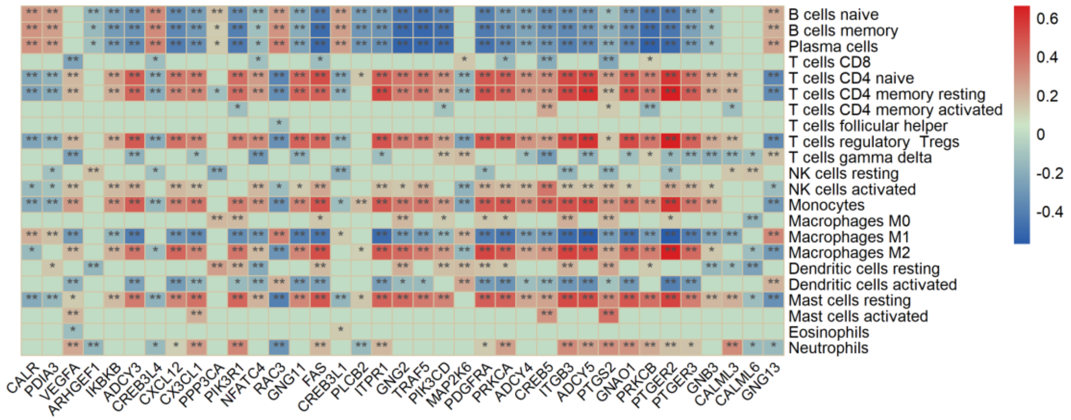
B cells naive
B cells memory
Plasma cells
T cells CD8
T cells CD4 naive
T cells CD4 memory resting
T cells CD4 memory activated
T cells follicular helper
T cells regulatory Tregs
T cells gamma delta
NK cells resting
NK cells activated
Monocytes
Macrophages M0
Macrophages M1
Macrophages M2
Dendritic cells resting
Dendritic cells activated
Mast cells resting
Mast cells activated
Eosinophils
Neutrophils
CALR
PDIA3
VEGFA
ARHGEF1
IKBKB
ADCY3
CREB3L4
CXCL12
CX3CL1
PPP3CA
PIK3R1
NFATC4
RAC3
GNG11
FAS
CREB3L1
PLCB2
ITPR1
GNG2
TRAF5
PIK3CD
MAP2K6
PDGFRA
PRKCA
ADCY4
CREB5
ITGB3
ADCY5
PTGS2
GNAO1
PRKCB
PTGER2
PTGER3
GNB3
CALML3
CALML6
GNG13
0.6
0.4
0.2
0
-0.2
-0.4

S

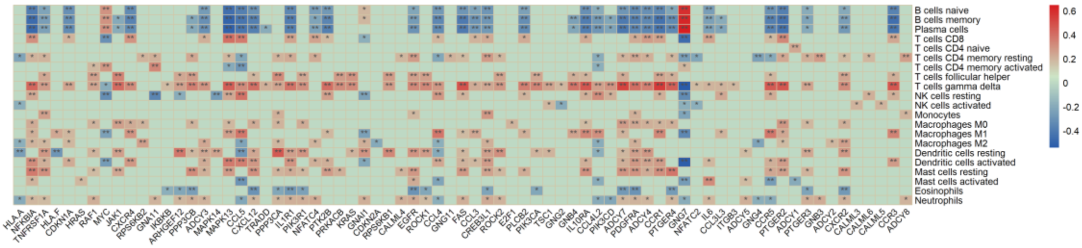

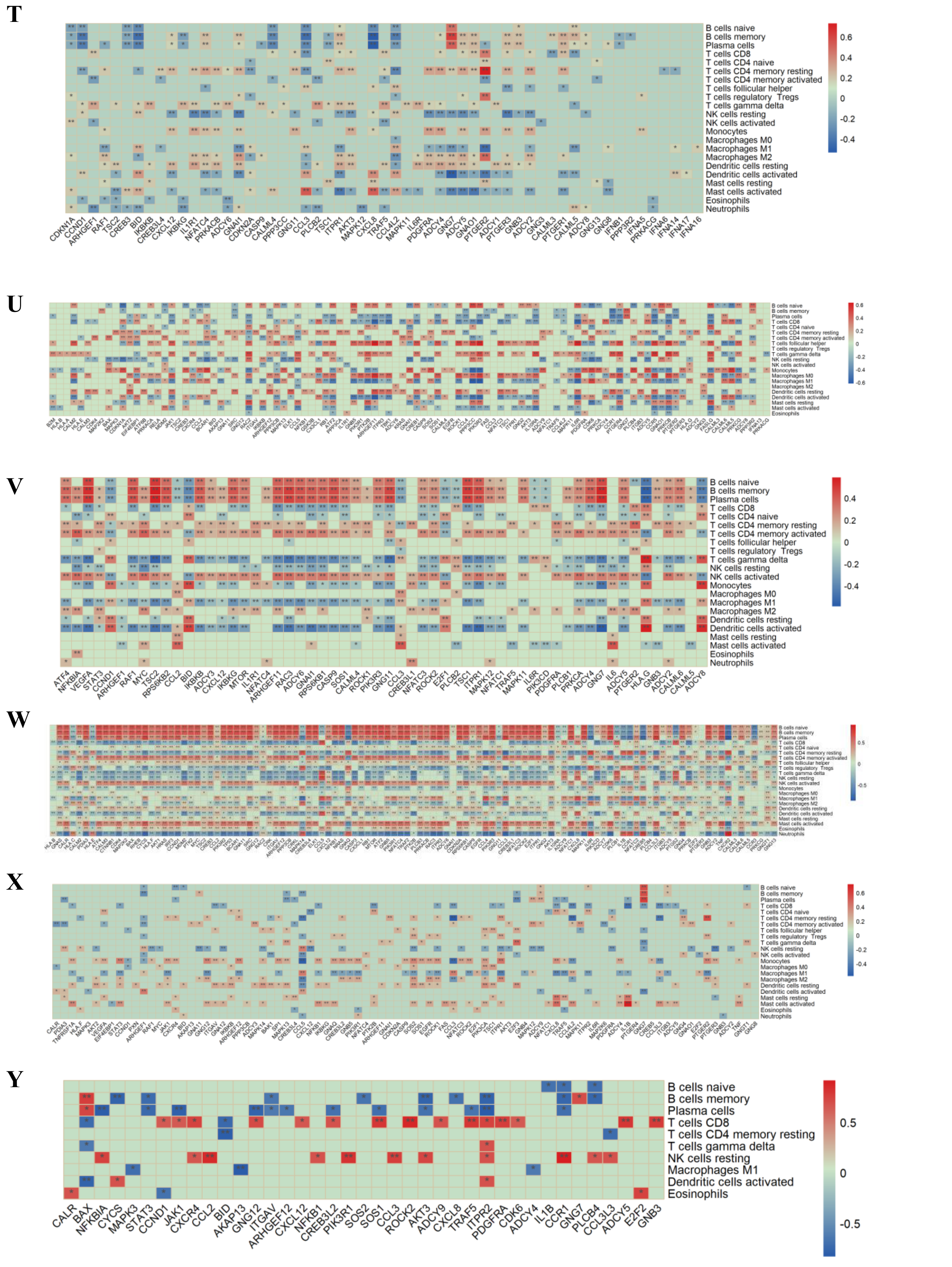

Correlation heatmaps between genes and immune cells for various cancers: (A) ACC, (B) BLCA, (C) BRCA, (D) CHOL, (E) DLBC, (F) ESCA, (G) GBM, (H) HNSC, (I) KICH, (J) KIRC, (K) KIRP, (L) LGG, (M) LIHC, (N) LUAD, (O) LUSC, (P) OV, (Q) PAAD, (R) PRAD, (S) SKCM, (T) STAD, (U) TGCT, (V) THCA, (W) THYM, (X) UCEC, and (Y) UCS. Red represents positive correlation, while blue represents negative correlation. The symbols "*", "**", "***", "****" and " " correspond to p<0.05, p<0.01, p<0.001, p<0.0001, and non-significance, respectively.
