# Supplementary material for: Comprehensive bioinformatics analysis of human cytomegalovirus pathway genes in pan-cancer
Source: Hum Genomics. 2024 Jun 17;18:65. doi: 10.1186/s40246-024-00633-5 (PMC11181644; doi:10.1186/s40246-024-00633-5)
Supplement: Supplementary file 7 — Supplementary Material 7 [file 40246_2024_633_MOESM7_ESM.pdf]

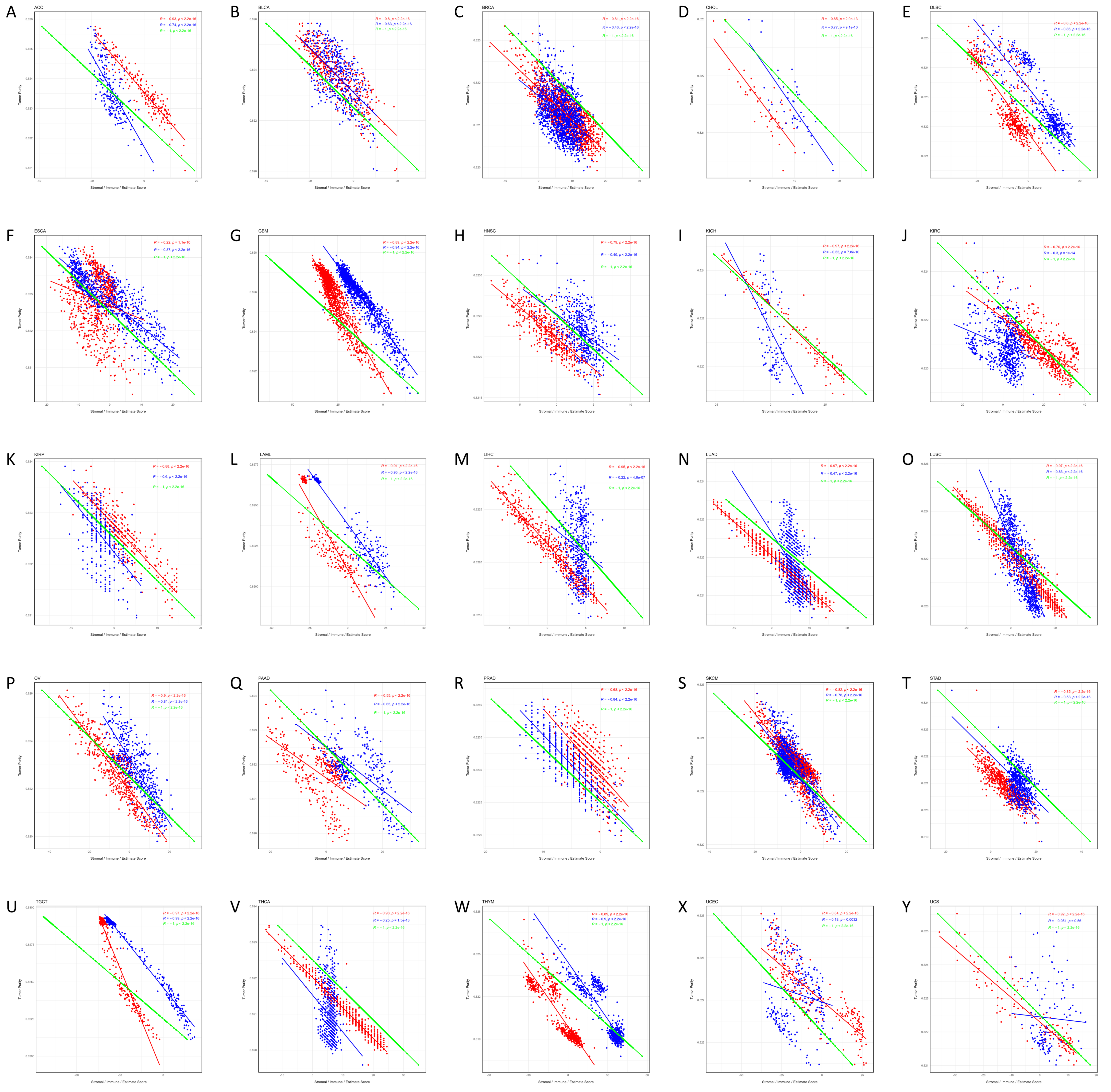

Stromal scores, immune scores and estimate scores Correlation with tumor purity of (A) ACC, (B) BLCA, (C) BRCA, (D) CHOL, (E) DLBC, (F) ESCA, (G) GBM, (H) HNSC, (I) KICH, (J) KIRC, (K) KIRP, (L) LAML, (M) LIHC, (N) LUAD, (O) LUSC, (P) OV, (Q) PAAD, (R) PRAD, (S) SKCM, (T) STAD, (U) TGCT, (V) THCA, (W) THYM, (X) UCEC, (Y)UCS. Red represents the correlation between Stromal Score and tumor purity, blue represents the correlation between Immune Score and tumor purity, green represents the correlation between ESTIMATE Score represents the correlation with tumor purity.
