# Supplementary material for: Comprehensive bioinformatics analysis of human cytomegalovirus pathway genes in pan-cancer
Source: Hum Genomics. 2024 Jun 17;18:65. doi: 10.1186/s40246-024-00633-5 (PMC11181644; doi:10.1186/s40246-024-00633-5)
Supplement: Supplementary file 8 — Supplementary Material 8 [file 40246_2024_633_MOESM8_ESM.pdf]

A

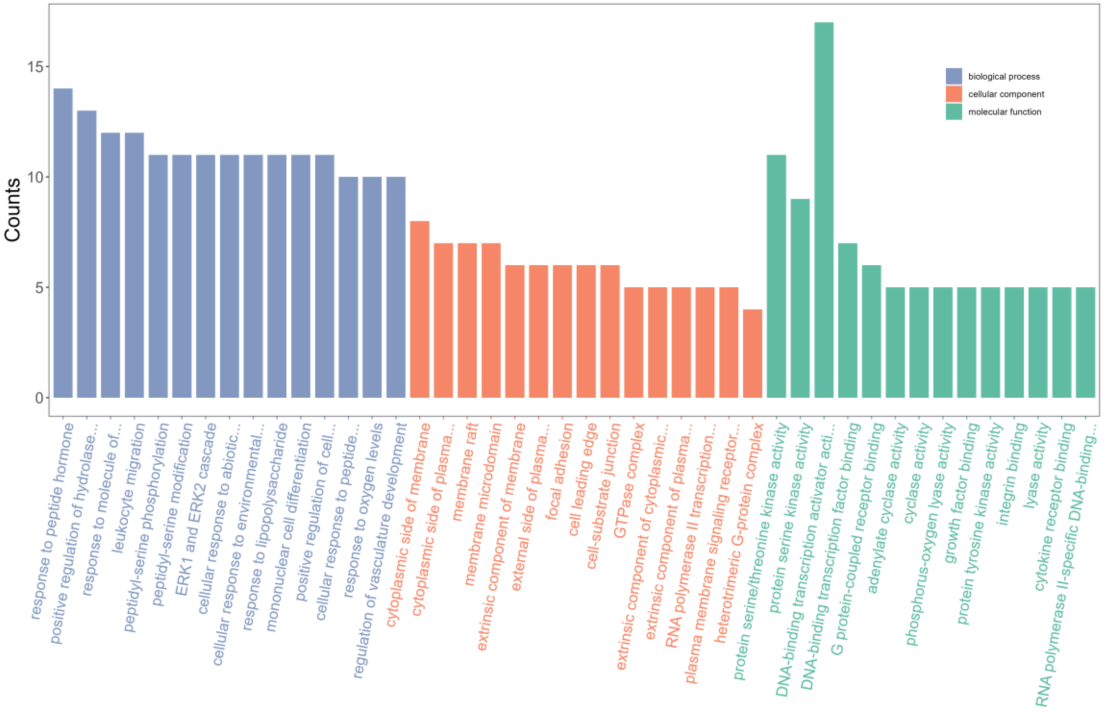
Counts
0
5
10
15
biological process
cellular component
molecular function
response to peptide hormone
positive regulation of hydrolase...
response to molecule of...
leukocyte migration
peptidyl-serine phosphorylation
peptidyl-serine modification
ERK1 and ERK2 cascade
cellular response to abiotic...
cellular response to environmental...
response to lipopolysaccharide
mononuclear cell differentiation
positive regulation of cell...
cellular response to peptide...
response to oxygen levels
regulation of vasculature development
cytoplasmic side of membrane
cytoplasmic side of plasma...
membrane raft
membrane microdomain
extrinsic component of membrane
external side of plasma...
focal adhesion
cell leading edge
cell-substrate junction
GTPase complex
extrinsic component of cytoplasmic...
extrinsic component of plasma...
RNA polymerase II transcription...
plasma membrane signaling receptor...
heterotrimeric G-protein complex
protein serine/threonine kinase activity
protein serine kinase activity
DNA-binding transcription activator acti...
DNA-binding transcription factor binding
G protein-coupled receptor binding
adenylate cyclase activity
cyclase activity
phosphorus-oxygen lyase activity
growth factor binding
protein tyrosine kinase activity
integrin binding
lyase activity
cytokine receptor binding
RNA polymerase II-specific DNA-binding...

B

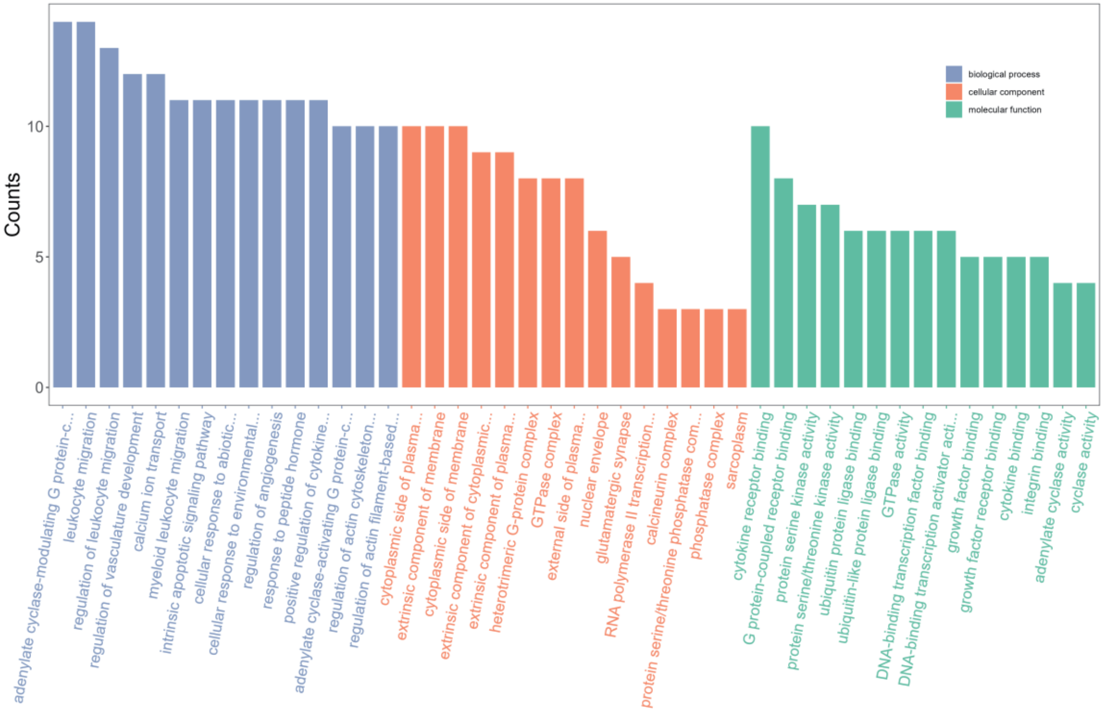
Counts
0
5
10
biological process
cellular component
molecular function
adenylate cyclase-modulating G protein-c...
leukocyte migration
regulation of leukocyte migration
regulation of vasculature development
calcium ion transport
myeloid leukocyte migration
intrinsic apoptotic signaling pathway
cellular response to abiotic...
cellular response to environmental...
regulation of angiogenesis
response to peptide hormone
positive regulation of cytokine...
adenylate cyclase-activating G protein-c...
regulation of actin cytoskeleton...
regulation of actin filament-based...
cytoplasmic side of plasma...
extrinsic component of membrane
cytoplasmic side of membrane
extrinsic component of cytoplasmic...
extrinsic component of plasma...
heterotrimeric G-protein complex
GTPase complex
external side of plasma...
nuclear envelope
glutamatergic synapse
RNA polymerase II transcription...
calcineurin complex
protein serine/threonine phosphatase com...
phosphatase complex
sarcoplasm
cytokine receptor binding
G protein-coupled receptor binding
protein serine kinase activity
protein serine/threonine kinase activity
ubiquitin protein ligase binding
ubiquitin-like protein ligase binding
GTPase activity
DNA-binding transcription factor binding
DNA-binding transcription activator acti...
growth factor binding
growth factor receptor binding
cytokine binding
integrin binding
adenylate cyclase activity
cyclase activity

C

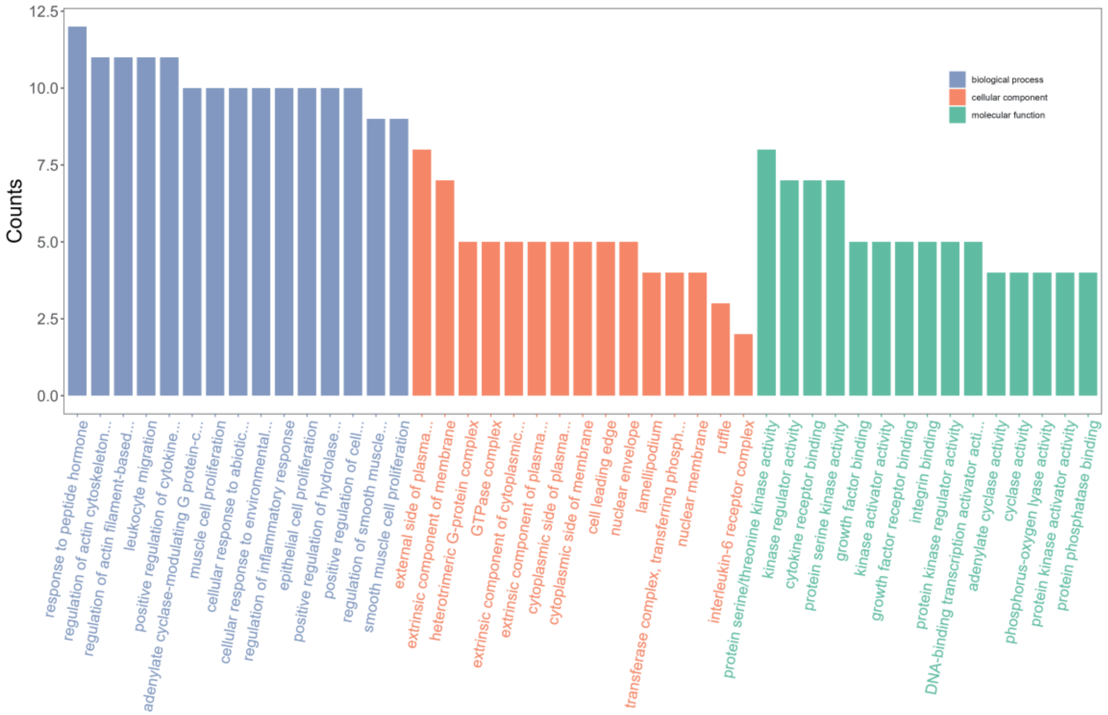

Counts
0.0
2.5
5.0
7.5
10.0
12.5
biological process
cellular component
molecular function
response to peptide hormone
regulation of actin cytoskeleton...
regulation of actin filament-based...
leukocyte migration
positive regulation of cytokine...
adenylate cyclase-modulating G protein-c...
muscle cell proliferation
cellular response to abiotic...
cellular response to environmental...
regulation of inflammatory response
epithelial cell proliferation
positive regulation of hydrolase...
positive regulation of cell...
regulation of smooth muscle...
smooth muscle cell proliferation
external side of plasma...
extrinsic component of membrane
heterotrimeric G-protein complex
GTPase complex
extrinsic component of cytoplasmic...
extrinsic component of plasma...
cytoplasmic side of plasma...
cytoplasmic side of membrane
cell leading edge
nuclear envelope
lamellipodium
transferase complex, transferring phosph...
nuclear membrane
ruffle
interleukin-6 receptor complex
protein serine/threonine kinase activity
kinase regulator activity
cytokine receptor binding
protein serine kinase activity
growth factor binding
kinase activator activity
growth factor receptor binding
integrin binding
protein kinase regulator activity
DNA-binding transcription activator acti...
adenylate cyclase activity
cyclase activity
phosphorus-oxygen lyase activity
protein kinase activator activity
protein phosphatase binding

D

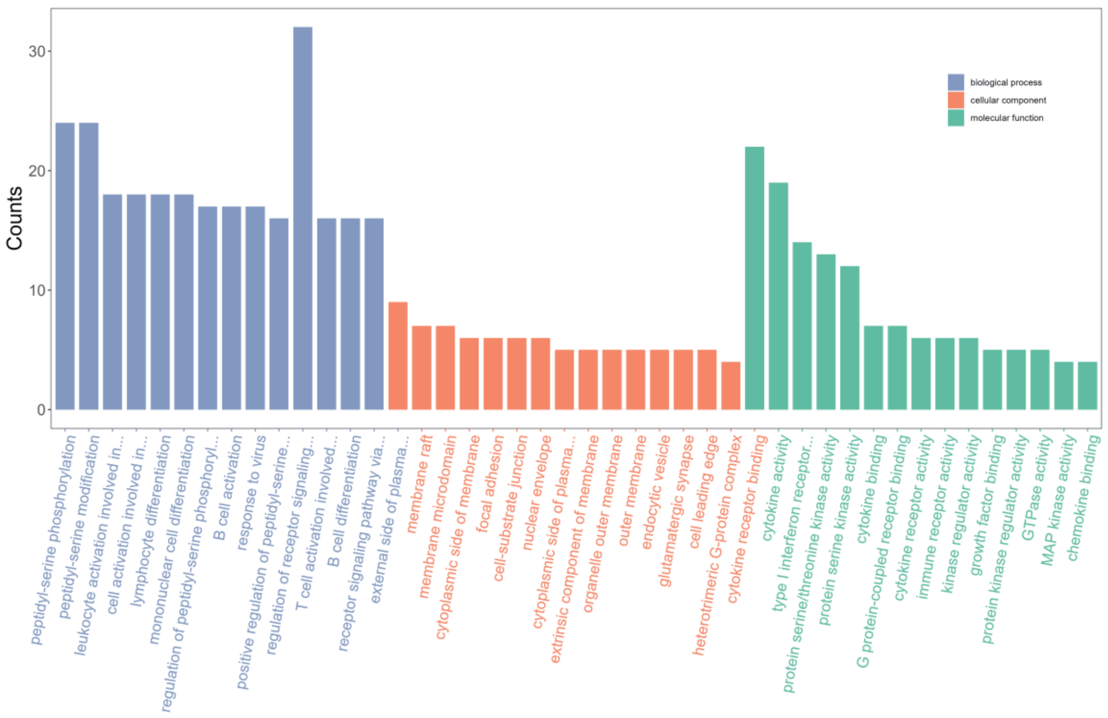

Counts
0
10
20
30
biological process
cellular component
molecular function
peptidyl-serine phosphorylation
peptidyl-serine modification
leukocyte activation involved in...
cell activation involved in...
lymphocyte differentiation
mononuclear cell differentiation
regulation of peptidyl-serine phosphoryl...
B cell activation
response to virus
positive regulation of peptidyl-serine...
regulation of receptor signaling...
T cell activation involved...
B cell differentiation
receptor signaling pathway via...
external side of plasma...
membrane raft
membrane microdomain
cytoplasmic side of membrane
focal adhesion
cell-substrate junction
nuclear envelope
cytoplasmic side of plasma...
extrinsic component of membrane
organelle outer membrane
outer membrane
endocytic vesicle
glutamatergic synapse
cell leading edge
heterotrimeric G-protein complex
cytokine receptor binding
cytokine activity
type I interferon receptor...
protein serine/threonine kinase activity
protein serine kinase activity
cytokine binding
G protein-coupled receptor binding
cytokine receptor activity
immune receptor activity
kinase regulator activity
growth factor binding
protein kinase regulator activity
GTPase activity
MAP kinase activity
chemokine binding

E

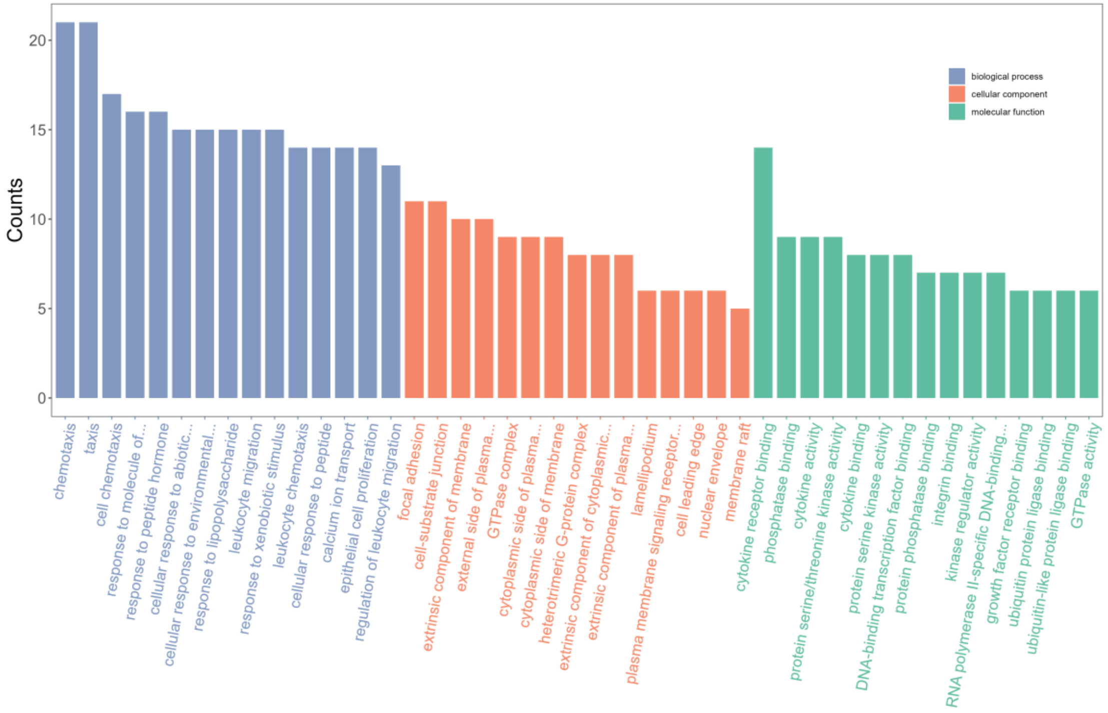
Counts
0
5
10
15
20
biological process
cellular component
molecular function
chemotaxis
taxis
cell chemotaxis
response to molecule of...
response to peptide hormone
cellular response to abiotic...
cellular response to environmental...
response to lipopolysaccharide
leukocyte migration
response to xenobiotic stimulus
leukocyte chemotaxis
cellular response to peptide
calcium ion transport
epithelial cell proliferation
regulation of leukocyte migration
focal adhesion
cell-substrate junction
extrinsic component of membrane
external side of plasma...
GTPase complex
cytoplasmic side of plasma...
cytoplasmic side of membrane
heterotrimeric G-protein complex
extrinsic component of cytoplasmic...
extrinsic component of plasma...
lamellipodium
plasma membrane signaling receptor...
cell leading edge
nuclear envelope
membrane raft
cytokine receptor binding
phosphatase binding
cytokine activity
protein serine/threonine kinase activity
cytokine binding
protein serine kinase activity
DNA-binding transcription factor binding
protein phosphatase binding
integrin binding
kinase regulator activity
RNA polymerase II-specific DNA-binding...
growth factor receptor binding
ubiquitin protein ligase binding
ubiquitin-like protein ligase binding
GTPase activity

F

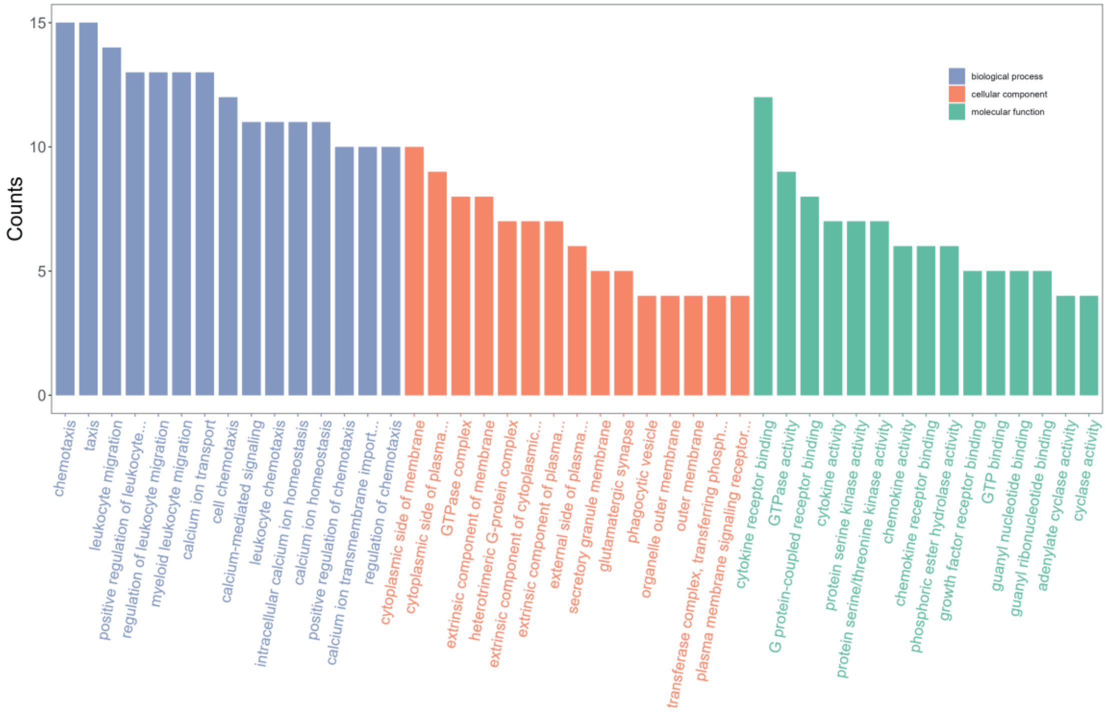
Counts
0
5
10
15
biological process
cellular component
molecular function
chemotaxis
taxis
leukocyte migration
positive regulation of leukocyte...
regulation of leukocyte migration
myeloid leukocyte migration
calcium ion transport
cell chemotaxis
calcium-mediated signaling
leukocyte chemotaxis
intracellular calcium ion homeostasis
calcium ion homeostasis
positive regulation of chemotaxis
calcium ion transmembrane import...
regulation of chemotaxis
cytoplasmic side of membrane
cytoplasmic side of plasma...
GTPase complex
extrinsic component of membrane
heterotrimeric G-protein complex
extrinsic component of cytoplasmic...
extrinsic component of plasma...
external side of plasma...
secretory granule membrane
glutamatergic synapse
phagocytic vesicle
organelle outer membrane
outer membrane
transferase complex, transferring phosph...
plasma membrane signaling receptor...
cytokine receptor binding
GTPase activity
G protein-coupled receptor binding
cytokine activity
protein serine kinase activity
protein serine/threonine kinase activity
chemokine activity
chemokine receptor binding
phosphoric ester hydrolase activity
growth factor receptor binding
GTP binding
guanyl nucleotide binding
guanyl ribonucleotide binding
adenylate cyclase activity
cyclase activity

G

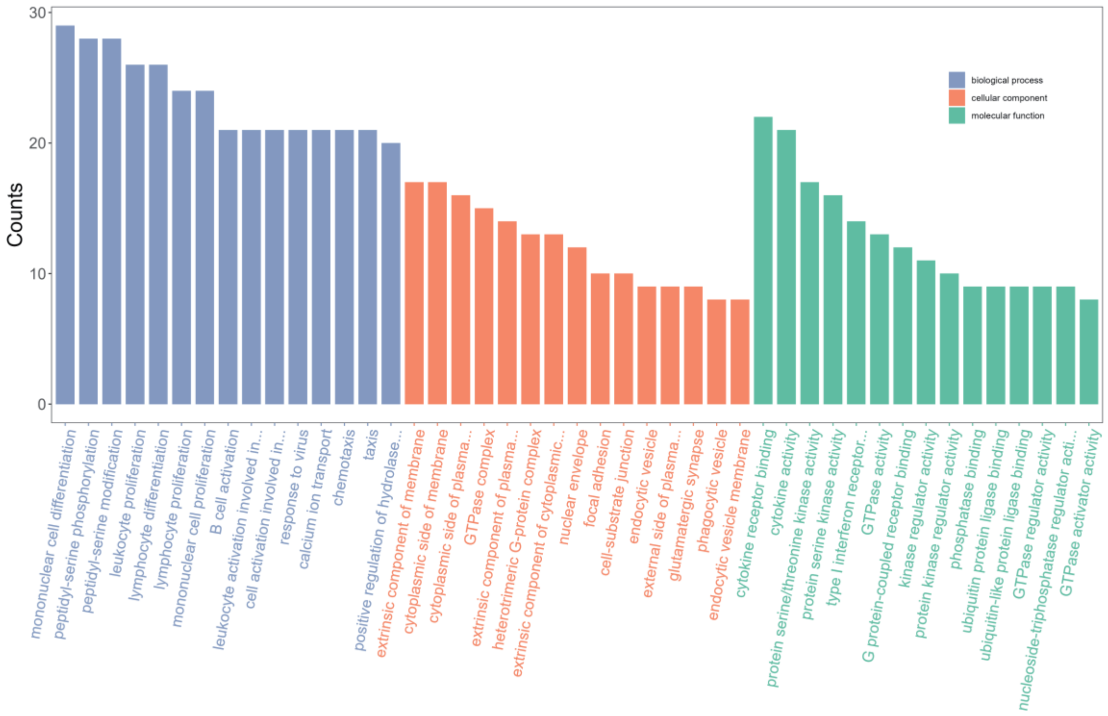

Counts
30
20
10
0
biological process
cellular component
molecular function
mononuclear cell differentiation
peptidyl-serine phosphorylation
peptidyl-serine modification
leukocyte proliferation
lymphocyte differentiation
lymphocyte proliferation
mononuclear cell proliferation
B cell activation
leukocyte activation involved in...
cell activation involved in...
response to virus
calcium ion transport
chemotaxis
taxis
positive regulation of hydrolase...
extrinsic component of membrane
cytoplasmic side of membrane
cytoplasmic side of plasma...
GTPase complex
extrinsic component of plasma...
heterotrimeric G-protein complex
extrinsic component of cytoplasmic...
nuclear envelope
focal adhesion
cell-substrate junction
endocytic vesicle
external side of plasma...
glutamatergic synapse
phagocytic vesicle
endocytic vesicle membrane
cytokine receptor binding
cytokine activity
protein serine/threonine kinase activity
protein serine kinase activity
type I interferon receptor...
GTPase activity
G protein-coupled receptor binding
kinase regulator activity
protein kinase regulator activity
phosphatase binding
ubiquitin protein ligase binding
ubiquitin-like protein ligase binding
GTPase regulator activity
nucleoside-triphosphatase regulator acti...
GTPase activator activity

H

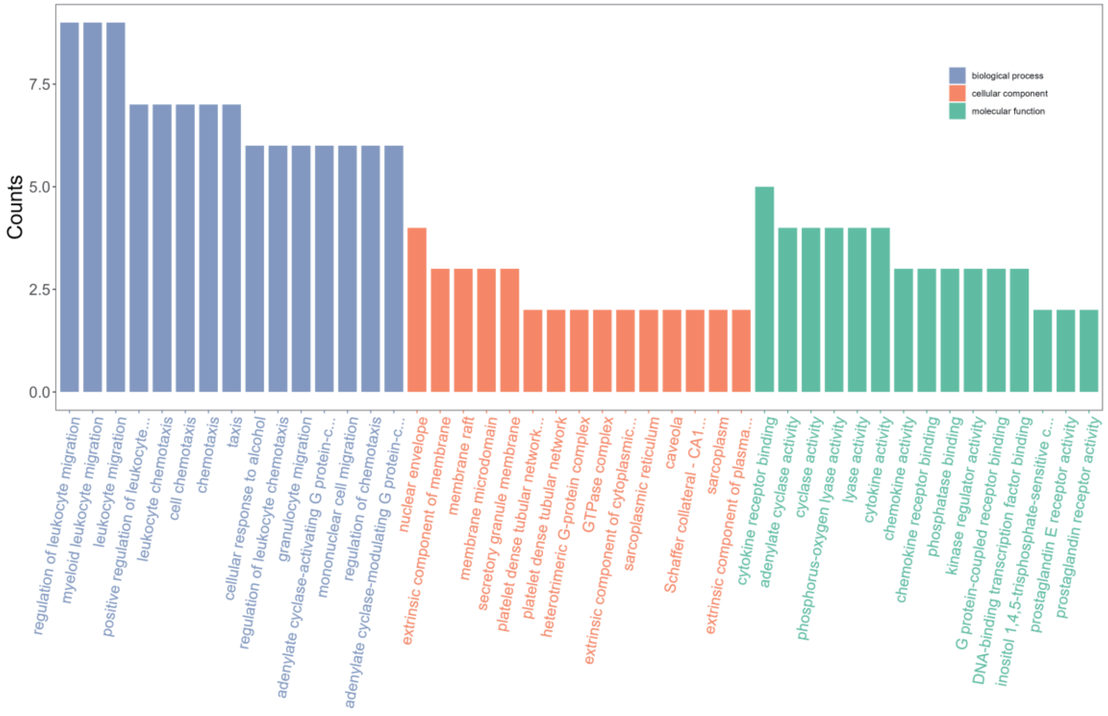

Counts
7.5
5.0
2.5
0.0
biological process
cellular component
molecular function
regulation of leukocyte migration
myeloid leukocyte migration
leukocyte migration
positive regulation of leukocyte...
leukocyte chemotaxis
cell chemotaxis
chemotaxis
taxis
cellular response to alcohol
regulation of leukocyte chemotaxis
granulocyte migration
adenylate cyclase-activating G protein-c...
mononuclear cell migration
regulation of chemotaxis
adenylate cyclase-modulating G protein-c...
nuclear envelope
extrinsic component of membrane
membrane raft
membrane microdomain
secretory granule membrane
platelet dense tubular network...
platelet dense tubular network
heterotrimeric G-protein complex
GTPase complex
extrinsic component of cytoplasmic...
sarcoplasmic reticulum
caveola
Schaffer collateral - CA1...
sarcoplasm
extrinsic component of plasma...
cytokine receptor binding
adenylate cyclase activity
cyclase activity
phosphorus-oxygen lyase activity
lyase activity
cytokine activity
chemokine activity
chemokine receptor binding
phosphatase binding
kinase regulator activity
G protein-coupled receptor binding
DNA-binding transcription factor binding
inositol 1,4,5-trisphosphate-sensitive c...
prostaglandin E receptor activity
prostaglandin receptor activity

I

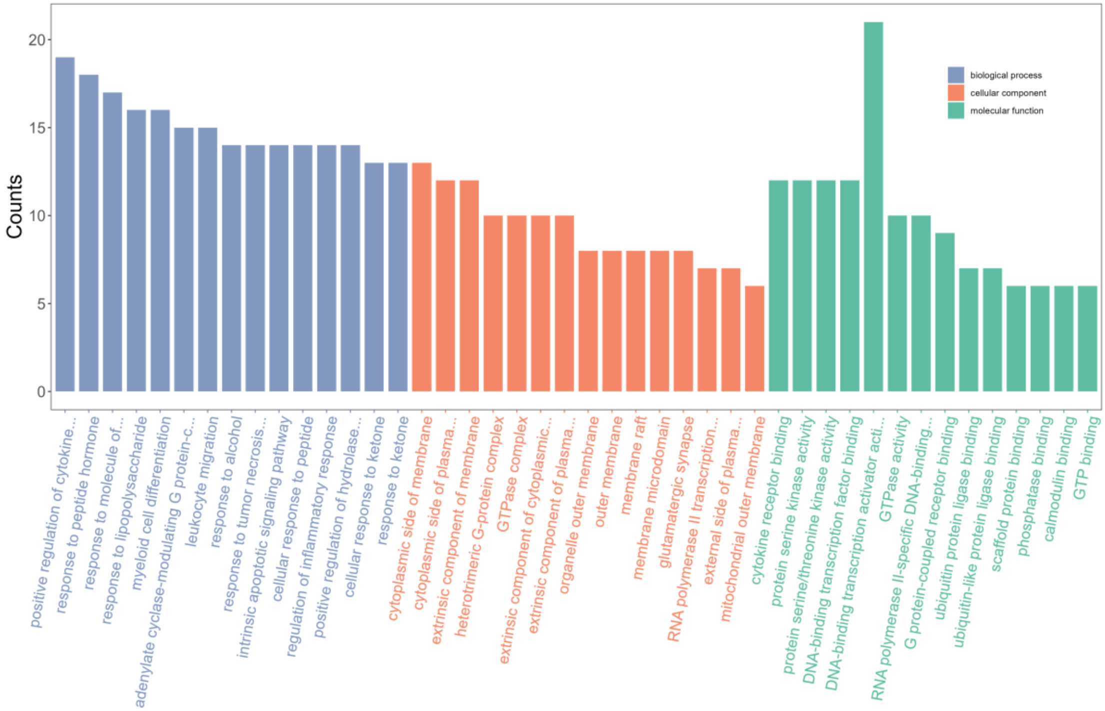
Counts
biological process
cellular component
molecular function
positive regulation of cytokine...
response to peptide hormone
response to molecule of...
response to lipopolysaccharide
myeloid cell differentiation
adenylate cyclase-modulating G protein-c...
leukocyte migration
response to alcohol
response to tumor necrosis...
intrinsic apoptotic signaling pathway
cellular response to peptide
regulation of inflammatory response
positive regulation of hydrolase...
cellular response to ketone
response to ketone
cytoplasmic side of membrane
cytoplasmic side of plasma...
extrinsic component of membrane
heterotrimeric G-protein complex
GTPase complex
extrinsic component of cytoplasmic...
extrinsic component of plasma...
organelle outer membrane
outer membrane
membrane raft
membrane microdomain
glutamatergic synapse
RNA polymerase II transcription...
external side of plasma...
mitochondrial outer membrane
cytokine receptor binding
protein serine kinase activity
protein serine/threonine kinase activity
DNA-binding transcription factor binding
DNA-binding transcription activator acti...
GTPase activity
RNA polymerase II-specific DNA-binding...
G protein-coupled receptor binding
ubiquitin protein ligase binding
ubiquitin-like protein ligase binding
scaffold protein binding
phosphatase binding
calmodulin binding
GTP binding

J

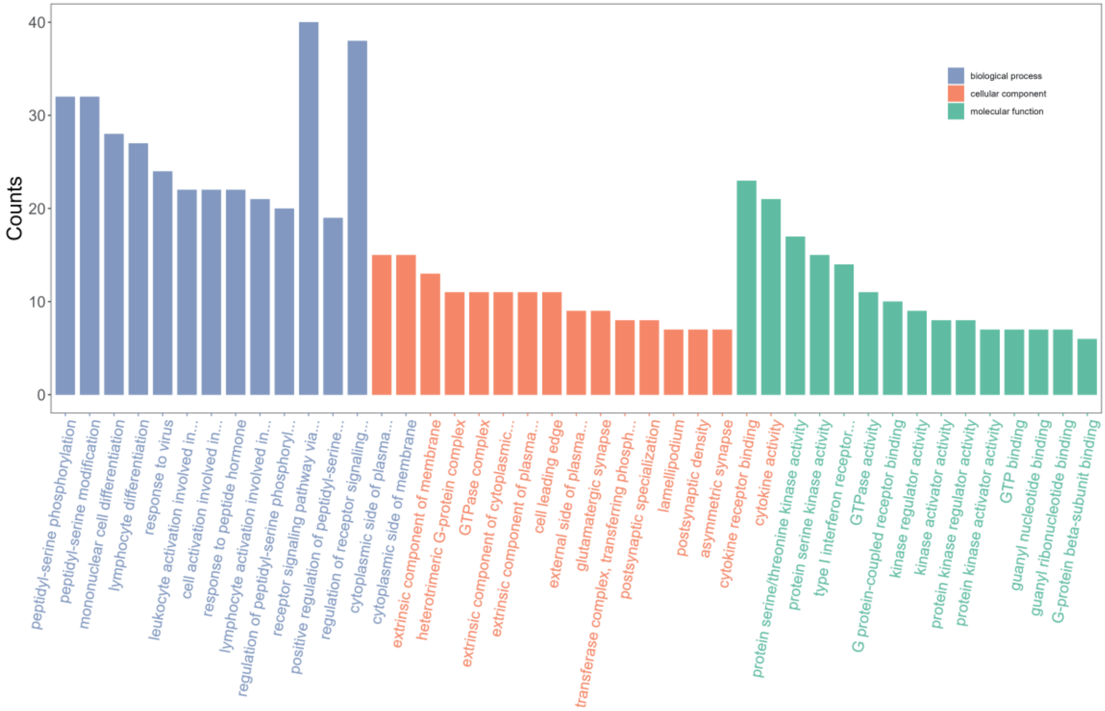
Counts
biological process
cellular component
molecular function
peptidyl-serine phosphorylation
peptidyl-serine modification
mononuclear cell differentiation
lymphocyte differentiation
response to virus
leukocyte activation involved in...
cell activation involved in...
response to peptide hormone
lymphocyte activation involved in...
regulation of peptidyl-serine phosphoryl...
receptor signaling pathway via...
positive regulation of peptidyl-serine...
regulation of receptor signaling...
cytoplasmic side of plasma...
cytoplasmic side of membrane
extrinsic component of membrane
heterotrimeric G-protein complex
GTPase complex
extrinsic component of cytoplasmic...
extrinsic component of plasma...
cell leading edge
external side of plasma...
glutamatergic synapse
transferase complex, transferring phosph...
postsynaptic specialization
lamellipodium
postsynaptic density
asymmetric synapse
cytokine receptor binding
cytokine activity
protein serine/threonine kinase activity
protein serine kinase activity
type I interferon receptor...
GTPase activity
G protein-coupled receptor binding
kinase regulator activity
kinase activator activity
protein kinase regulator activity
protein kinase activator activity
GTP binding
guanyl nucleotide binding
guanyl ribonucleotide binding
G-protein beta-subunit binding

K

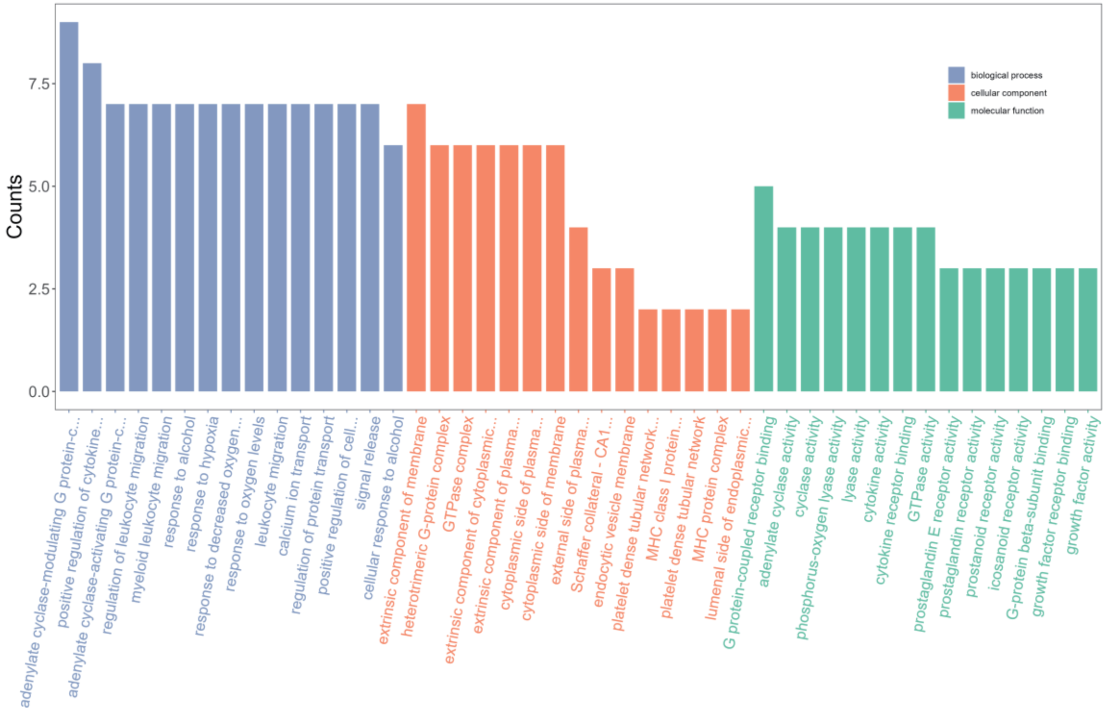
Counts
biological process
cellular component
molecular function
adenylate cyclase-modulating G protein-c...
positive regulation of cytokine...
adenylate cyclase-activating G protein-c...
regulation of leukocyte migration
myeloid leukocyte migration
response to alcohol
response to hypoxia
response to decreased oxygen...
response to oxygen levels
leukocyte migration
calcium ion transport
regulation of protein transport
positive regulation of cell...
signal release
cellular response to alcohol
extrinsic component of membrane
heterotrimeric G-protein complex
GTPase complex
extrinsic component of cytoplasmic...
extrinsic component of plasma...
cytoplasmic side of plasma...
cytoplasmic side of membrane
external side of plasma...
Schaffer collateral - CA1...
endocytic vesicle membrane
platelet dense tubular network...
MHC class I protein...
platelet dense tubular network
MHC protein complex
lumenal side of endoplasmic...
G protein-coupled receptor binding
adenylate cyclase activity
cyclase activity
phosphorus-oxygen lyase activity
lyase activity
cytokine activity
cytokine receptor binding
GTPase activity
prostaglandin E receptor activity
prostaglandin receptor activity
prostanoid receptor activity
icosanoid receptor activity
G-protein beta-subunit binding
growth factor receptor binding
growth factor activity

L

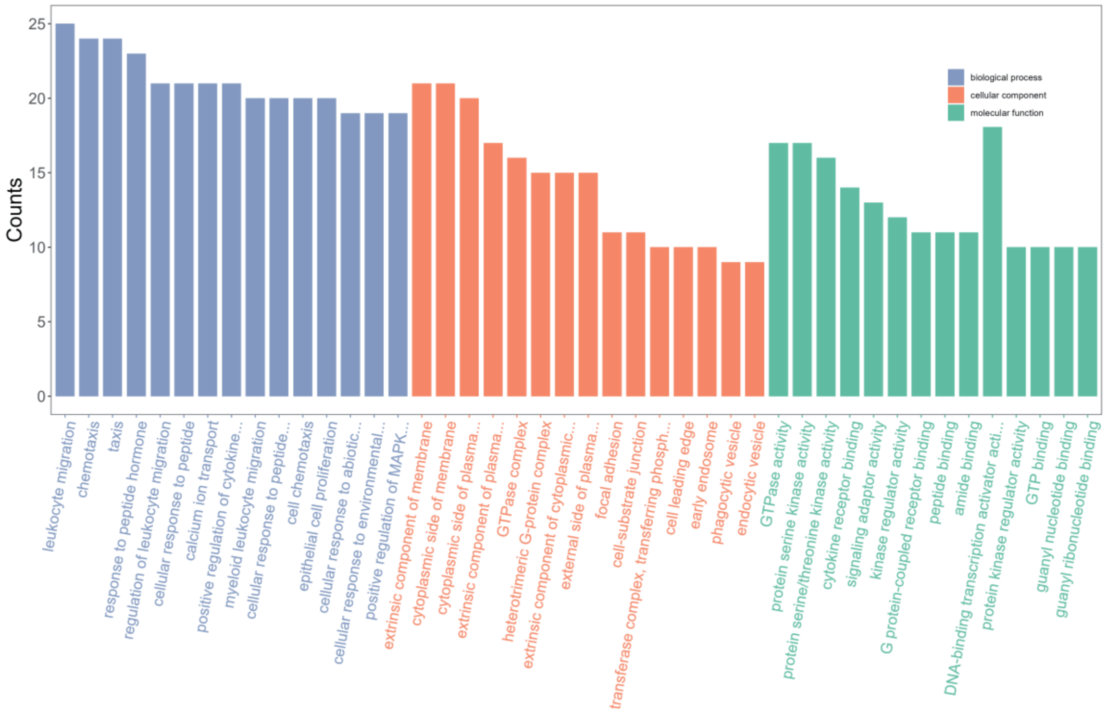
Counts
biological process
cellular component
molecular function
leukocyte migration
chemotaxis
taxis
response to peptide hormone
regulation of leukocyte migration
cellular response to peptide
calcium ion transport
positive regulation of cytokine...
myeloid leukocyte migration
cellular response to peptide...
cell chemotaxis
epithelial cell proliferation
cellular response to abiotic...
cellular response to environmental...
positive regulation of MAPK...
extrinsic component of membrane
cytoplasmic side of membrane
cytoplasmic side of plasma...
extrinsic component of plasma...
GTPase complex
heterotrimeric G-protein complex
extrinsic component of cytoplasmic...
external side of plasma...
focal adhesion
cell-substrate junction
transferase complex, transferring phosph...
cell leading edge
early endosome
phagocytic vesicle
endocytic vesicle
GTPase activity
protein serine kinase activity
protein serine/threonine kinase activity
cytokine receptor binding
signaling adaptor activity
kinase regulator activity
G protein-coupled receptor binding
peptide binding
amide binding
DNA-binding transcription activator acti...
protein kinase regulator activity
GTP binding
guanyl nucleotide binding
guanyl ribonucleotide binding

M

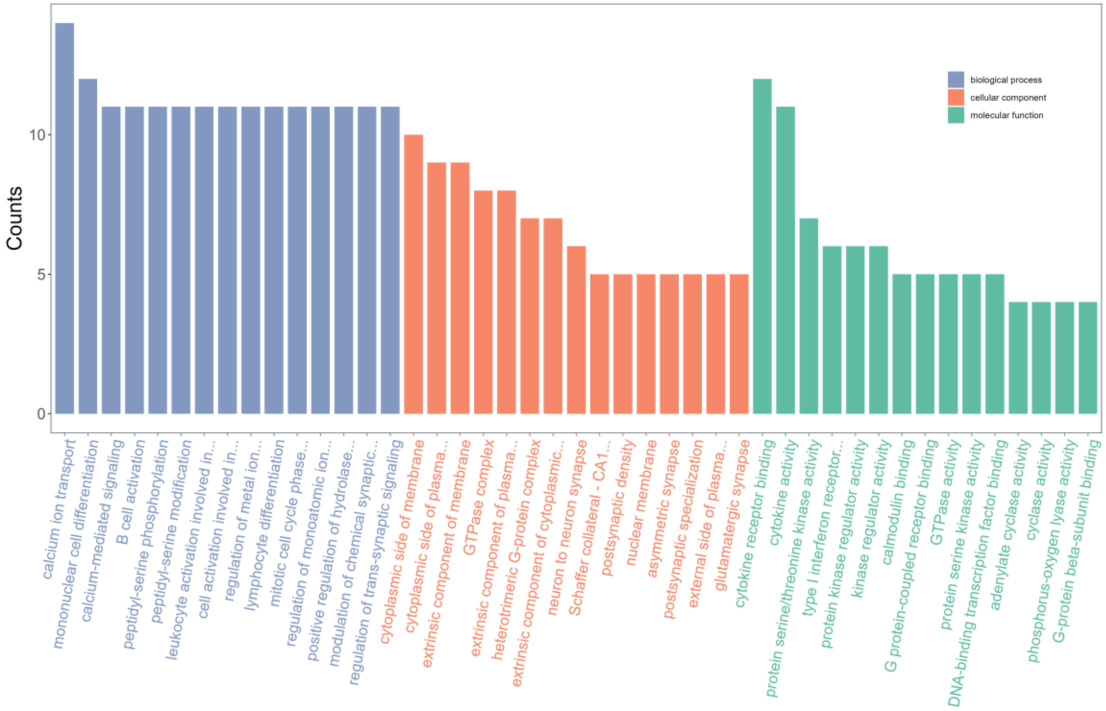

biological process
cellular component
molecular function
Counts
0
5
10
calcium ion transport
mononuclear cell differentiation
calcium-mediated signaling
B cell activation
peptidyl-serine phosphorylation
peptidyl-serine modification
leukocyte activation involved in...
cell activation involved in...
regulation of metal ion...
lymphocyte differentiation
mitotic cell cycle phase...
regulation of monoatomic ion...
positive regulation of hydrolase...
modulation of chemical synaptic...
regulation of trans-synaptic signaling
cytoplasmic side of membrane
cytoplasmic side of plasma...
extrinsic component of membrane
GTPase complex
extrinsic component of plasma...
heterotrimeric G-protein complex
extrinsic component of cytoplasmic...
neuron to neuron synapse
Schaffer collateral - CA1...
postsynaptic density
nuclear membrane
asymmetric synapse
postsynaptic specialization
external side of plasma...
glutamatergic synapse
cytokine receptor binding
cytokine activity
protein serine/threonine kinase activity
type I interferon receptor...
protein kinase regulator activity
kinase regulator activity
calmodulin binding
G protein-coupled receptor binding
GTPase activity
protein serine kinase activity
DNA-binding transcription factor binding
adenylate cyclase activity
cyclase activity
phosphorus-oxygen lyase activity
G-protein beta-subunit binding

N

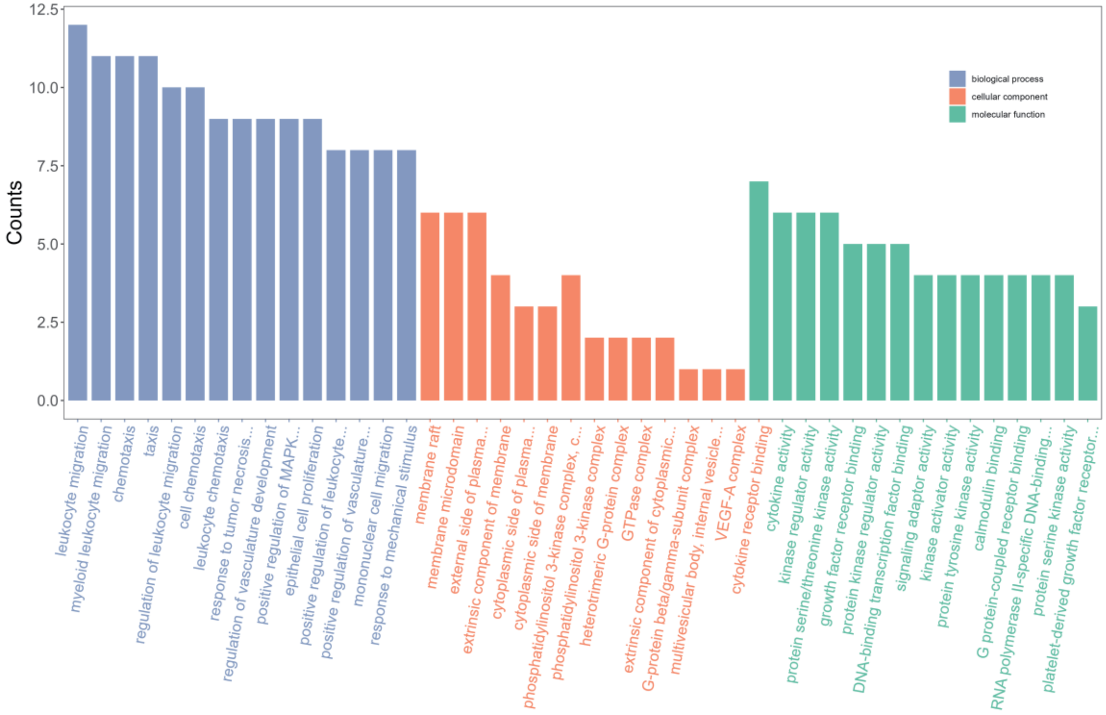

biological process
cellular component
molecular function
Counts
0.0
2.5
5.0
7.5
10.0
12.5
leukocyte migration
myeloid leukocyte migration
chemotaxis
taxis
regulation of leukocyte migration
cell chemotaxis
leukocyte chemotaxis
response to tumor necrosis...
regulation of vasculature development
positive regulation of MAPK...
epithelial cell proliferation
positive regulation of leukocyte...
positive regulation of vasculature...
mononuclear cell migration
response to mechanical stimulus
membrane raft
membrane microdomain
external side of plasma...
extrinsic component of membrane
cytoplasmic side of plasma...
cytoplasmic side of membrane
phosphatidylinositol 3-kinase complex, c...
phosphatidylinositol 3-kinase complex
heterotrimeric G-protein complex
GTPase complex
extrinsic component of cytoplasmic...
G-protein beta/gamma-subunit complex
multivesicular body, internal vesicle...
VEGF-A complex
cytokine receptor binding
cytokine activity
kinase regulator activity
protein serine/threonine kinase activity
growth factor receptor binding
protein kinase regulator activity
DNA-binding transcription factor binding
signaling adaptor activity
kinase activator activity
protein tyrosine kinase activity
calmodulin binding
G protein-coupled receptor binding
RNA polymerase II-specific DNA-binding...
protein serine kinase activity
platelet-derived growth factor receptor...

O

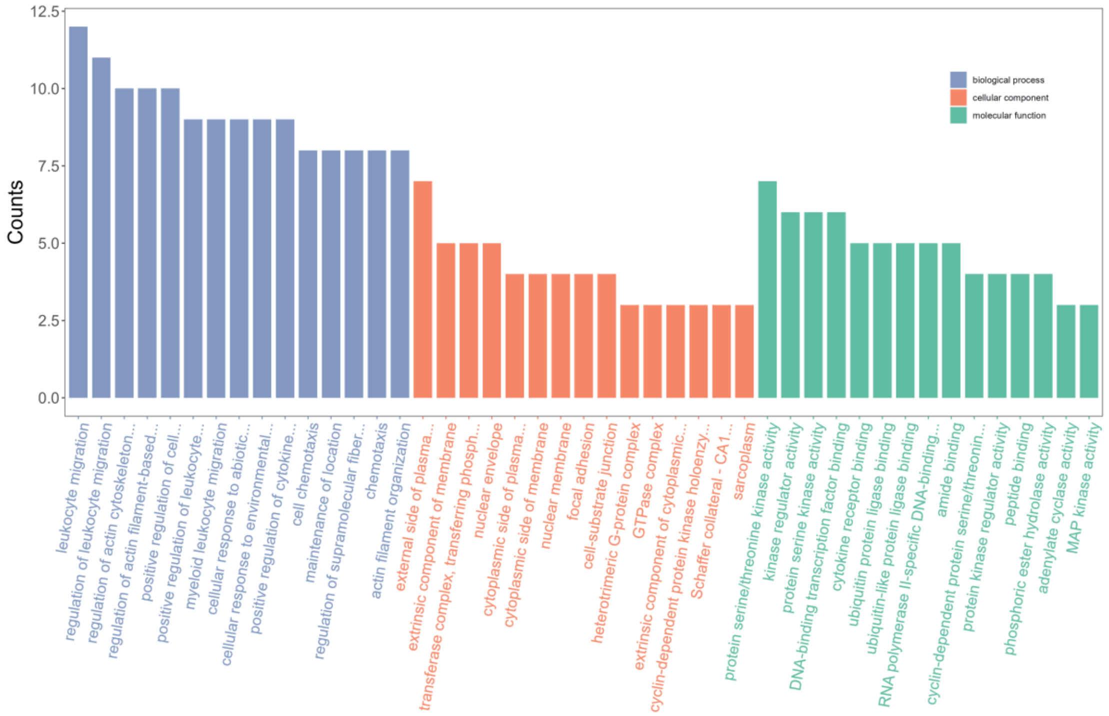

Counts
12.5
10.0
7.5
5.0
2.5
0.0
biological process
cellular component
molecular function
leukocyte migration
regulation of leukocyte migration
regulation of actin cytoskeleton...
regulation of actin filament-based...
positive regulation of cell...
positive regulation of leukocyte...
myeloid leukocyte migration
cellular response to abiotic...
cellular response to environmental...
positive regulation of cytokine...
cell chemotaxis
maintenance of location
regulation of supramolecular fiber...
chemotaxis
actin filament organization
external side of plasma...
extrinsic component of membrane
transferase complex, transferring phosph...
nuclear envelope
cytoplasmic side of plasma...
cytoplasmic side of membrane
nuclear membrane
focal adhesion
cell-substrate junction
heterotrimeric G-protein complex
GTPase complex
extrinsic component of cytoplasmic...
cyclin-dependent protein kinase holoenzy...
Schaffer collateral - CA1...
sarcoplasm
protein serine/threonine kinase activity
kinase regulator activity
protein serine kinase activity
DNA-binding transcription factor binding
cytokine receptor binding
ubiquitin protein ligase binding
ubiquitin-like protein ligase binding
RNA polymerase II-specific DNA-binding...
amide binding
cyclin-dependent protein serine/threonin...
protein kinase regulator activity
peptide binding
phosphoric ester hydrolase activity
adenylate cyclase activity
MAP kinase activity

P

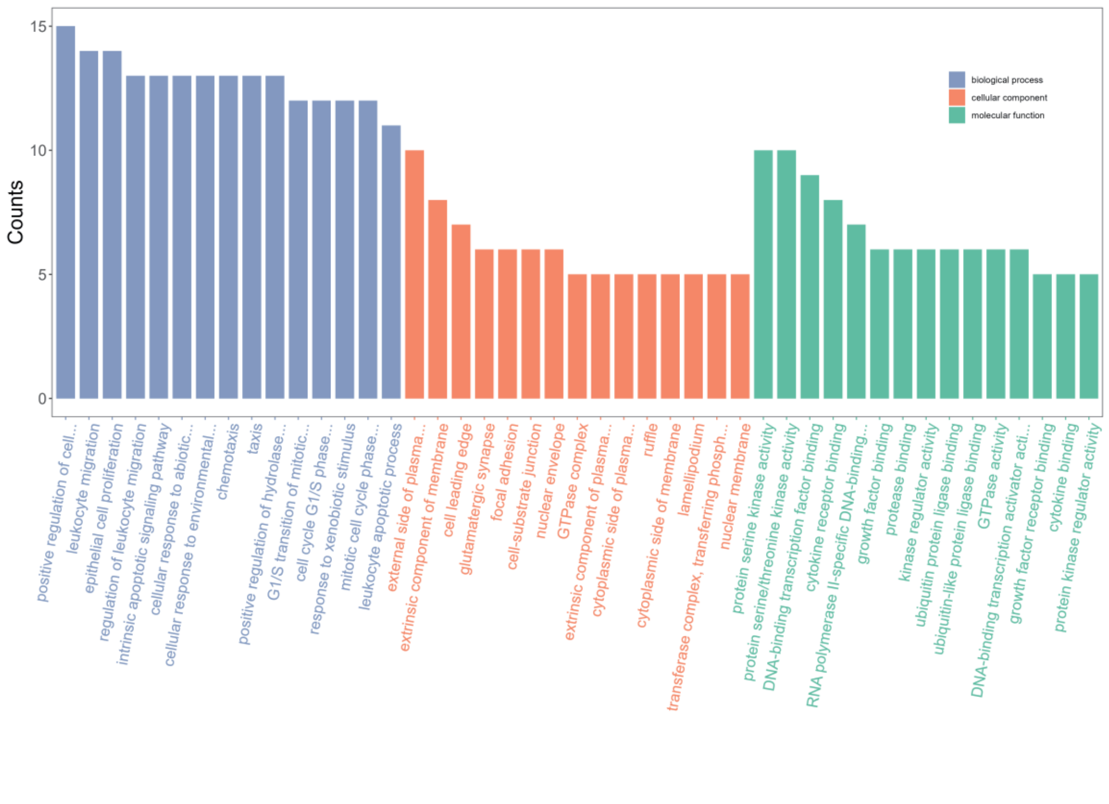

Counts
15
10
5
0
biological process
cellular component
molecular function
positive regulation of cell...
leukocyte migration
epithelial cell proliferation
regulation of leukocyte migration
intrinsic apoptotic signaling pathway
cellular response to abiotic...
cellular response to environmental...
chemotaxis
taxis
positive regulation of hydrolase...
G1/S transition of mitotic...
cell cycle G1/S phase...
response to xenobiotic stimulus
mitotic cell cycle phase...
leukocyte apoptotic process
external side of plasma...
extrinsic component of membrane
cell leading edge
glutamatergic synapse
focal adhesion
cell-substrate junction
nuclear envelope
GTPase complex
extrinsic component of plasma...
cytoplasmic side of plasma...
ruffle
cytoplasmic side of membrane
lamellipodium
transferase complex, transferring phosph...
nuclear membrane
protein serine kinase activity
protein serine/threonine kinase activity
DNA-binding transcription factor binding
cytokine receptor binding
RNA polymerase II-specific DNA-binding...
growth factor binding
protease binding
kinase regulator activity
ubiquitin protein ligase binding
ubiquitin-like protein ligase binding
GTPase activity
DNA-binding transcription activator acti...
growth factor receptor binding
cytokine binding
protein kinase regulator activity

Q

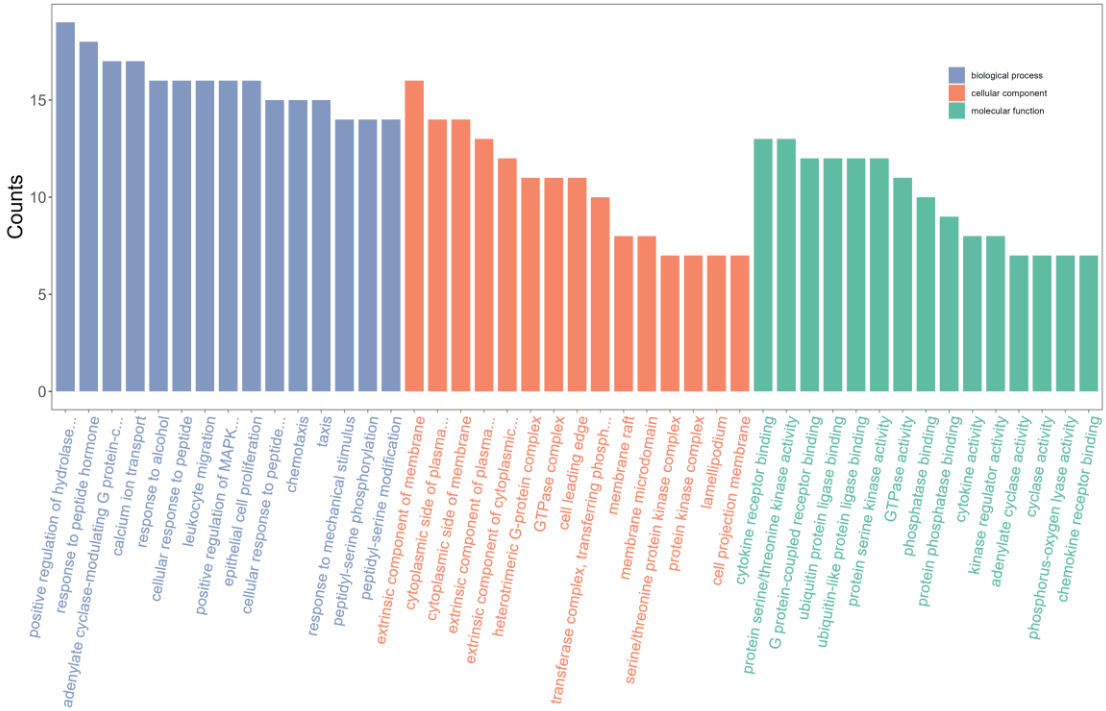
Counts
0
5
10
15
biological process
cellular component
molecular function
positive regulation of hydrolase...
response to peptide hormone
adenylate cyclase-modulating G protein-c...
calcium ion transport
response to alcohol
cellular response to peptide
leukocyte migration
positive regulation of MAPK...
epithelial cell proliferation
cellular response to peptide...
chemotaxis
taxis
response to mechanical stimulus
peptidyl-serine phosphorylation
peptidyl-serine modification
extrinsic component of membrane
cytoplasmic side of plasma...
cytoplasmic side of membrane
extrinsic component of plasma...
extrinsic component of cytoplasmic...
heterotrimeric G-protein complex
GTPase complex
cell leading edge
transferase complex, transferring phosph...
membrane raft
membrane microdomain
serine/threonine protein kinase complex
protein kinase complex
lamellipodium
cell projection membrane
cytokine receptor binding
protein serine/threonine kinase activity
G protein-coupled receptor binding
ubiquitin protein ligase binding
ubiquitin-like protein ligase binding
protein serine kinase activity
GTPase activity
phosphatase binding
protein phosphatase binding
cytokine activity
kinase regulator activity
adenylate cyclase activity
cyclase activity
phosphorus-oxygen lyase activity
chemokine receptor binding

R

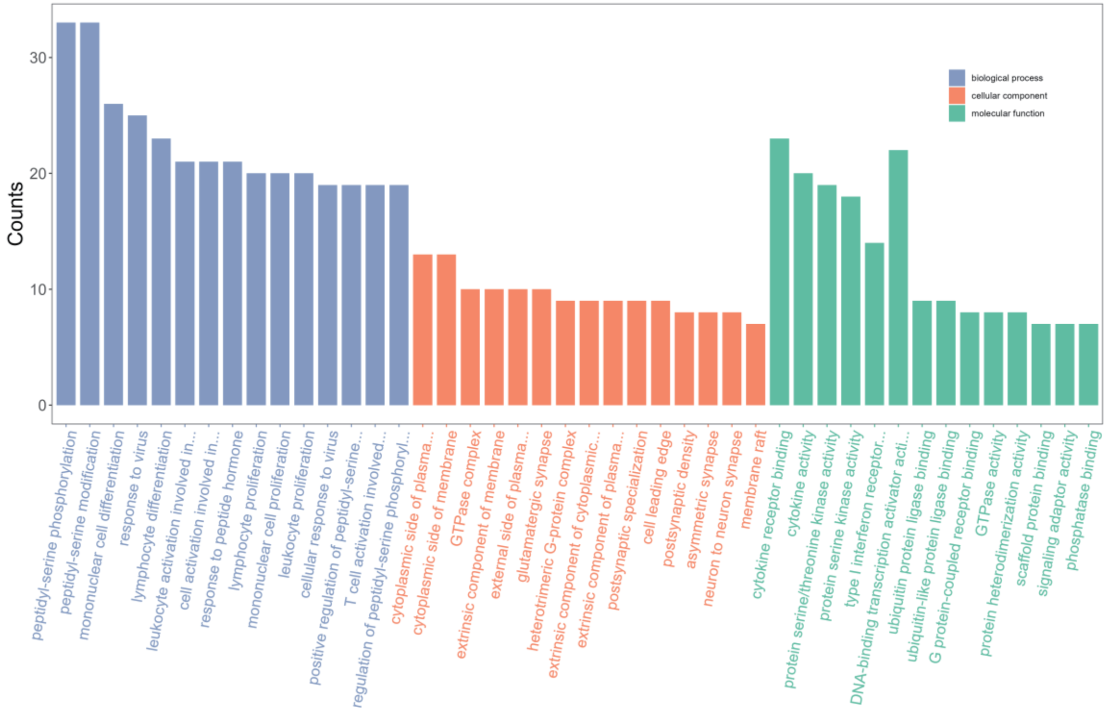
Counts
0
10
20
30
biological process
cellular component
molecular function
peptidyl-serine phosphorylation
peptidyl-serine modification
mononuclear cell differentiation
response to virus
lymphocyte differentiation
leukocyte activation involved in...
cell activation involved in...
response to peptide hormone
lymphocyte proliferation
mononuclear cell proliferation
leukocyte proliferation
cellular response to virus
positive regulation of peptidyl-serine...
T cell activation involved...
regulation of peptidyl-serine phosphoryl...
cytoplasmic side of plasma...
cytoplasmic side of membrane
GTPase complex
extrinsic component of membrane
external side of plasma...
glutamatergic synapse
heterotrimeric G-protein complex
extrinsic component of cytoplasmic...
extrinsic component of plasma...
postsynaptic specialization
cell leading edge
postsynaptic density
asymmetric synapse
neuron to neuron synapse
membrane raft
cytokine receptor binding
cytokine activity
protein serine/threonine kinase activity
protein serine kinase activity
type I interferon receptor...
DNA-binding transcription activator acti...
ubiquitin protein ligase binding
ubiquitin-like protein ligase binding
G protein-coupled receptor binding
GTPase activity
protein heterodimerization activity
scaffold protein binding
signaling adaptor activity
phosphatase binding

S

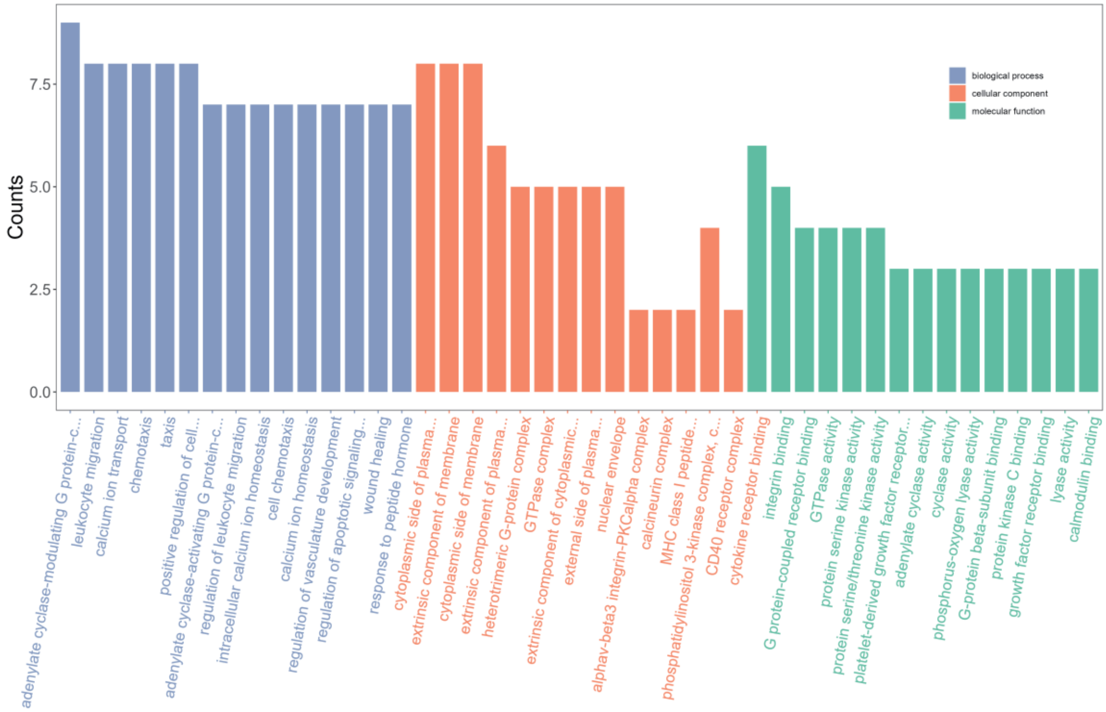
Counts
biological process
cellular component
molecular function
adenylate cyclase-modulating G protein-c...
leukocyte migration
calcium ion transport
chemotaxis
taxis
positive regulation of cell...
adenylate cyclase-activating G protein-c...
regulation of leukocyte migration
intracellular calcium ion homeostasis
cell chemotaxis
calcium ion homeostasis
regulation of vasculature development
regulation of apoptotic signaling...
wound healing
response to peptide hormone
cytoplasmic side of plasma...
extrinsic component of membrane
cytoplasmic side of membrane
extrinsic component of plasma...
heterotrimeric G-protein complex
GTPase complex
extrinsic component of cytoplasmic...
external side of plasma...
nuclear envelope
alphav-beta3 integrin-PKCalpha complex
calcineurin complex
MHC class I peptide...
phosphatidylinositol 3-kinase complex, c...
CD40 receptor complex
cytokine receptor binding
integrin binding
G protein-coupled receptor binding
GTPase activity
protein serine kinase activity
protein serine/threonine kinase activity
platelet-derived growth factor receptor...
adenylate cyclase activity
cyclase activity
phosphorus-oxygen lyase activity
G-protein beta-subunit binding
protein kinase C binding
growth factor receptor binding
lyase activity
calmodulin binding

T

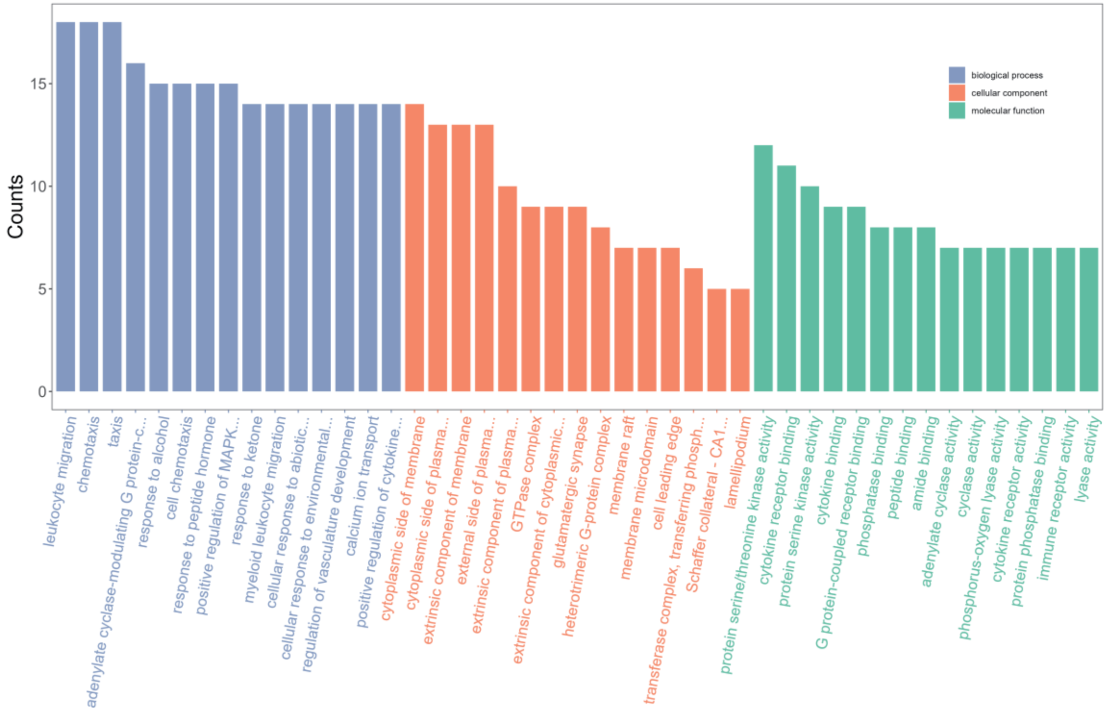
Counts
biological process
cellular component
molecular function
leukocyte migration
chemotaxis
taxis
adenylate cyclase-modulating G protein-c...
response to alcohol
cell chemotaxis
response to peptide hormone
positive regulation of MAPK...
response to ketone
myeloid leukocyte migration
cellular response to abiotic...
cellular response to environmental...
regulation of vasculature development
calcium ion transport
positive regulation of cytokine...
cytoplasmic side of membrane
cytoplasmic side of plasma...
extrinsic component of membrane
external side of plasma...
extrinsic component of plasma...
GTPase complex
extrinsic component of cytoplasmic...
glutamatergic synapse
heterotrimeric G-protein complex
membrane raft
membrane microdomain
cell leading edge
transferase complex, transferring phosph...
Schaffer collateral - CA1...
lamellipodium
protein serine/threonine kinase activity
cytokine receptor binding
protein serine kinase activity
cytokine binding
G protein-coupled receptor binding
phosphatase binding
peptide binding
amide binding
adenylate cyclase activity
cyclase activity
phosphorus-oxygen lyase activity
cytokine receptor activity
protein phosphatase binding
immune receptor activity
lyase activity

U

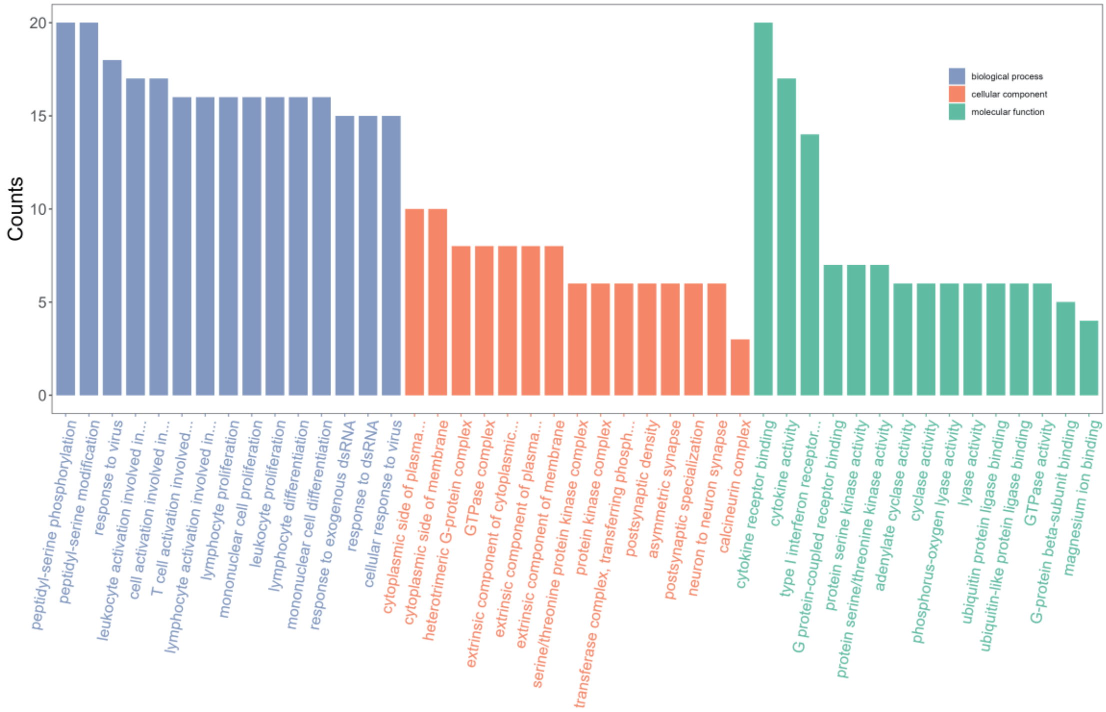
Counts
0
5
10
15
20
biological process
cellular component
molecular function
peptidyl-serine phosphorylation
peptidyl-serine modification
response to virus
leukocyte activation involved in...
cell activation involved in...
T cell activation involved in...
lymphocyte activation involved in...
lymphocyte proliferation
mononuclear cell proliferation
leukocyte proliferation
lymphocyte differentiation
mononuclear cell differentiation
response to exogenous dsRNA
response to dsRNA
cellular response to virus
cytoplasmic side of plasma...
cytoplasmic side of membrane
heterotrimeric G-protein complex
GTPase complex
extrinsic component of cytoplasmic...
extrinsic component of plasma...
extrinsic component of membrane
serine/threonine protein kinase complex
protein kinase complex
transferase complex, transferring phosph...
postsynaptic density
asymmetric synapse
postsynaptic specialization
neuron to neuron synapse
calcineurin complex
cytokine receptor binding
cytokine activity
type I interferon receptor...
G protein-coupled receptor binding
protein serine kinase activity
protein serine/threonine kinase activity
adenylate cyclase activity
cyclase activity
phosphorus-oxygen lyase activity
lyase activity
ubiquitin protein ligase binding
ubiquitin-like protein ligase binding
GTPase activity
G-protein beta-subunit binding
magnesium ion binding

V

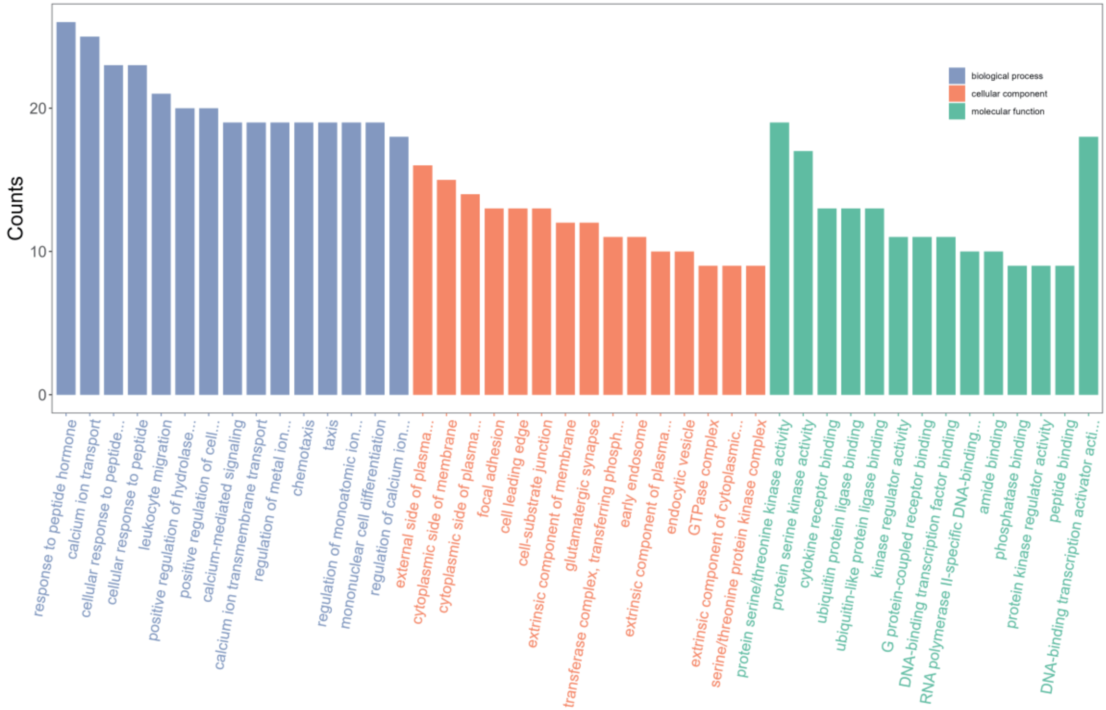
Counts
0
10
20
biological process
cellular component
molecular function
response to peptide hormone
calcium ion transport
cellular response to peptide...
cellular response to peptide
leukocyte migration
positive regulation of hydrolase...
positive regulation of cell...
calcium-mediated signaling
calcium ion transmembrane transport
regulation of metal ion...
chemotaxis
taxis
regulation of monoatomic ion...
mononuclear cell differentiation
regulation of calcium ion...
external side of plasma...
cytoplasmic side of membrane
cytoplasmic side of plasma...
focal adhesion
cell leading edge
cell-substrate junction
extrinsic component of membrane
glutamatergic synapse
transferase complex, transferring phosph...
early endosome
extrinsic component of plasma...
endocytic vesicle
GTPase complex
extrinsic component of cytoplasmic...
serine/threonine protein kinase complex
protein serine/threonine kinase activity
protein serine kinase activity
cytokine receptor binding
ubiquitin protein ligase binding
ubiquitin-like protein ligase binding
kinase regulator activity
G protein-coupled receptor binding
DNA-binding transcription factor binding
RNA polymerase II-specific DNA-binding...
amide binding
phosphatase binding
protein kinase regulator activity
peptide binding
DNA-binding transcription activator acti...

W

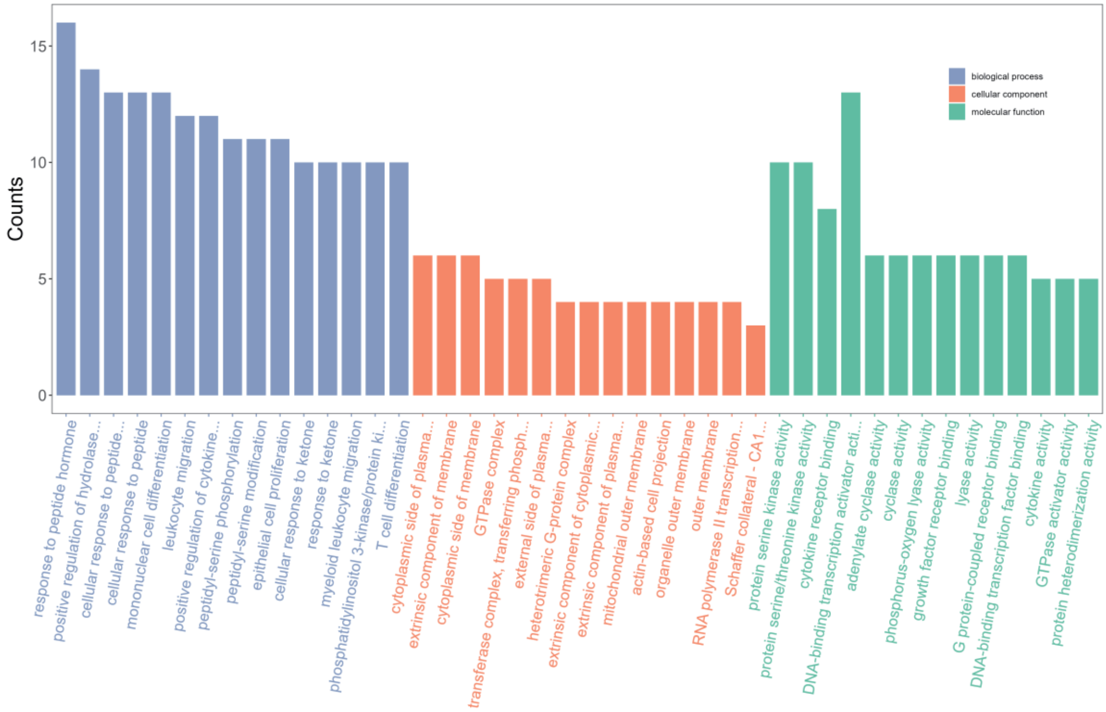
Counts
0
5
10
15
biological process
cellular component
molecular function
response to peptide hormone
positive regulation of hydrolase...
cellular response to peptide...
cellular response to peptide
mononuclear cell differentiation
leukocyte migration
positive regulation of cytokine...
peptidyl-serine phosphorylation
peptidyl-serine modification
epithelial cell proliferation
cellular response to ketone
response to ketone
myeloid leukocyte migration
phosphatidylinositol 3-kinase/protein ki...
T cell differentiation
cytoplasmic side of plasma...
extrinsic component of membrane
cytoplasmic side of membrane
GTPase complex
transferase complex, transferring phosph...
external side of plasma...
heterotrimeric G-protein complex
extrinsic component of cytoplasmic...
extrinsic component of plasma...
mitochondrial outer membrane
actin-based cell projection
organelle outer membrane
outer membrane
RNA polymerase II transcription...
Schaffer collateral - CA1...
protein serine kinase activity
protein serine/threonine kinase activity
cytokine receptor binding
DNA-binding transcription activator acti...
adenylate cyclase activity
cyclase activity
phosphorus-oxygen lyase activity
growth factor receptor binding
lyase activity
G protein-coupled receptor binding
DNA-binding transcription factor binding
cytokine activity
GTPase activator activity
protein heterodimerization activity

X

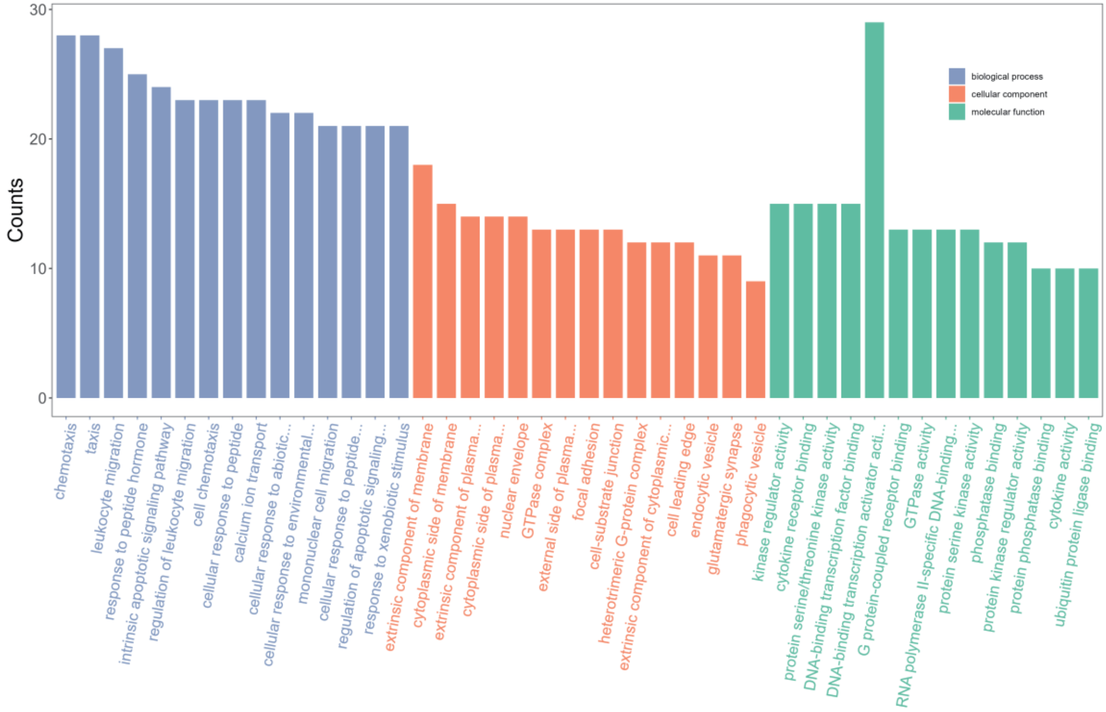
Counts
0
10
20
30
biological process
cellular component
molecular function
chemotaxis
taxis
leukocyte migration
response to peptide hormone
intrinsic apoptotic signaling pathway
regulation of leukocyte migration
cell chemotaxis
cellular response to peptide
calcium ion transport
cellular response to abiotic...
cellular response to environmental...
mononuclear cell migration
cellular response to peptide...
regulation of apoptotic signaling...
response to xenobiotic stimulus
extrinsic component of membrane
cytoplasmic side of membrane
extrinsic component of plasma...
cytoplasmic side of plasma...
nuclear envelope
GTPase complex
external side of plasma...
focal adhesion
cell-substrate junction
heterotrimeric G-protein complex
extrinsic component of cytoplasmic...
cell leading edge
endocytic vesicle
glutamatergic synapse
phagocytic vesicle
kinase regulator activity
cytokine receptor binding
protein serine/threonine kinase activity
DNA-binding transcription factor binding
DNA-binding transcription activator acti...
G protein-coupled receptor binding
GTPase activity
RNA polymerase II-specific DNA-binding...
protein serine kinase activity
phosphatase binding
protein kinase regulator activity
protein phosphatase binding
cytokine activity
ubiquitin protein ligase binding

Y

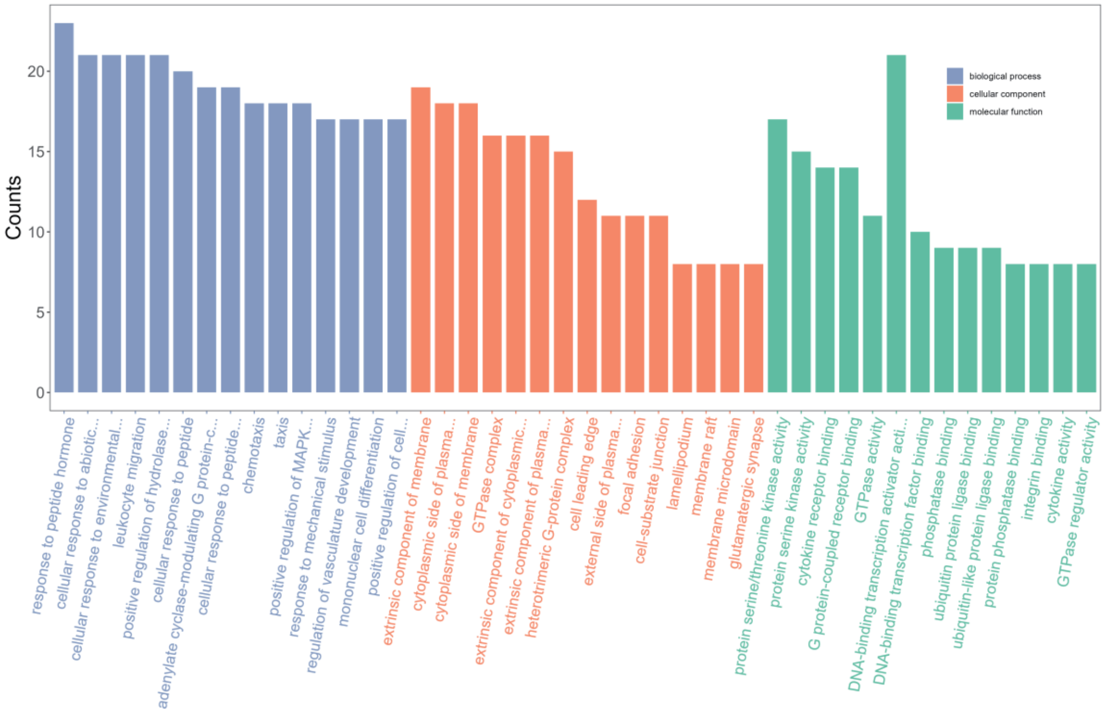

Z

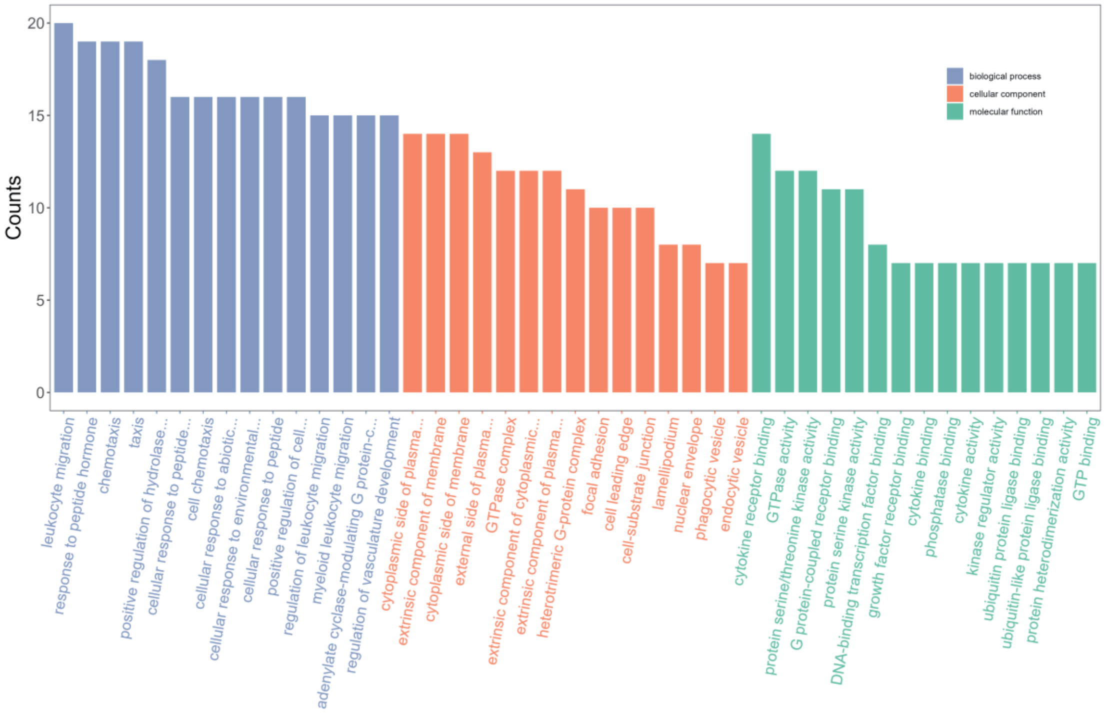

GO enrichment analyses in (A) ACC, (B) BLCA, (C) BRCA, (D) CHOL, (E) DLBC, (F) ESCA, (G) GBM, (H) HNSC, (I) KICH, (J) KIRC, (K) KIRP, (L) LAML, (M) LGG, (N) LIHC, (O) LUAD, (P) LUSC, (Q) OV, (R) PAAD, (S) PRAD, (T) SKCM, (U) STAD, (V) TGCT, (W) THCA, (X) THYM, (Y) UCEC, and (Z) UCS: The horizontal axis represents the potential functions enriched, while the vertical axis indicates the number of enrichments (from left to right, they are Biological Process (BP) in purple, Cellular Component (CC) in orange, and Molecular Function (MF) in green).
