# Supplementary material for: Comprehensive bioinformatics analysis of human cytomegalovirus pathway genes in pan-cancer
Source: Hum Genomics. 2024 Jun 17;18:65. doi: 10.1186/s40246-024-00633-5 (PMC11181644; doi:10.1186/s40246-024-00633-5)
Supplement: Supplementary file 9 — Supplementary Material 9 [file 40246_2024_633_MOESM9_ESM.pdf]

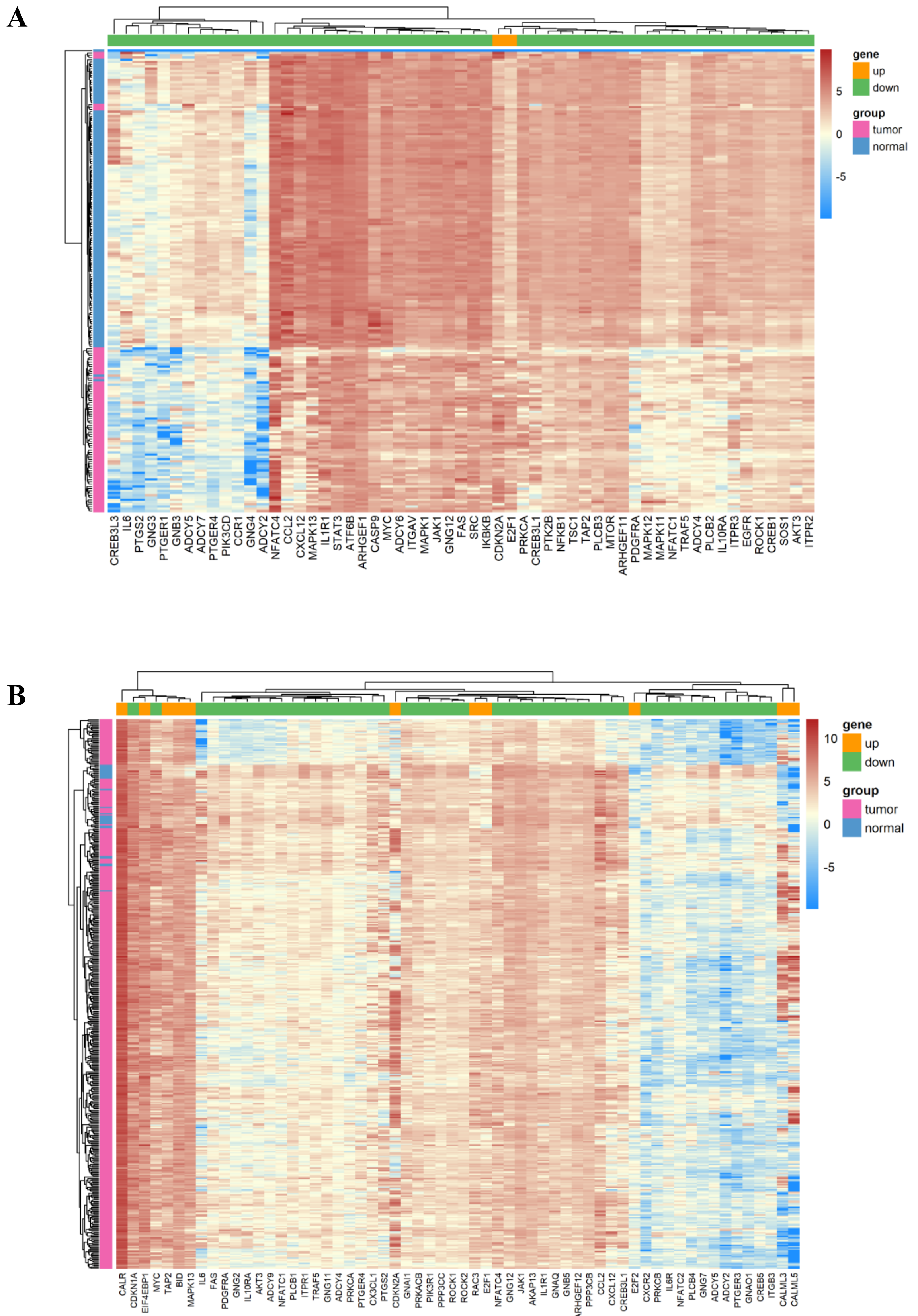
A
gene
up
down
group
tumor
normal
5
0
-5
CREB3L3 IL6 PTGS2 GNG3 PTGER1 GNB3 ADCY5 ADCY7 PTGER4 PIK3CD CCR1 GNG4 ADCY2 NFATC4 CCL2 CXCL12 MAPK13 IL1R1 STAT3 ATF6B ARHGEF1 CASP9 MYC ADCY6 ITGAV MAPK1 JAK1 GNG12 FAS SRC IKBKB CDKN2A E2F1 PRKCA CREB3L1 PTK2B NFKB1 TSC1 TAP2 PLCB3 MTOR ARHGEF11 PDGFRA MAPK12 MAPK11 NFATC1 TRAF5 ADCY4 PLCB2 IL10RA ITPR3 EGFR ROCK1 CREB1 SOS1 AKT3 ITPR2
B
gene
up
down
group
tumor
normal
10
5
0
-5
CALR CDKN1A EIF4EBP1 MYC TAP2 BID MAPK13 IL6 FAS PDGFRA GNG2 IL10RA AKT3 ADCY9 NFATC1 PLCB1 ITPR1 TRAF5 GNG11 ADCY4 PRKCA PTGER4 CX3CL1 PTGS2 CDKN2A GNAI1 PRKACB PIK3R1 PPP3CC ROCK1 ROCK2 RAC3 E2F1 NFATC4 GNG12 JAK1 AKAP13 IL1R1 GNAQ GNB5 ARHGEF12 PPP3CB CCL2 CXCL12 CREB3L1 E2F2 CXCR2 PRKCB IL6R NFATC2 PLCB4 GNG7 ADCY5 ADCY2 PTGER3 GNAO1 CREB5 ITGB3 CALML3 CALML5

C

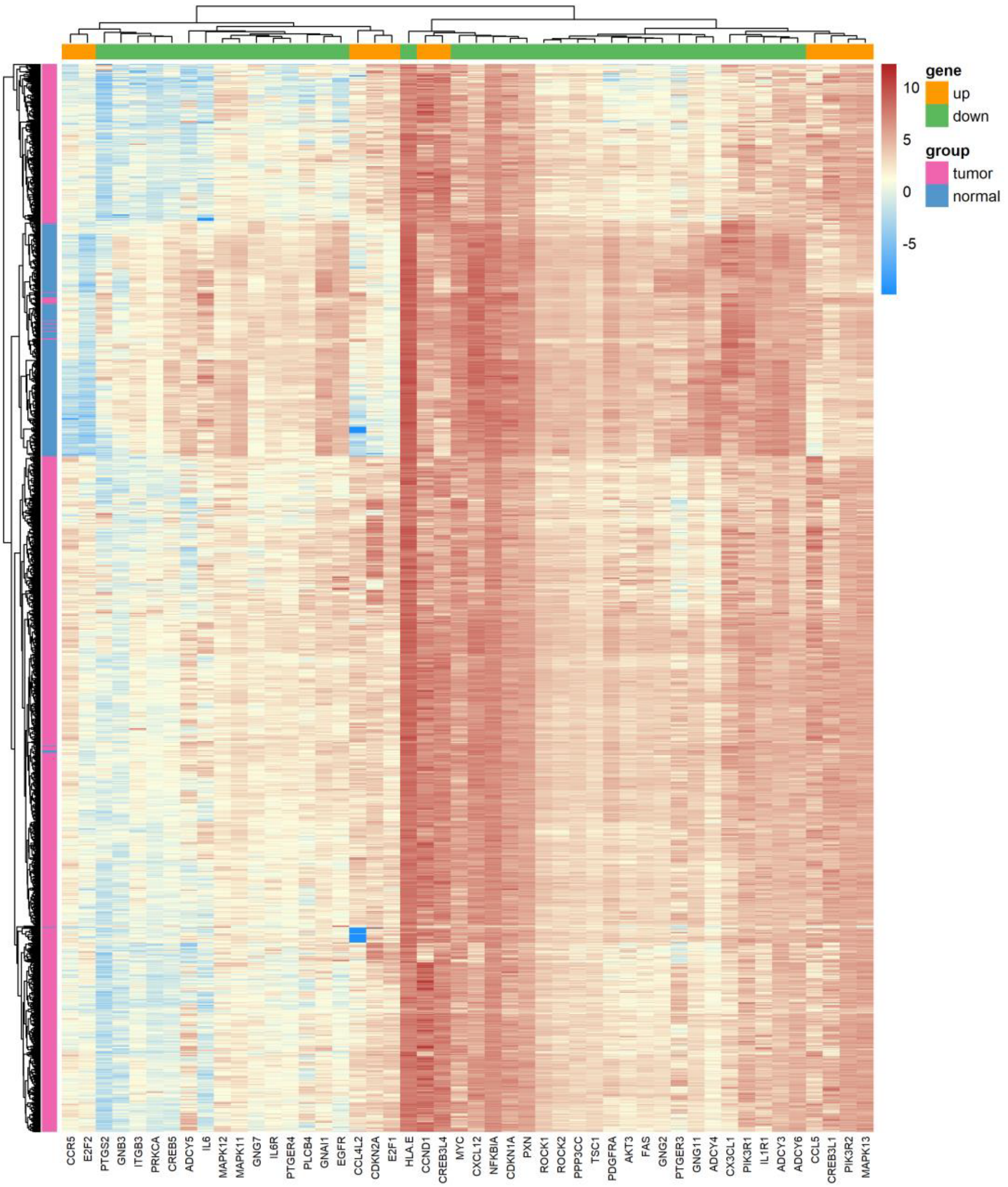
gene
up
down
group
tumor
normal
10
5
0
-5
CCR5
E2F2
PTGS2
GNB3
ITGB3
PRKCA
CREB5
ADCY5
IL6
MAPK12
MAPK11
GNG7
IL6R
PTGER4
PLCB4
GNAI1
EGFR
CCL4L2
CDKN2A
E2F1
HLA.E
CCND1
CREB3L4
MYC
CXCL12
NFKBIA
CDKN1A
PXN
ROCK1
ROCK2
PPP3CC
TSC1
PDGFRA
AKT3
FAS
GNG2
PTGER3
GNG11
ADCY4
CX3CL1
PIK3R1
IL1R1
ADCY3
ADCY6
CCL5
CREB3L1
PIK3R2
MAPK13

D

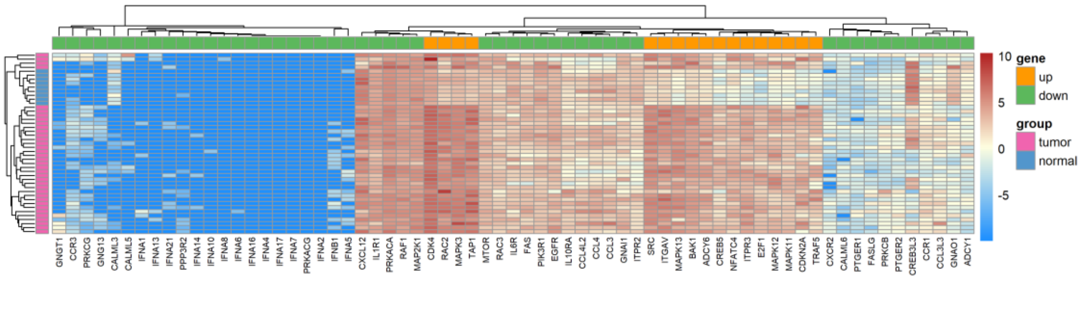
gene
up
down
group
tumor
normal
10
5
0
-5
GNGT1
CCR3
PRKCG
GNG13
CALML3
CALML5
IFNA1
IFNA13
IFNA21
PPP3R2
IFNA14
IFNA10
IFNA8
IFNA6
IFNA16
IFNA4
IFNA17
IFNA7
PRKACG
IFNA2
IFNB1
IFNA5
CXCL12
IL1R1
PRKACA
RAF1
MAP2K1
CDK4
RAC2
MAPK3
TAP1
MTOR
RAC3
IL6R
FAS
PIK3R1
EGFR
IL10RA
CCL4L2
CCL4
CCL3
GNAI1
ITPR2
SRC
ITGAV
MAPK13
BAK1
ADCY6
CREB5
NFATC4
ITPR3
E2F1
MAPK12
MAPK11
CDKN2A
TRAF5
CXCR2
CALML6
PTGER1
FASLG
PRKCB
PTGER2
CREB3L3
CCR1
CCL3L3
GNAO1
ADCY1

E

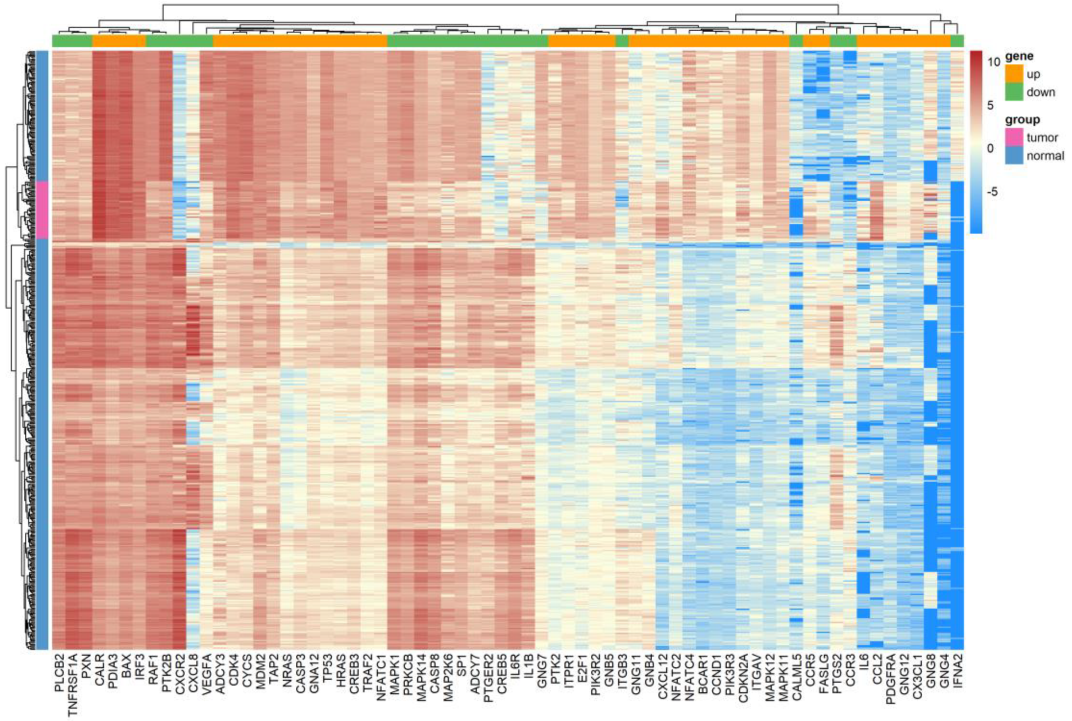
10
5
0
-5
gene
up
down
group
tumor
normal
PLCB2
TNFRSF1A
PXN
CALR
PDIA3
BAX
IRF3
RAF1
PTK2B
CXCR2
CXCL8
VEGFA
ADCY3
CDK4
CYCS
MDM2
TAP2
NRAS
CASP3
GNA12
TP53
HRAS
CREB3
TRAF2
NFATC1
MAPK1
PRKCB
MAPK14
CASP8
MAP2K6
SP1
ADCY7
PTGER2
CREB5
IL6R
IL1B
GNG7
PTK2
ITPR1
E2F1
PIK3R2
GNB5
ITGB3
GNG11
GNB4
CXCL12
NFATC2
NFATC4
BCAR1
CCND1
PIK3R3
CDKN2A
ITGAV
MAPK12
MAPK11
CALML5
CCR5
FASLG
PTGS2
CCR3
IL6
CCL2
PDGFRA
GNG12
CX3CL1
GNG8
GNG4
IFNA2

F

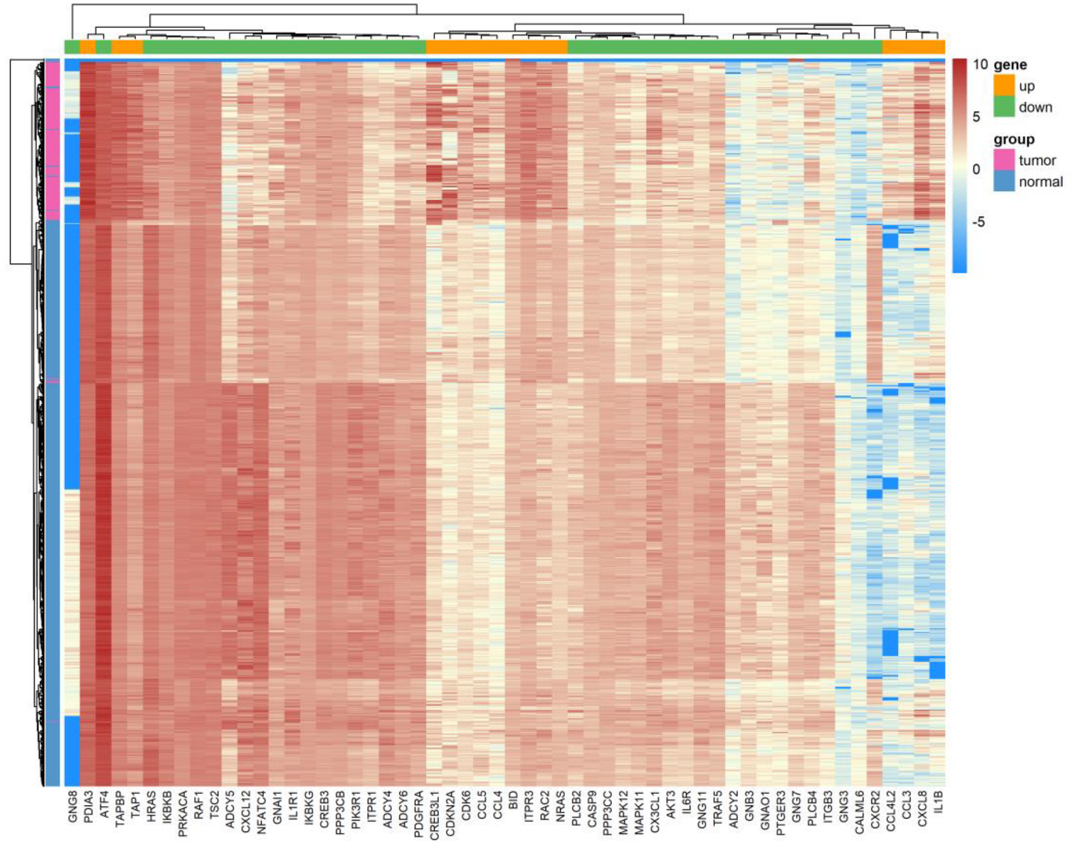
10
5
0
-5
gene
up
down
group
tumor
normal
GNG8
PDIA3
ATF4
TAPBP
TAP1
HRAS
IKBKB
PRKACA
RAF1
TSC2
ADCY5
CXCL12
NFATC4
GNAI1
IL1R1
IKBKG
CREB3
PPP3CB
PIK3R1
ITPR1
ADCY4
ADCY6
PDGFRA
CREB3L1
CDKN2A
CDK6
CCL5
CCL4
BID
ITPR3
RAC2
NRAS
PLCB2
CASP9
PPP3CC
MAPK12
MAPK11
CX3CL1
AKT3
IL6R
GNG11
TRAF5
ADCY2
GNB3
GNAO1
PTGER3
GNG7
PLCB4
ITGB3
GNG3
CALML6
CXCR2
CCL4L2
CCL3
CXCL8
IL1B

G

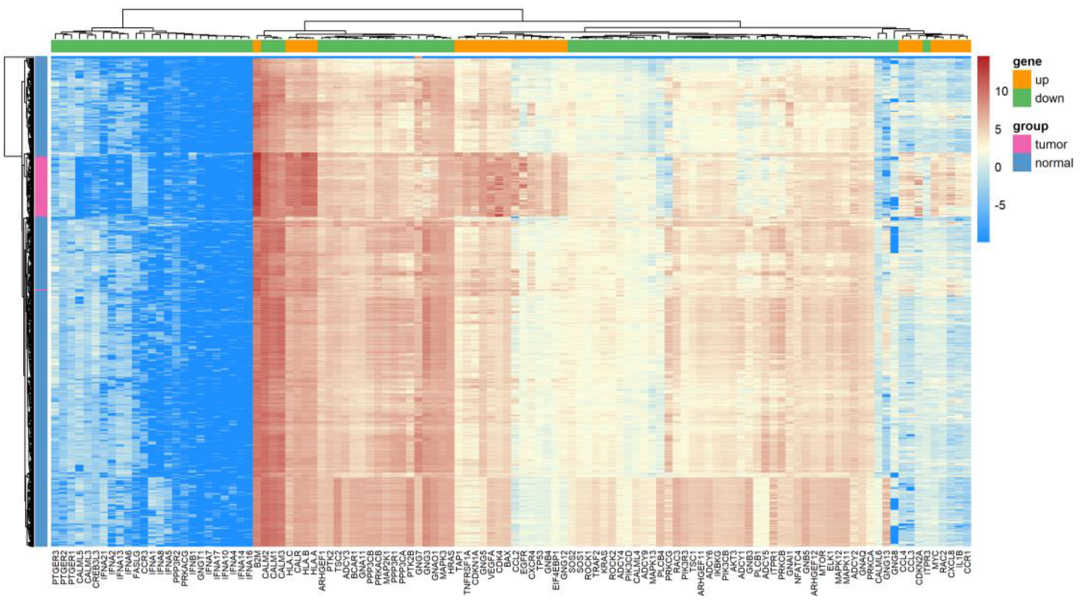
gene
up
down
group
tumor
normal
10
5
0
-5

H

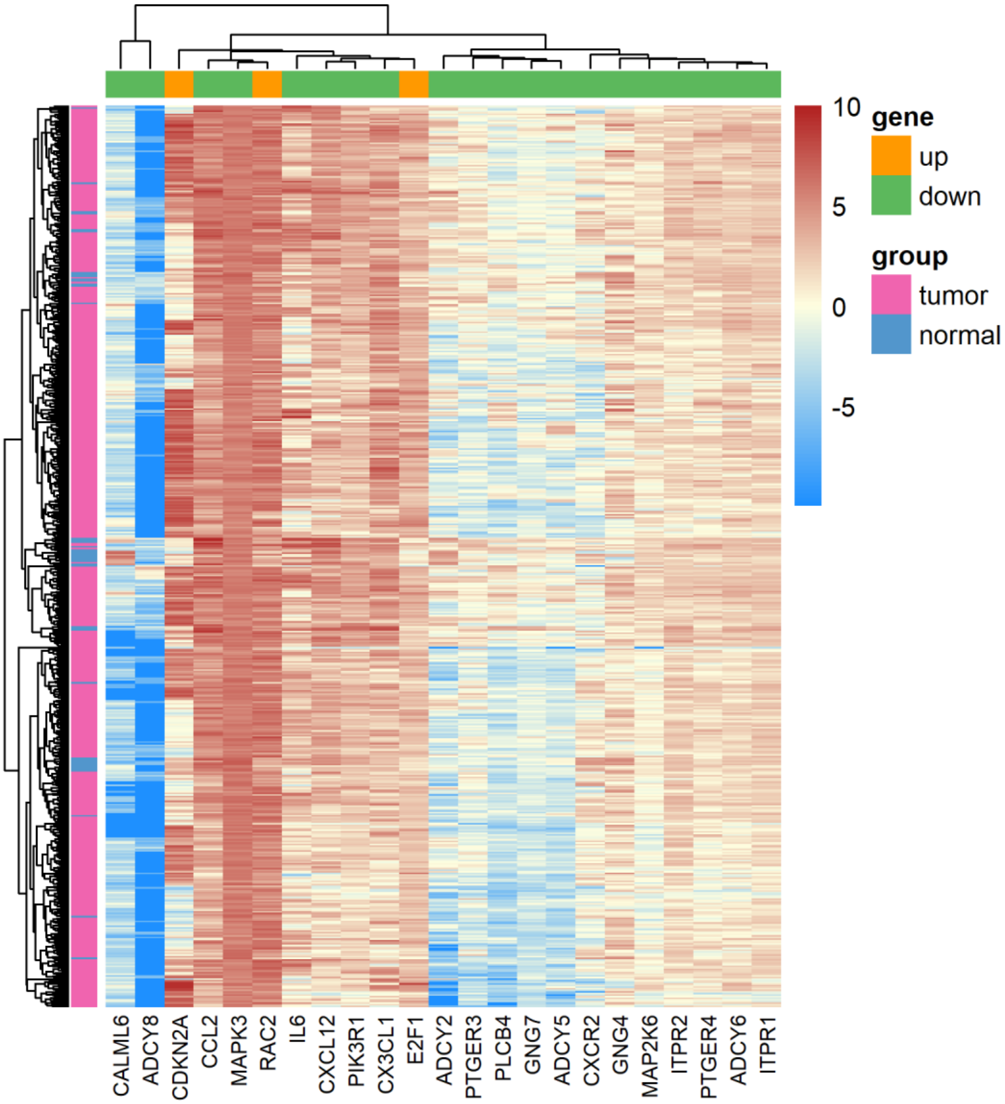
gene
up
down
group
tumor
normal
10
5
0
-5
CALML6
ADCY8
CDKN2A
CCL2
MAPK3
RAC2
IL6
CXCL12
PIK3R1
CX3CL1
E2F1
ADCY2
PTGER3
PLCB4
GNG7
ADCY5
CXCR2
GNG4
MAP2K6
ITPR2
PTGER4
ADCY6
ITPR1

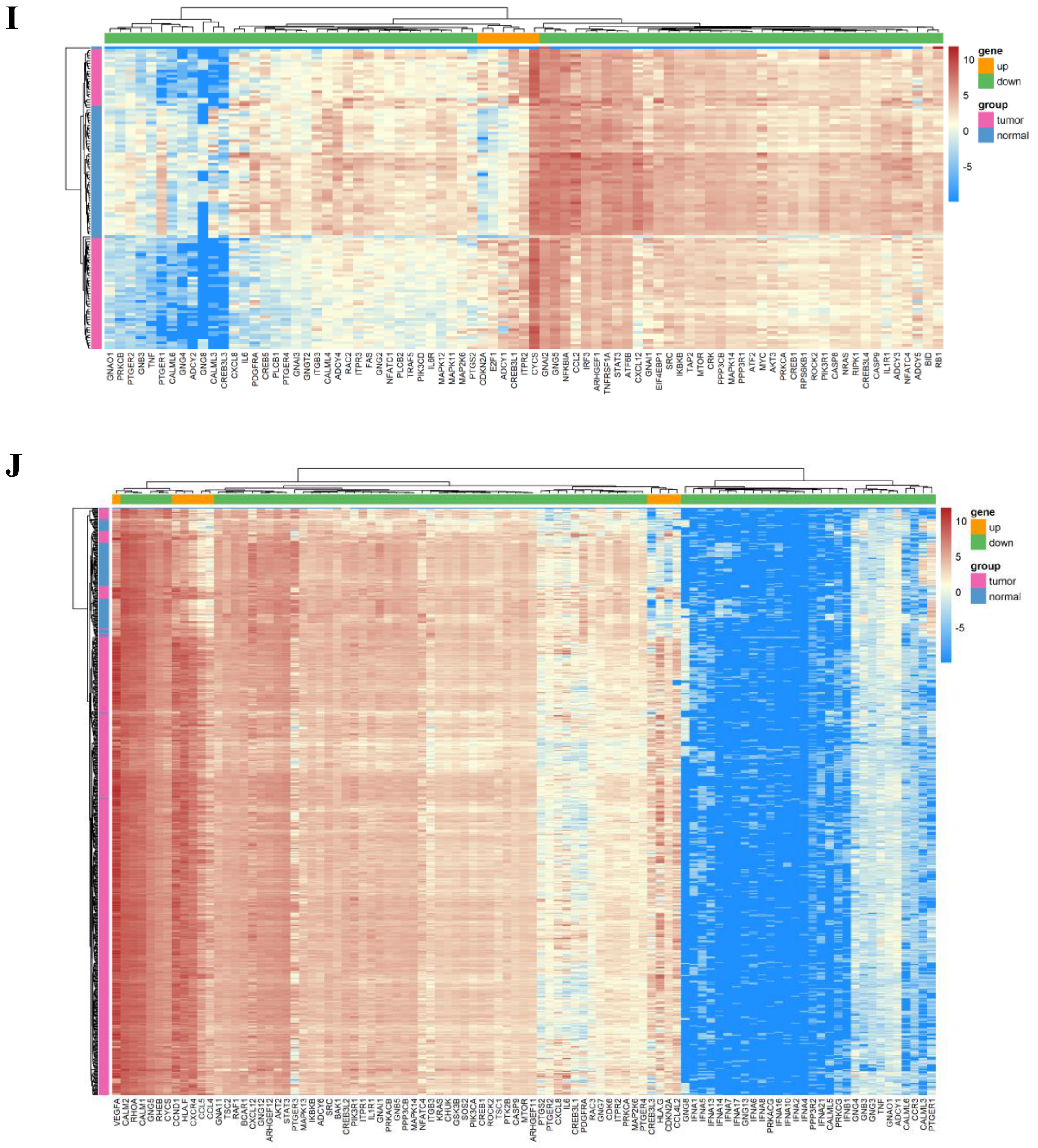
I
gene
up
down
group
tumor
normal
10
5
0
-5
GNAO1 PRKCB PTGER2 GNB3 TNF PTGER1 CALML6 GNG4 ADCY2 GNG8 CALML3 CREB3L3 CXCL8 IL6 PDGFRA CREB5 PLCB1 PTGER4 GNAI3 GNGT2 ITGB3 CALML4 ADCY4 RAC2 ITPR3 FAS GNG2 NFATC1 PLCB2 TRAF5 PIK3CD IL6R MAPK12 MAPK11 MAP2K6 PTGS2 CDKN2A E2F1 ADCY1 CREB3L1 ITPR2 CYCS GNAI2 GNG5 NFKBIA CCL2 IRF3 ARHGEF1 TNFRSF1A STAT3 ATF6B CXCL12 GNAI1 EIF4EBP1 SRC IKBKB TAP2 MTOR CRK PPP3CB MAPK14 PPP3R1 ATF2 MYC AKT3 PRKCA CREB1 RPS6KB1 ROCK2 PIK3R1 CASP8 NRAS RIPK1 CREB3L4 CASP9 IL1R1 ADCY3 NFATC4 ADCY5 BID RB1
J
gene
up
down
group
tumor
normal
10
5
0
-5
VEGFA CALM2 RHOA CALM1 GNG5 RHEB CYCS CCND1 HLA.F CXCR4 CCL5 CCL4 GNA11 TSC2 RAF1 BCAR1 CXCL12 GNG12 ARHGEF12 AKT2 STAT3 PTGER3 MAPK13 IKBKB ADCY6 SRC BAK1 CREB3L2 PIK3R1 ITPR1 IL1R1 GNAI1 PRKACB GNB5 PPP3CB MAPK14 NFATC4 ITGB3 KRAS CHUK GSK3B SOS2 PIK3CA CREB1 ROCK2 TSC1 PTK2B CASP9 MTOR ARHGEF11 PTGS2 PTGER2 CXCL8 IL6 CREB3L1 PDGFRA RAC3 GNG7 CDK6 ITPR2 PRKCA MAP2K6 PTGER4 CREB3L3 HLA.G CDKN2A CCL4L2 GNG8 IFNA1 IFNA5 IFNA13 IFNA14 IFNA7 IFNA17 GNG13 IFNA6 IFNA8 PRKACG IFNA16 IFNA10 IFNA2 IFNA4 PPP3R2 IFNA21 CALML5 PRKCG IFNB1 GNG4 GNB3 TNF GNAO1 ADCY1 CALML6 CCR3 CALML3 PTGER1

K

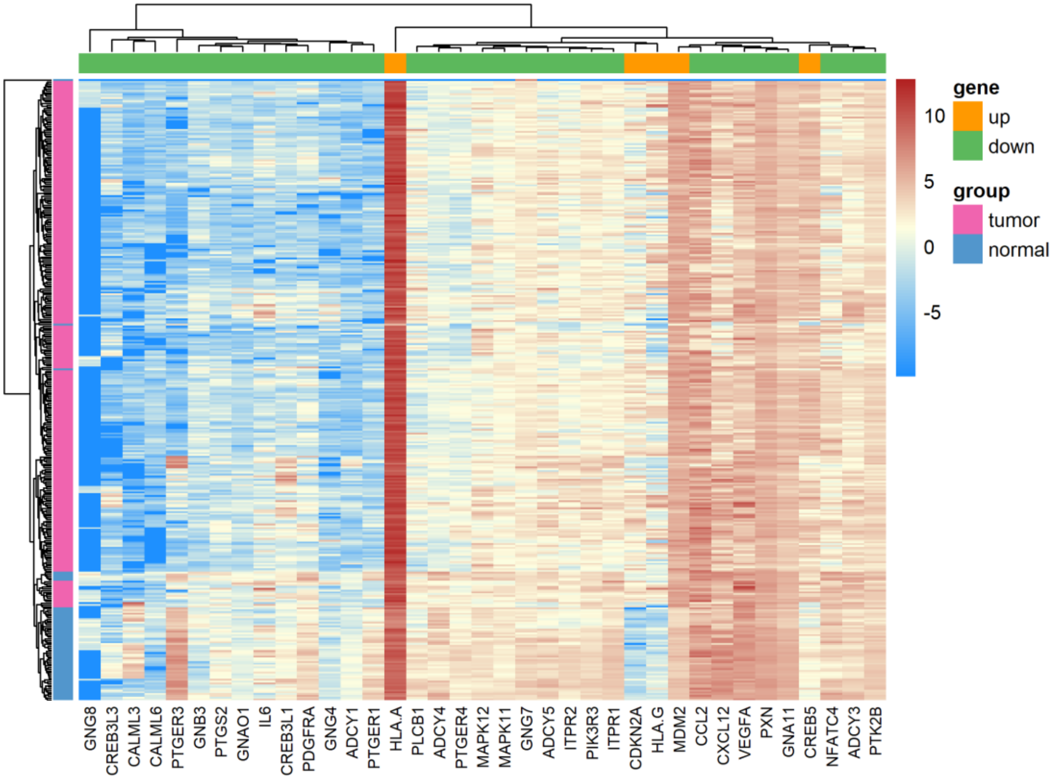
gene
up
down
group
tumor
normal
10
5
0
-5
GNG8
CREB3L3
CALML3
CALML6
PTGER3
GNB3
PTGS2
GNAO1
IL6
CREB3L1
PDGFRA
GNG4
ADCY1
PTGER1
HLA.A
PLCB1
ADCY4
PTGER4
MAPK12
MAPK11
GNG7
ADCY5
ITPR2
PIK3R3
ITPR1
CDKN2A
HLA.G
MDM2
CCL2
CXCL12
VEGFA
PXN
GNA11
CREB5
NFATC4
ADCY3
PTK2B

L

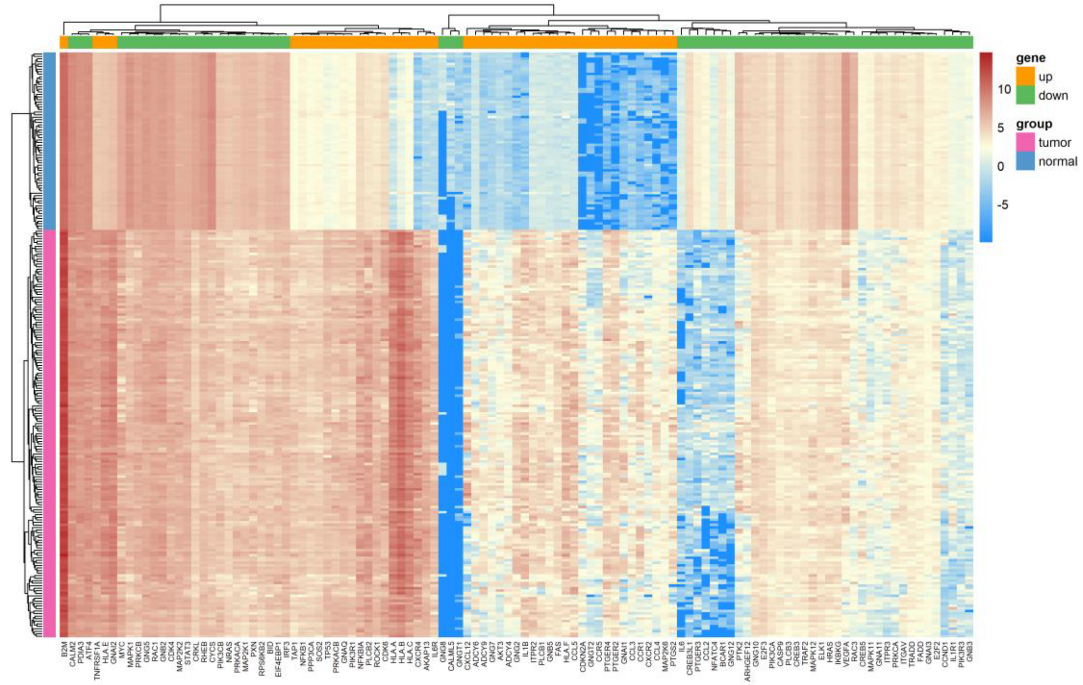
gene
up
down
group
tumor
normal
10
5
0
-5

M

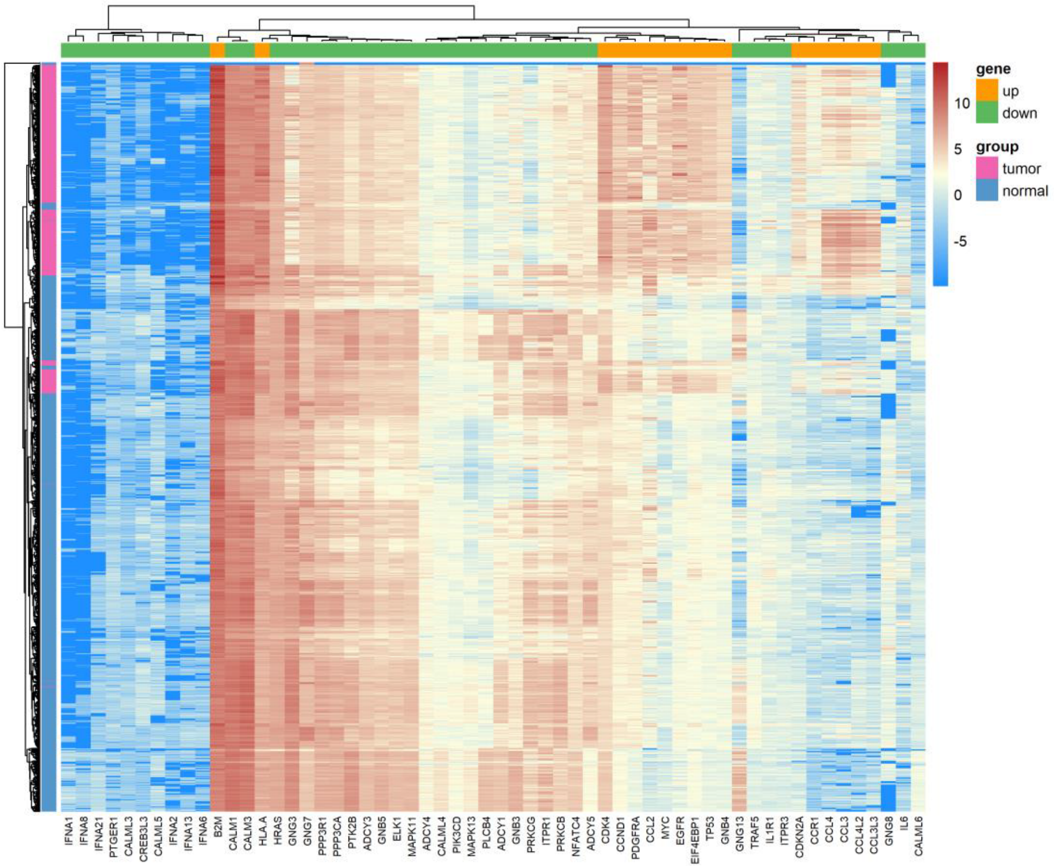

gene
up
down
group
tumor
normal
10
5
0
-5
IFNA1
IFNA8
IFNA21
PTGER1
CALML3
CREB3L3
CALML5
IFNA2
IFNA13
IFNA6
B2M
CALM1
CALM3
HLA.A
HRAS
GNG3
GNG7
PPP3R1
PPP3CA
PTK2B
ADCY3
GNB5
ELK1
MAPK11
ADCY4
CALML4
PIK3CD
MAPK13
PLCB4
ADCY1
GNB3
PRKCG
ITPR1
PRKCB
NFATC4
ADCY5
CDK4
CCND1
PDGFRA
CCL2
MYC
EGFR
EIF4EBP1
TP53
GNB4
GNG13
TRAF5
IL1R1
ITPR3
CDKN2A
CCR1
CCL4
CCL3
CCL4L2
CCL3L3
GNG8
IL6
CALML6

N

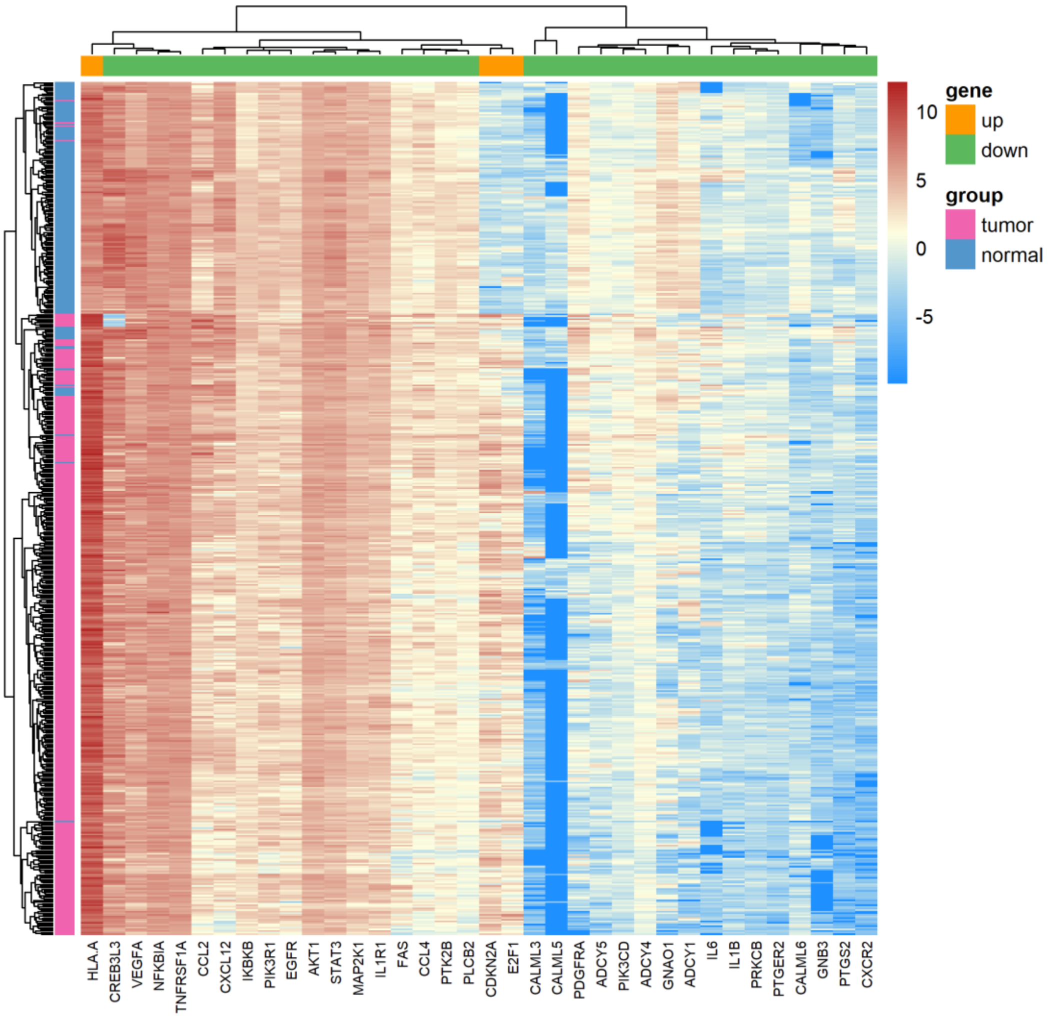

gene
up
down
group
tumor
normal
10
5
0
-5
HLA.A
CREB3L3
VEGFA
NFKBIA
TNFRSF1A
CCL2
CXCL12
IKBKB
PIK3R1
EGFR
AKT1
STAT3
MAP2K1
IL1R1
FAS
CCL4
PTK2B
PLCB2
CDKN2A
E2F1
CALML3
CALML5
PDGFRA
ADCY5
PIK3CD
ADCY4
GNAO1
ADCY1
IL6
IL1B
PRKCB
PTGER2
CALML6
GNB3
PTGS2
CXCR2

O

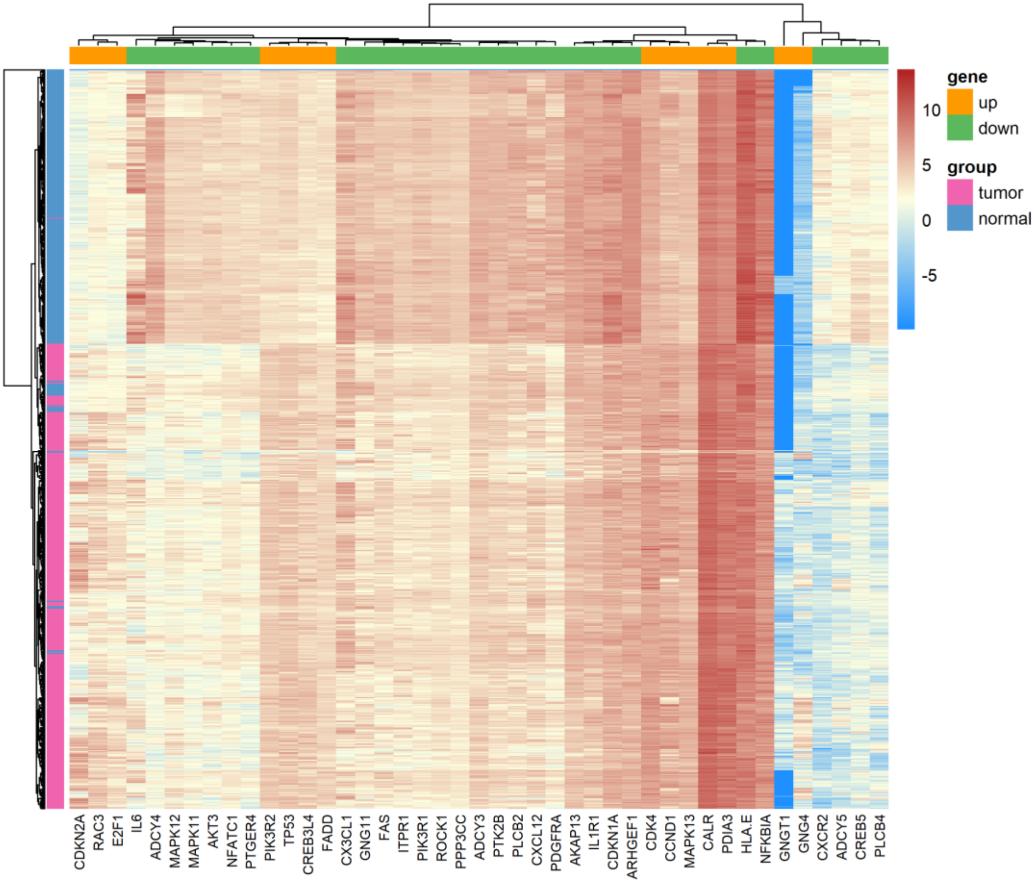

gene
up
down
group
tumor
normal
10
5
0
-5
CDKN2A
RAC3
E2F1
IL6
ADCY4
MAPK12
MAPK11
AKT3
NFATC1
PTGER4
PIK3R2
TP53
CREB3L4
FADD
CX3CL1
GNG11
FAS
ITPR1
PIK3R1
ROCK1
PPP3CC
ADCY3
PTK2B
PLCB2
CXCL12
PDGFRA
AKAP13
IL1R1
CDKN1A
ARHGEF1
CDK4
CCND1
MAPK13
CALR
PDIA3
HLA.E
NFKBIA
GNGT1
GNG4
CXCR2
ADCY5
CREB5
PLCB4

P

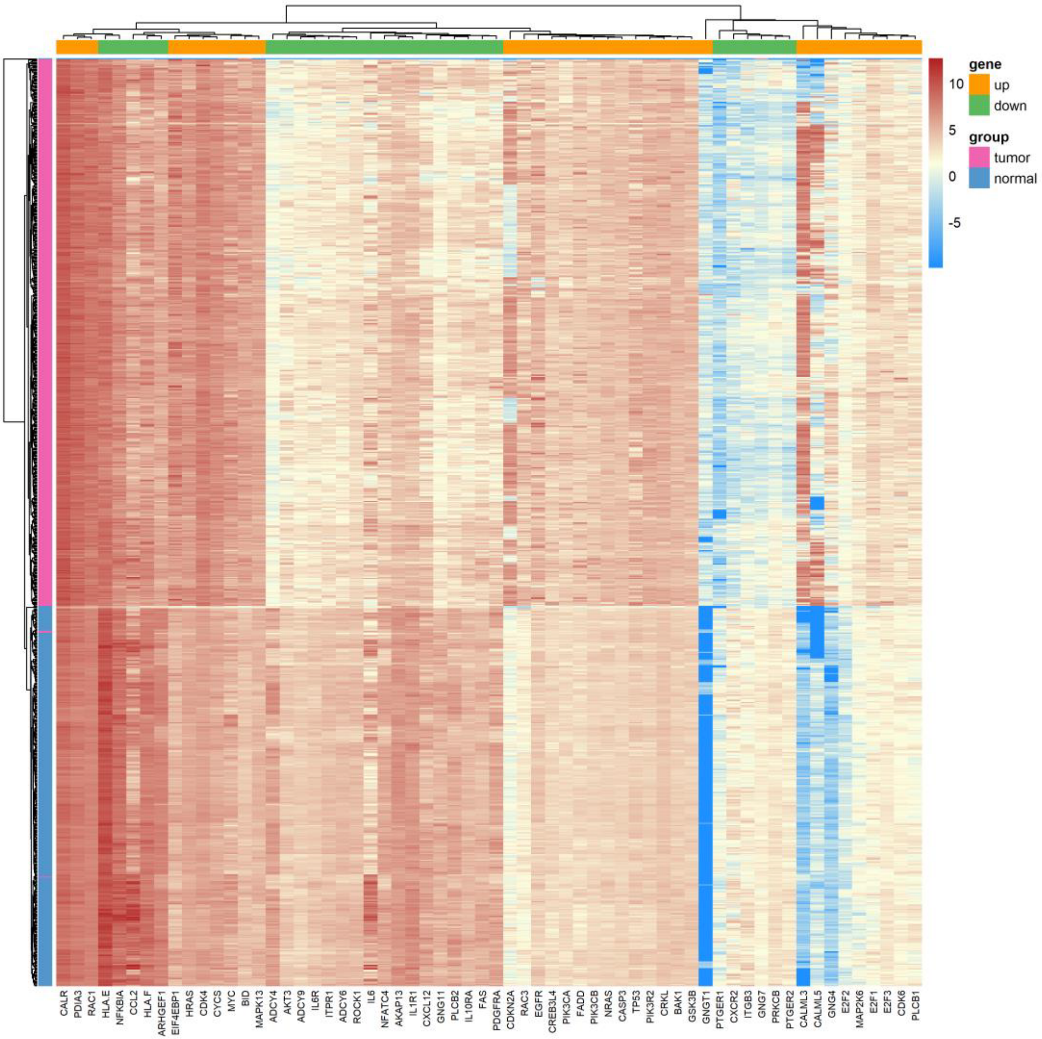
gene
up
down
group
tumor
normal
10
5
0
-5
CALR
PDIA3
RAC1
HLA.E
NFKBIA
CCL2
HLA.F
ARHGEF1
EIF4EBP1
HRAS
CDK4
CYCS
MYC
BID
MAPK13
ADCY4
AKT3
ADCY9
IL6R
ITPR1
ADCY6
ROCK1
IL6
NFATC4
AKAP13
IL1R1
CXCL12
GNG11
PLCB2
IL10RA
FAS
PDGFRA
CDKN2A
RAC3
EGFR
CREB3L4
PIK3CA
FADD
PIK3CB
NRAS
CASP3
TP53
PIK3R2
CRKL
BAK1
GSK3B
GNGT1
PTGER1
CXCR2
ITGB3
GNG7
PRKCB
PTGER2
CALML3
CALML5
GNG4
E2F2
MAP2K6
E2F1
E2F3
CDK6
PLCB1

Q

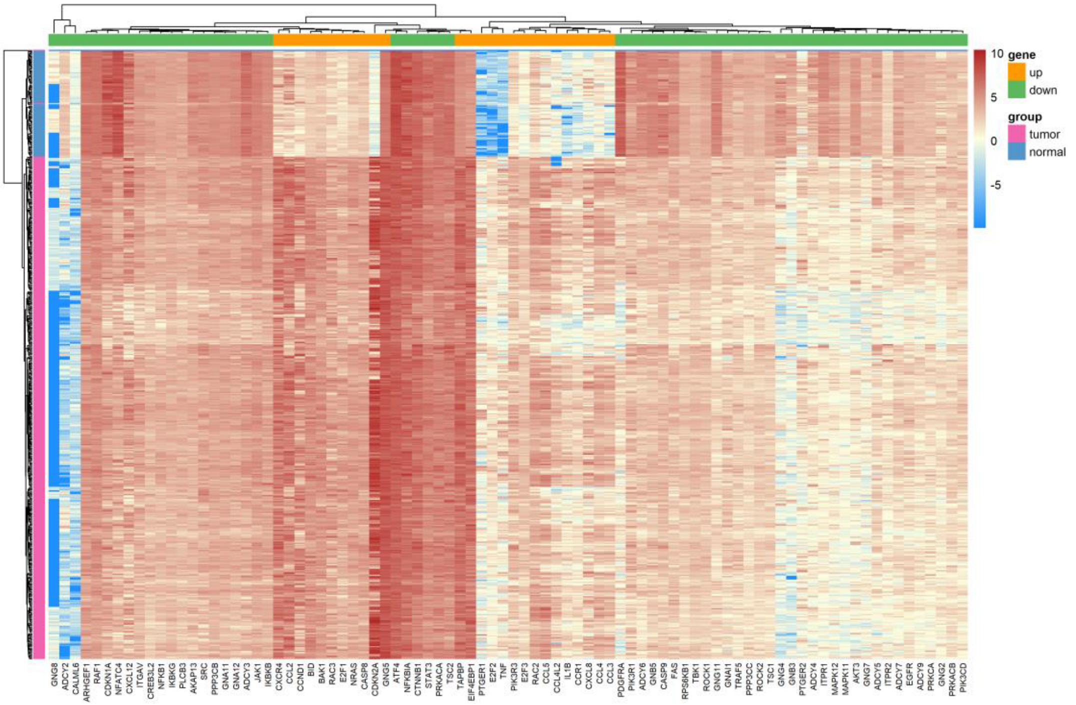
10
5
0
-5
gene
up
down
group
tumor
normal

R

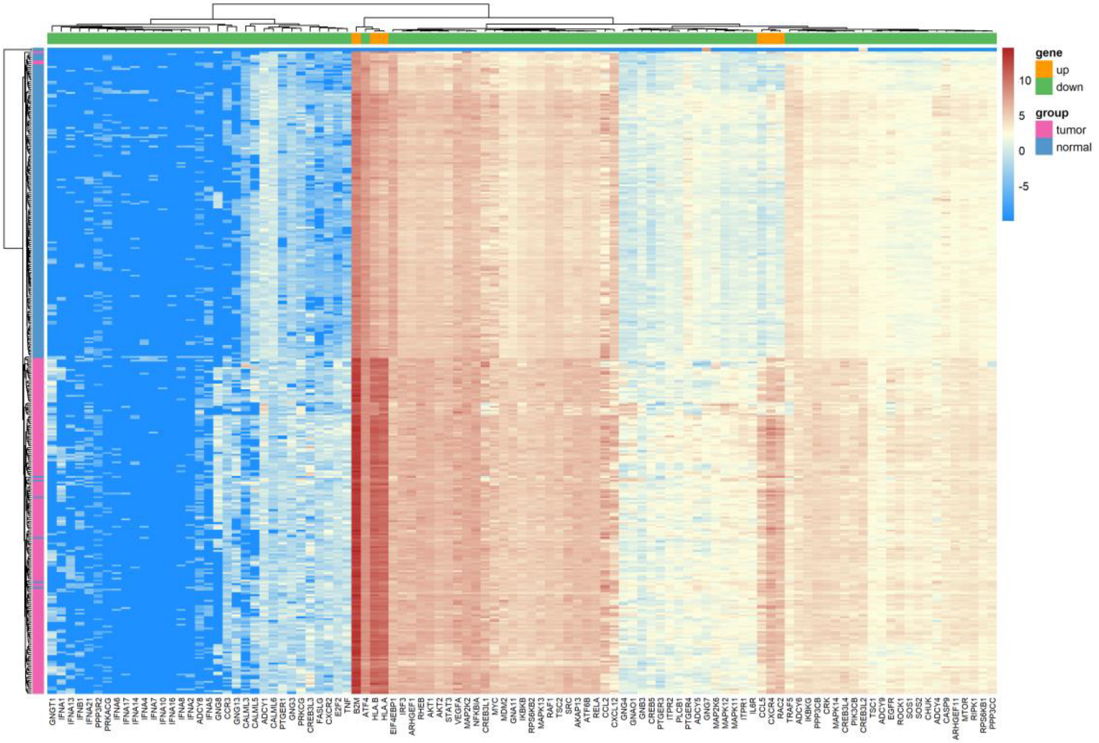
10
5
0
-5
gene
up
down
group
tumor
normal

S

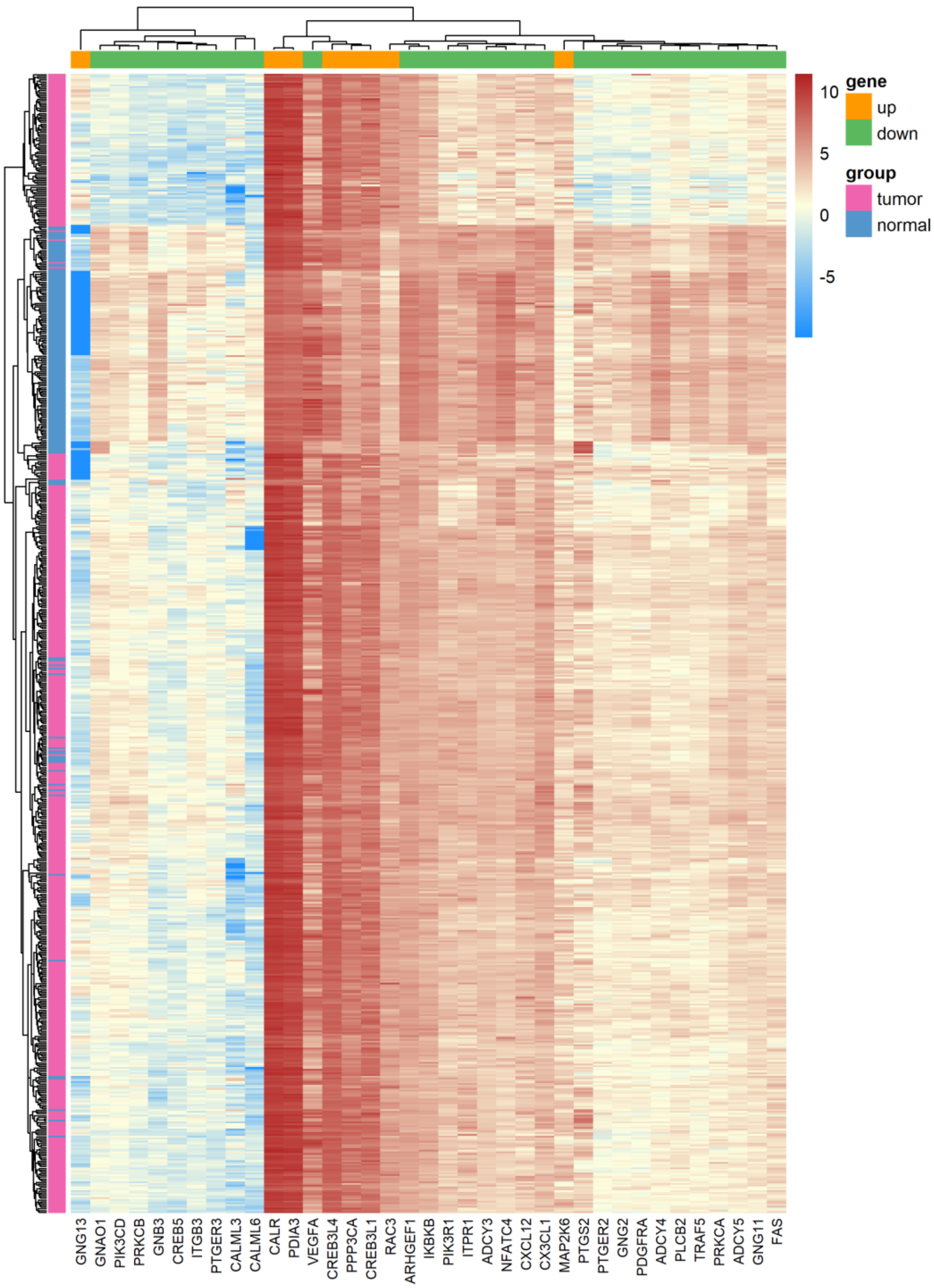
gene
up
down
group
tumor
normal
10
5
0
-5
GNG13
GNAO1
PIK3CD
PRKCB
GNB3
CREB5
ITGB3
PTGER3
CALML3
CALML6
CALR
PDIA3
VEGFA
CREB3L4
PPP3CA
CREB3L1
RAC3
ARHGEF1
IKBKB
PIK3R1
ITPR1
ADCY3
NFATC4
CXCL12
CX3CL1
MAP2K6
PTGS2
PTGER2
GNG2
PDGFRA
ADCY4
PLCB2
TRAF5
PRKCA
ADCY5
GNG11
FAS

T

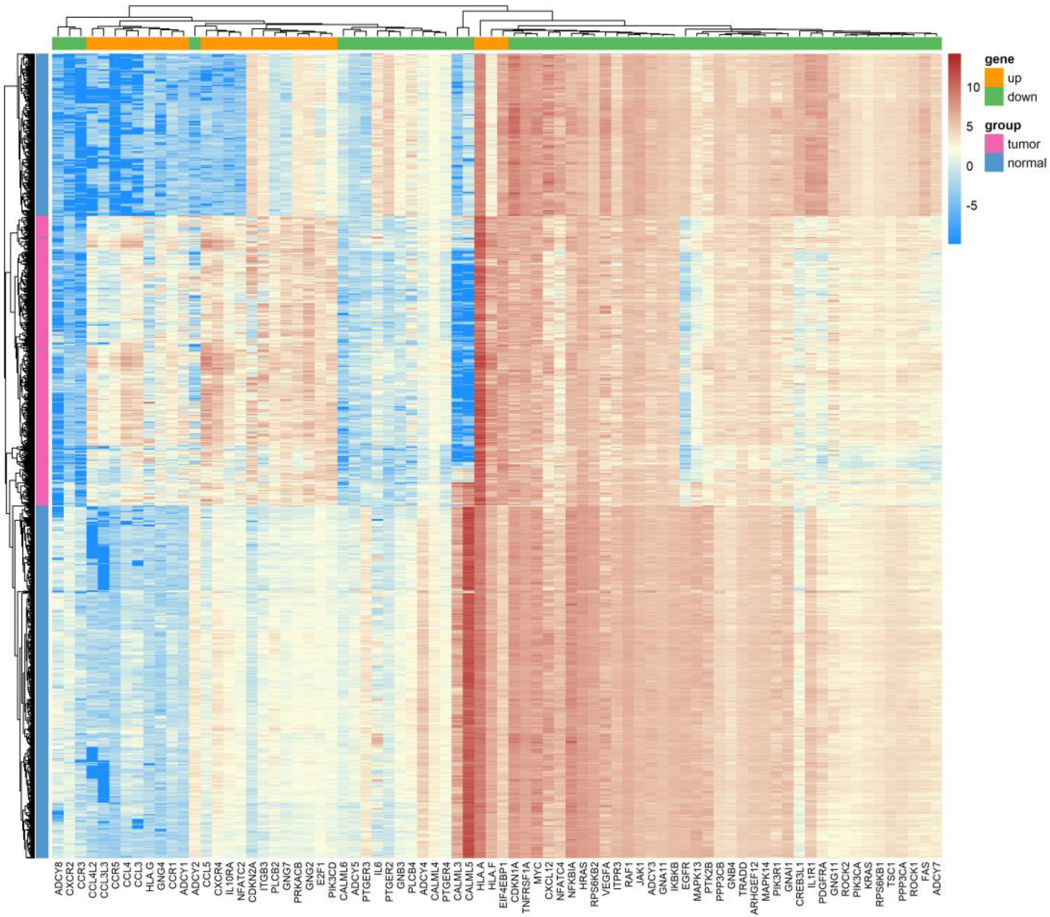
gene
up
down
group
tumor
normal
10
5
0
-5
ADCY8
CXCR2
CCR3
CCL4L2
CCL3L3
CCR5
CCL4
CCL3
HLA.G
GNG4
CCR1
ADCY1
ADCY2
CCL5
CXCR4
IL10RA
NFATC2
CDKN2A
ITGB3
PLCB2
GNG7
PRKACB
GNG2
E2F1
PIK3CD
CALML6
ADCY5
PTGER3
IL6
PTGER2
GNB3
PLCB4
ADCY4
CALML4
PTGER4
CALML3
CALML5
HLA.A
HLA.F
EIF4EBP1
CDKN1A
TNFRSF1A
MYC
CXCL12
NFATC4
NFKBIA
HRAS
RPS6KB2
VEGFA
ITPR3
RAF1
JAK1
ADCY3
GNA11
IKBKB
EGFR
MAPK13
PTK2B
PPP3CB
GNB4
TRADD
ARHGEF12
MAPK14
PIK3R1
GNAI1
CREB3L1
IL1R1
PDGFRA
GNG11
ROCK2
PIK3CA
KRAS
RPS6KB1
TSC1
PPP3CA
ROCK1
FAS
ADCY7

U

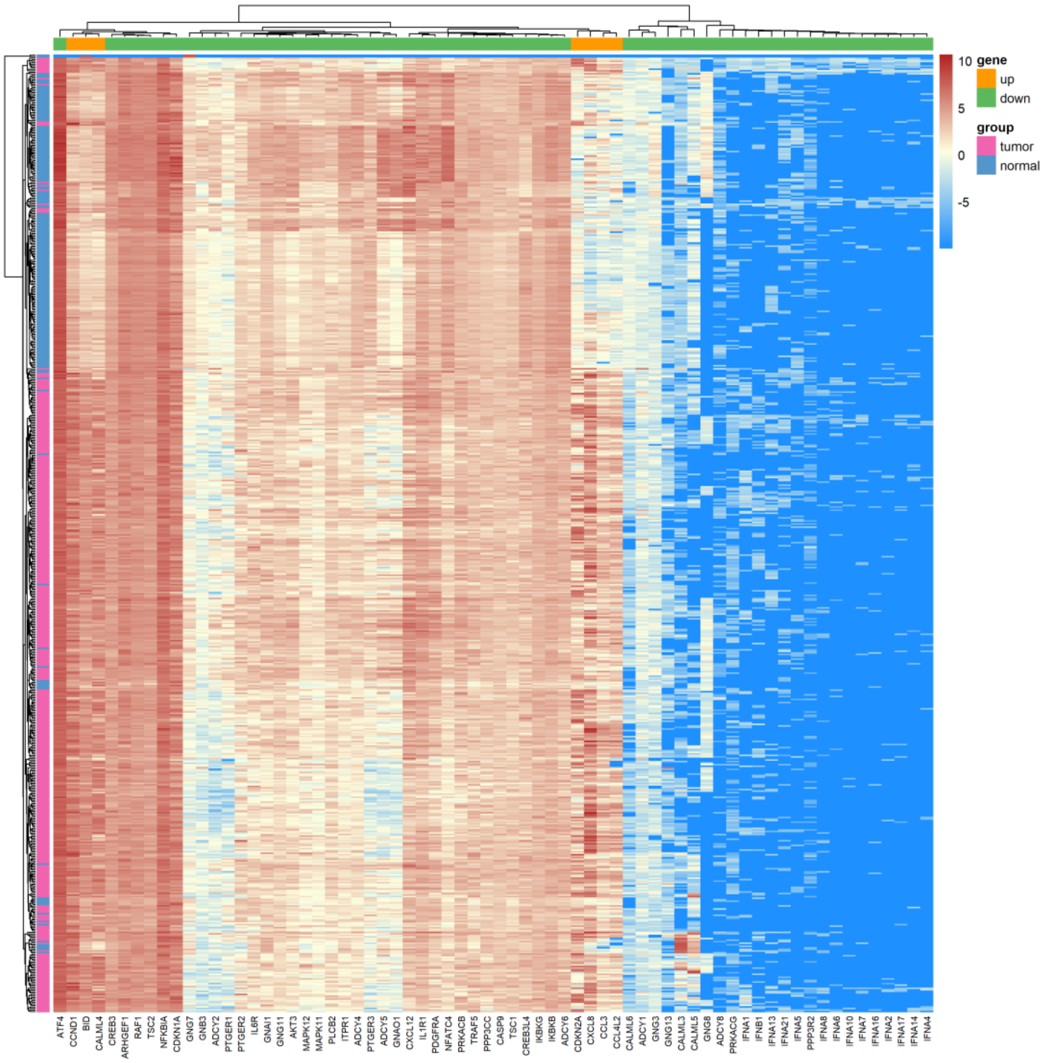
gene
up
down
group
tumor
normal
10
5
0
-5
ATF4
CCND1
BID
CALML4
CREB3
ARHGEF1
RAF1
TSC2
NFKBIA
CDKN1A
GNG7
GNB3
ADCY2
PTGER1
PTGER2
IL6R
GNAI1
GNG11
AKT3
MAPK12
MAPK11
PLCB2
ITPR1
ADCY4
PTGER3
ADCY5
GNAO1
CXCL12
IL1R1
PDGFRA
NFATC4
PRKACB
TRAF5
PPP3CC
CASP9
TSC1
CREB3L4
IKBKG
IKBKB
ADCY6
CDKN2A
CXCL8
CCL3
CCL4L2
CALML6
ADCY1
GNG3
GNG13
CALML3
CALML5
GNG8
ADCY8
PRKACG
IFNA1
IFNB1
IFNA13
IFNA21
IFNA5
PPP3R2
IFNA8
IFNA6
IFNA10
IFNA7
IFNA16
IFNA2
IFNA17
IFNA14
IFNA4

V

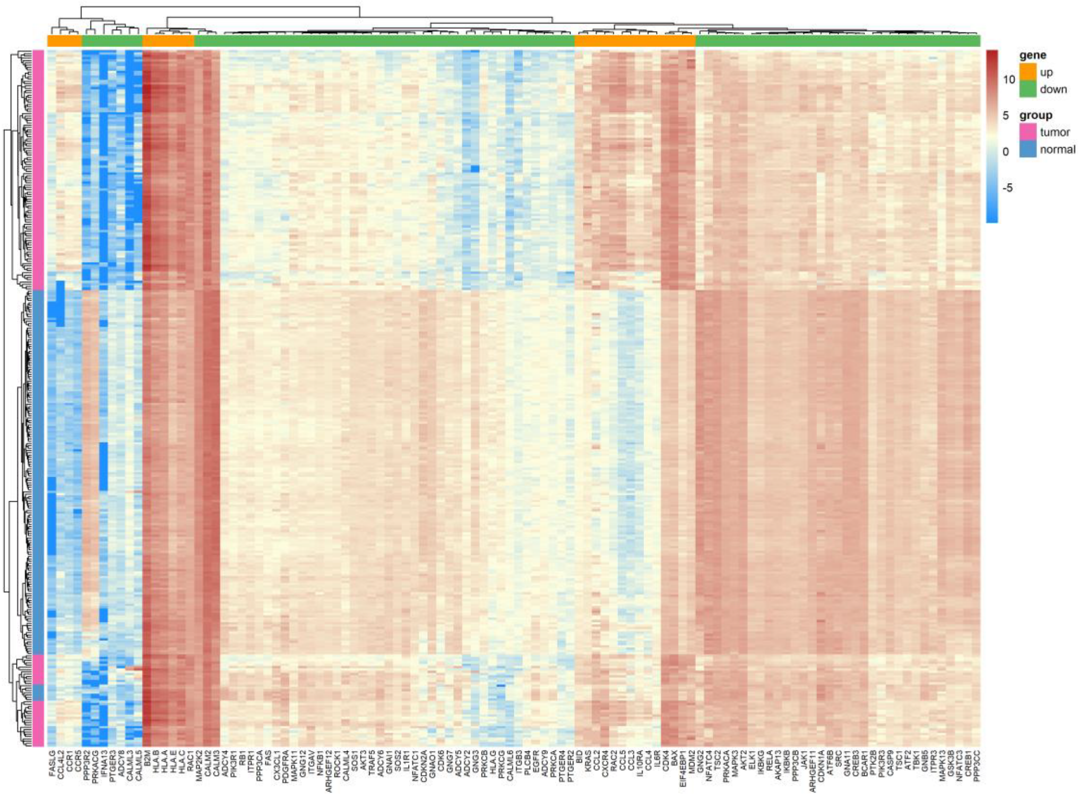

W

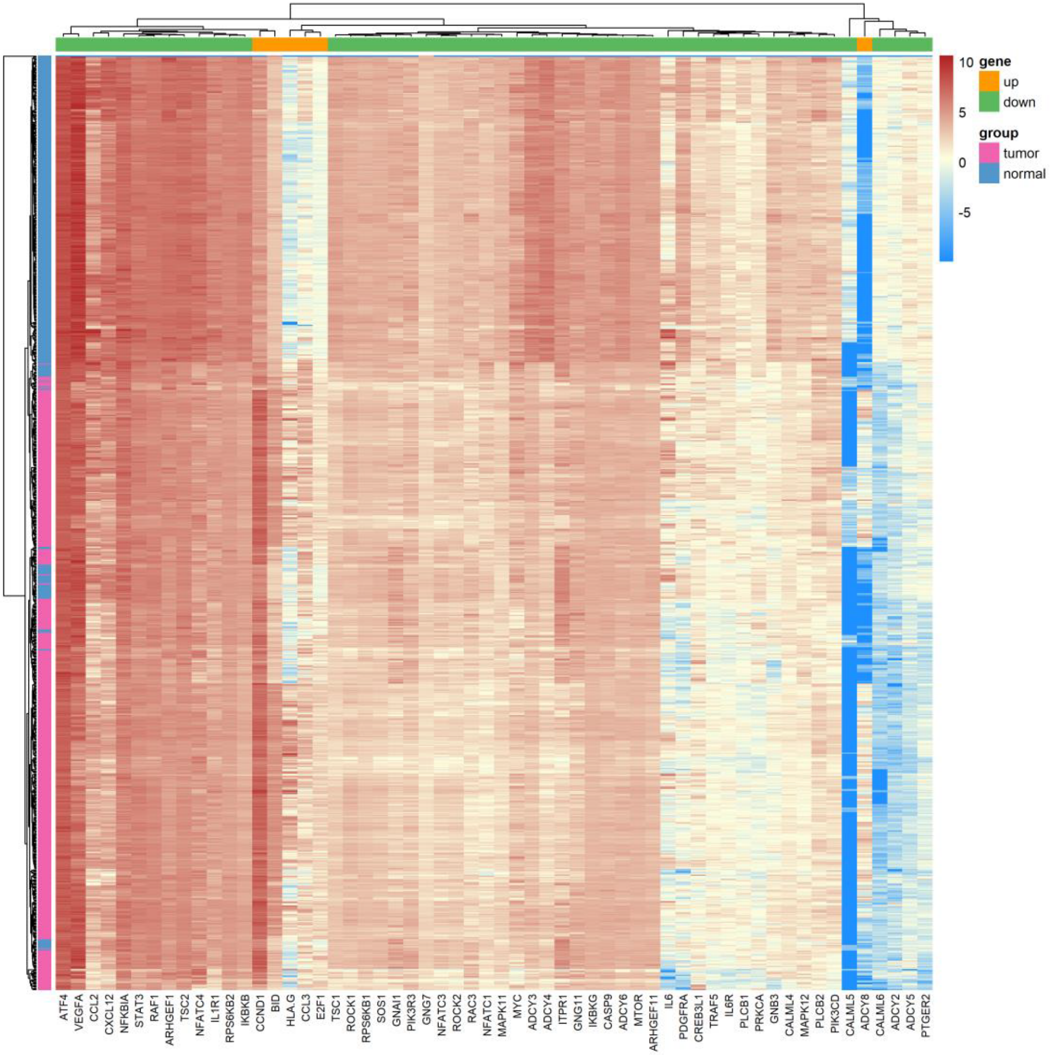

gene
up
down
group
tumor
normal
10
5
0
-5
ATF4
VEGFA
CCL2
CXCL12
NFKBIA
STAT3
RAF1
ARHGEF1
TSC2
NFATC4
IL1R1
RPS6KB2
IKBKB
CCND1
BID
HLA.G
CCL3
E2F1
TSC1
ROCK1
RPS6KB1
SOS1
GNAI1
PIK3R3
GNG7
NFATC3
ROCK2
RAC3
NFATC1
MAPK11
MYC
ADCY3
ADCY4
ITPR1
GNG11
IKBKG
CASP9
ADCY6
MTOR
ARHGEF11
IL6
PDGFRA
CREB3L1
TRAF5
IL6R
PLCB1
PRKCA
GNB3
CALML4
MAPK12
PLCB2
PIK3CD
CALML5
ADCY8
CALML6
ADCY2
ADCY5
PTGER2

X

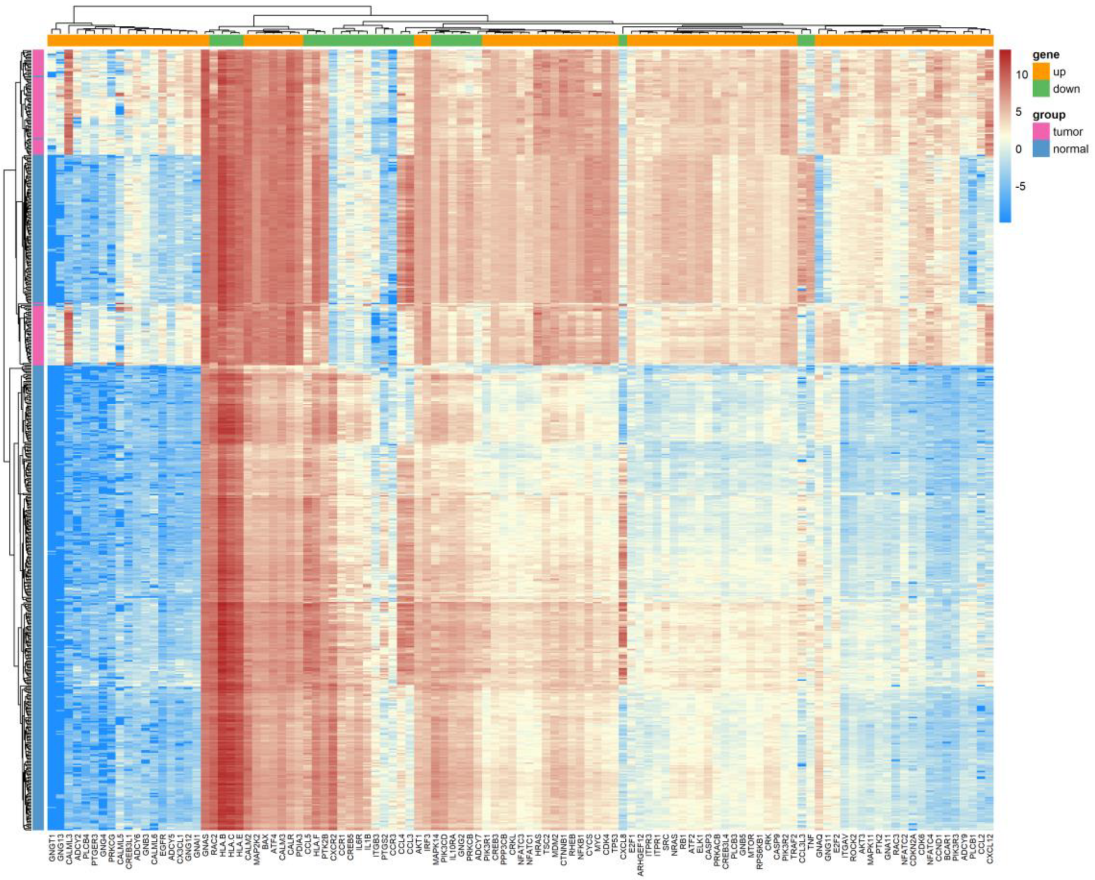
gene
up
down
group
tumor
normal
10
5
0
-5

Y

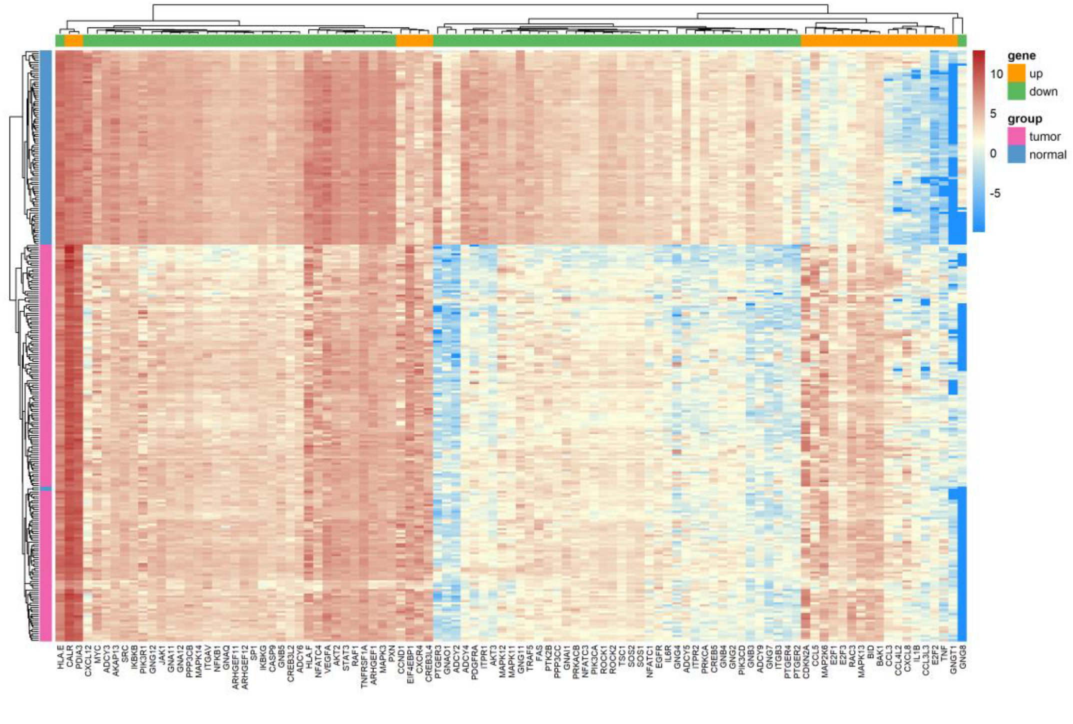
gene
up
down
group
tumor
normal
10
5
0
-5

Z

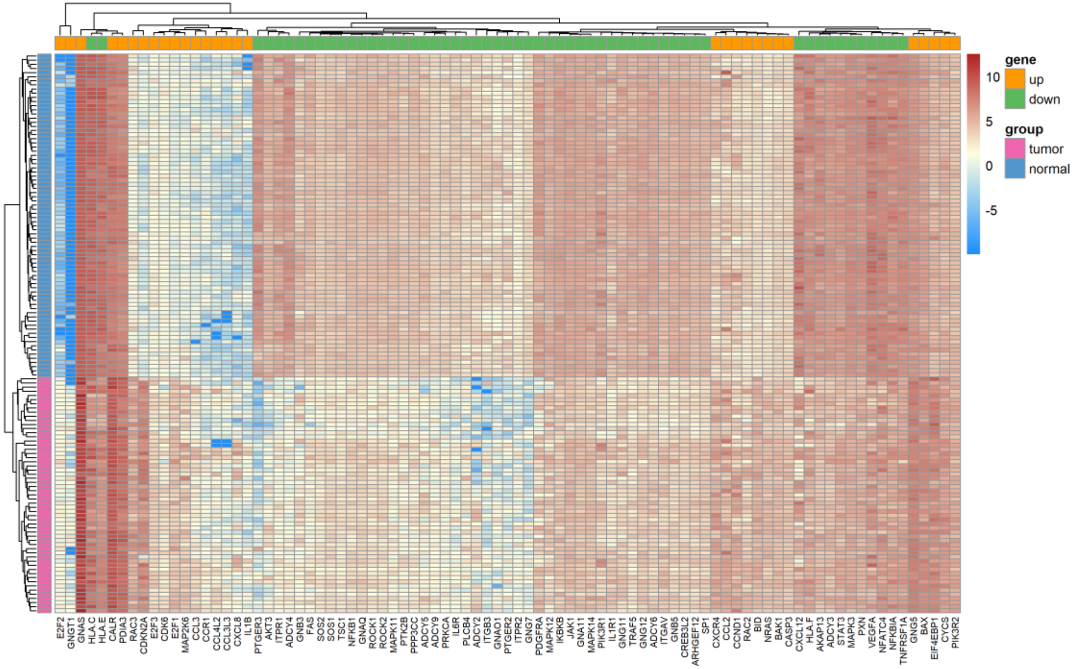

A clustering heatmap of pan-cancer. (A) ACC, (B) BLCA, (C) BRCA, (D) CHOL, (E) DLBC, (F) ESCA, (G) GBM, (H) HNSC, (I) KICH, (J) KIRC, (K) KIRP, (L) LAML, (M) LGG, (N) LIHC, (O) LUAD, (P) LUSC, (Q) OV, (R) PAAD, (S) PRAD, (T) SKCM, (U) STAD, (V) TGCT, (W) THCA, (X) THYM, (Y) UCEC, and (Z) UCS are represented in this heatmap. On the left side, pink indicates tumor samples and blue indicates normal samples; on the top, orange represents gene upregulation groups and green represents gene downregulation groups; in the heatmap, red and blue represent high and low expression levels respectively.
